# Supplementary figures and images for: Metaeffector interactions modulate the type III effector-triggered immunity load of Pseudomonas syringae
Source: PLoS Pathog. 2022 May 16;18(5):e1010541. doi: 10.1371/journal.ppat.1010541 (PMC9135338; doi:10.1371/journal.ppat.1010541)

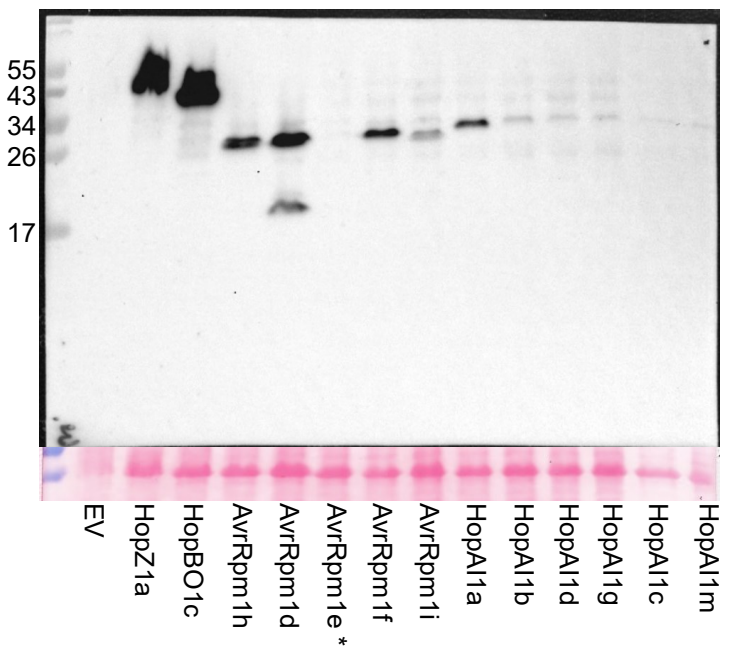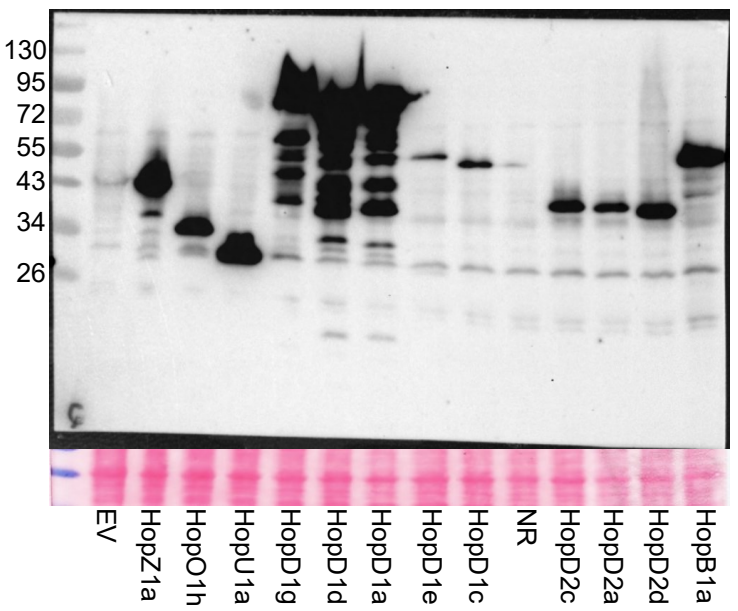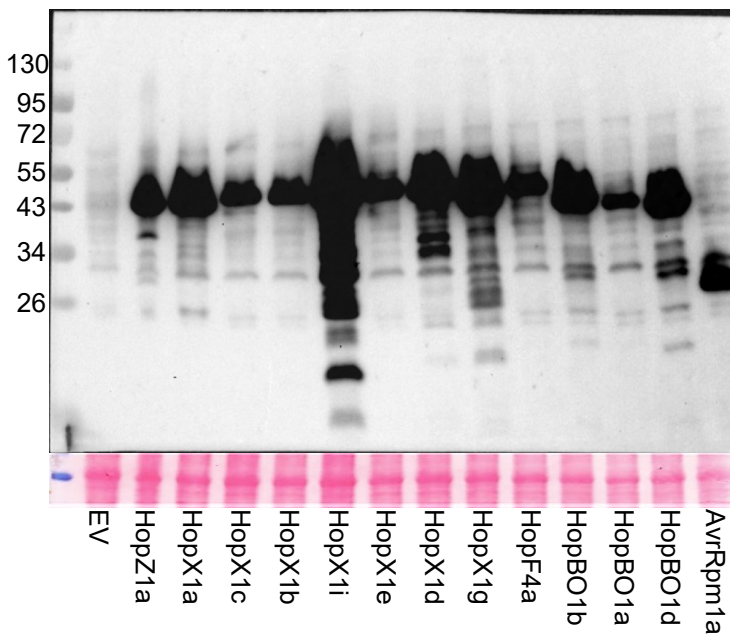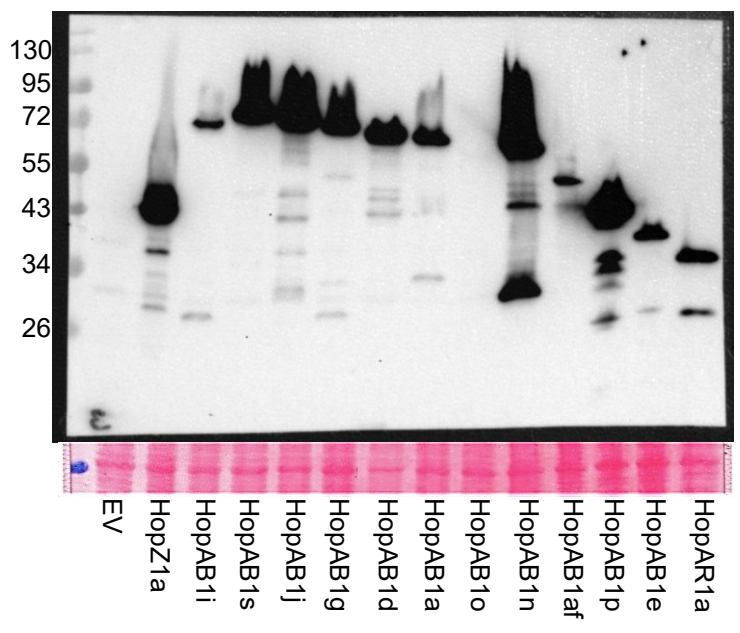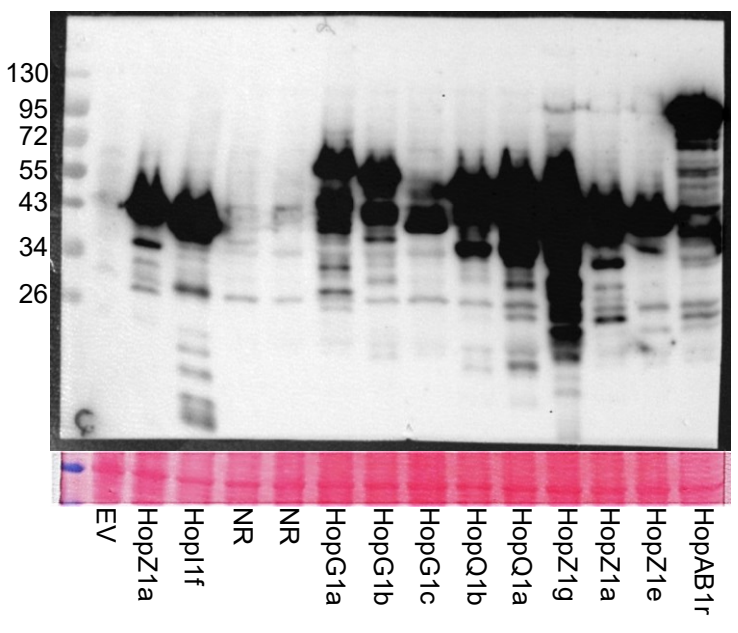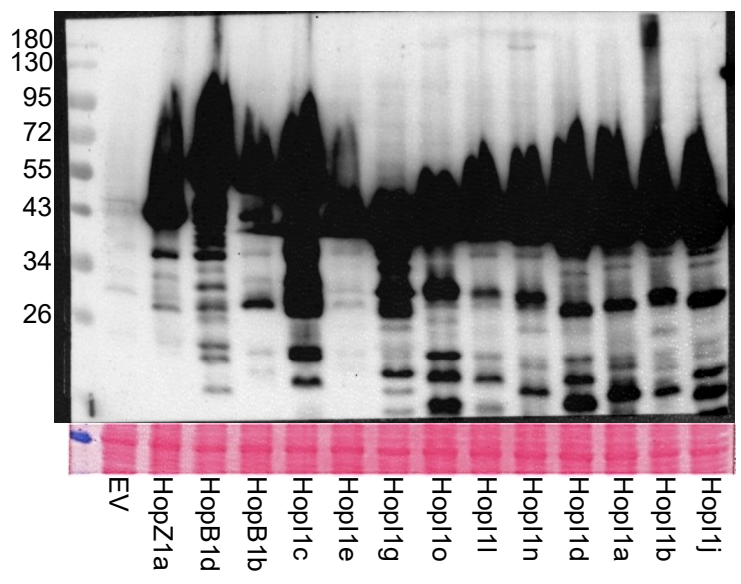

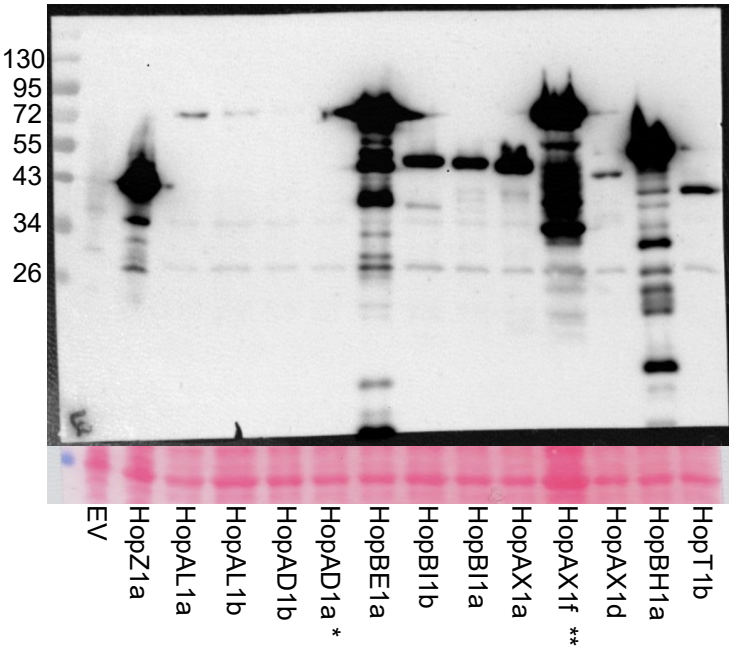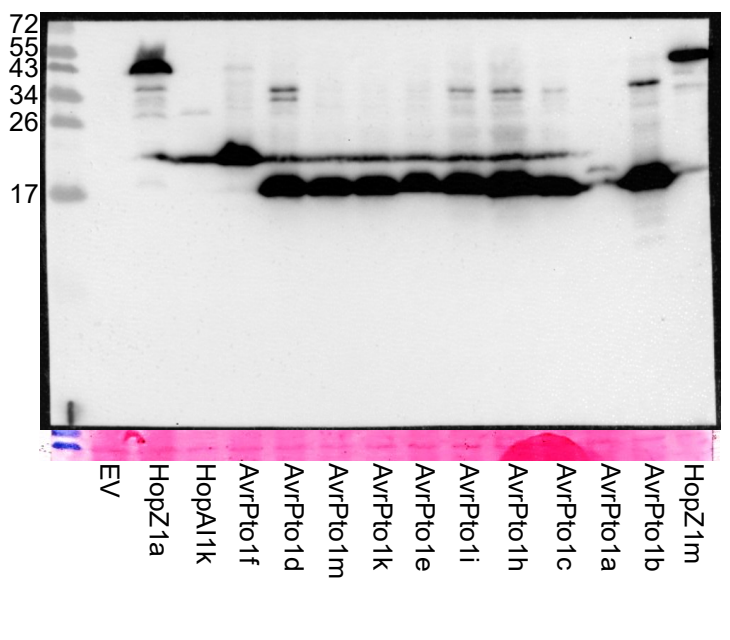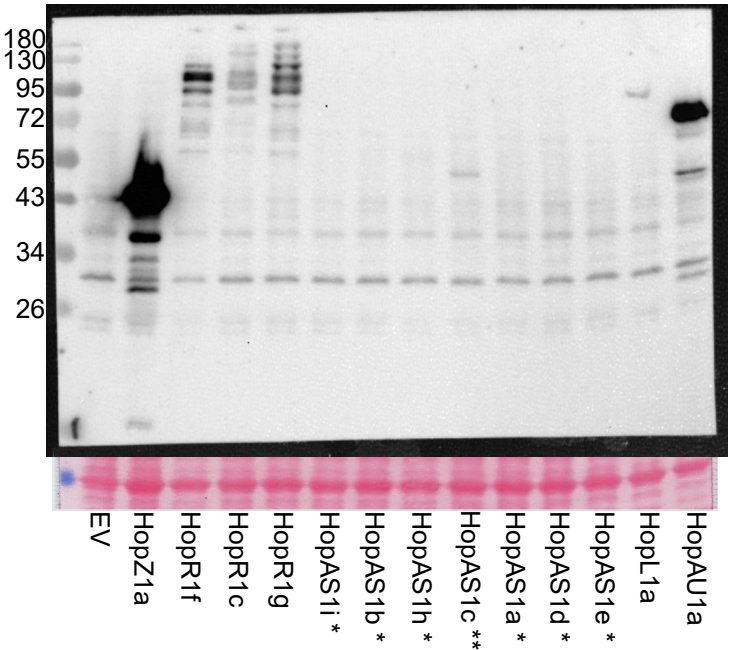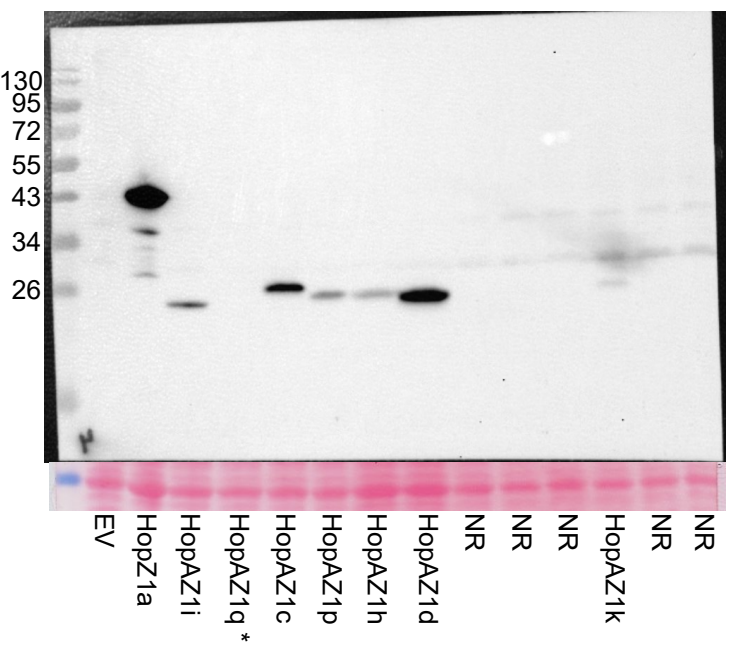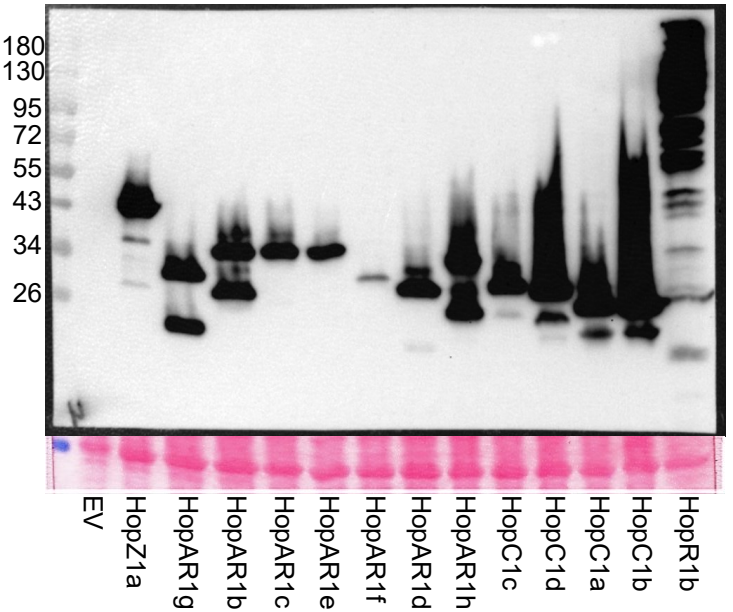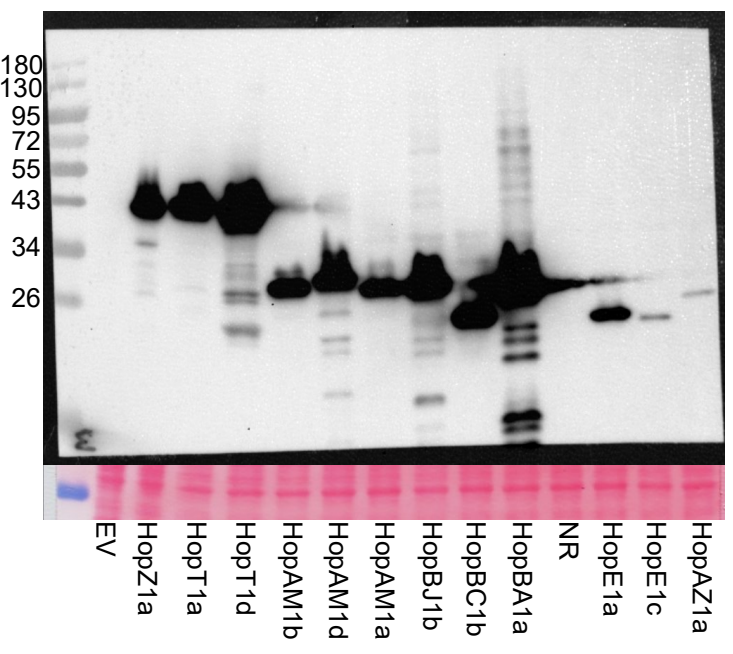

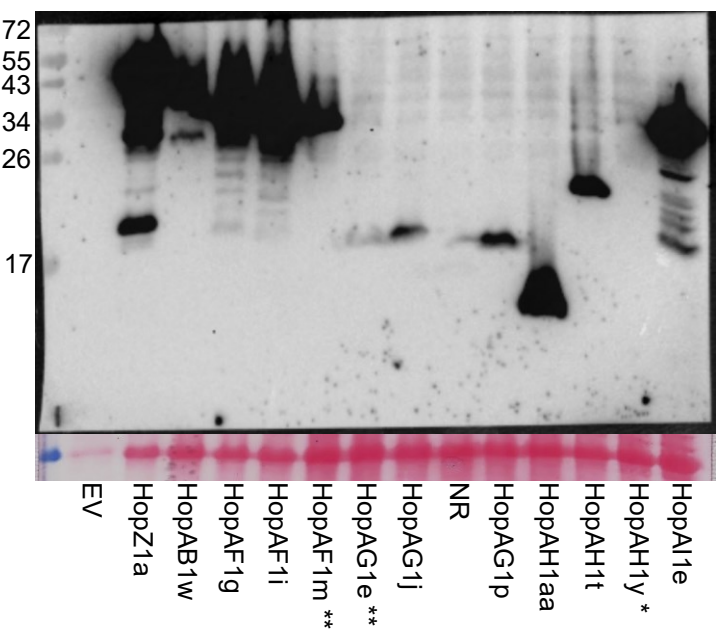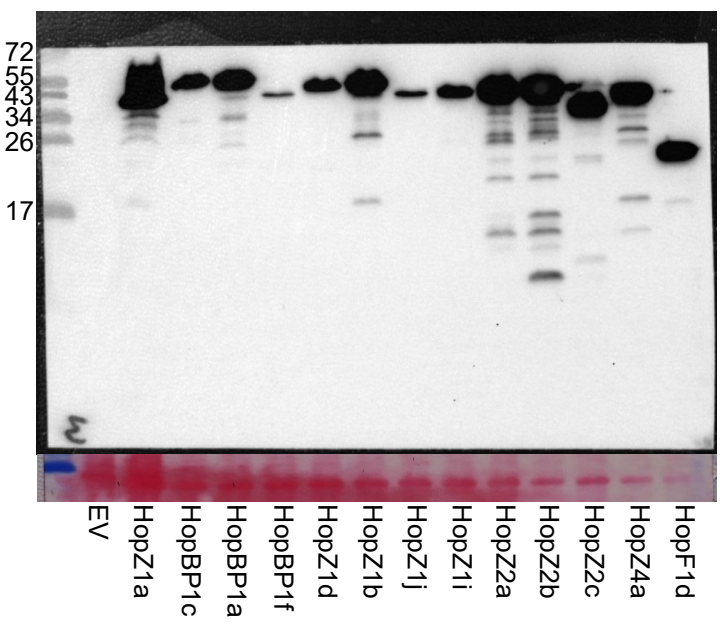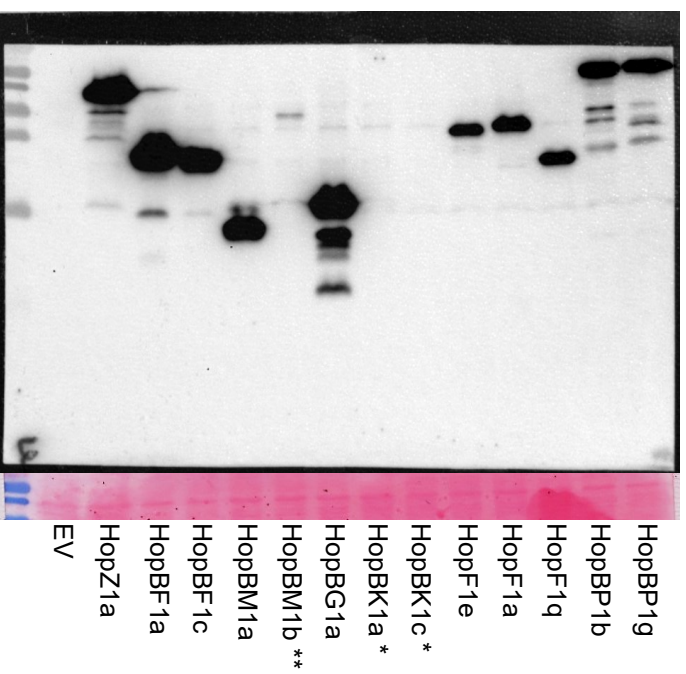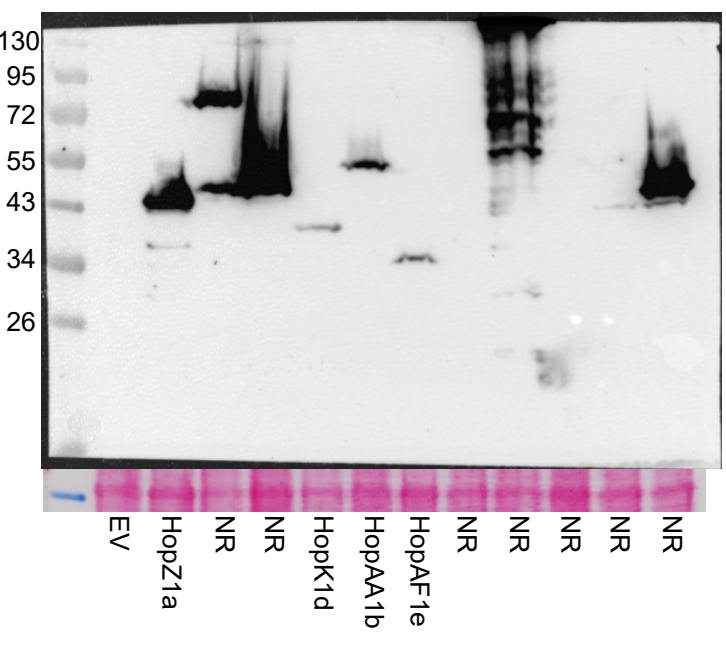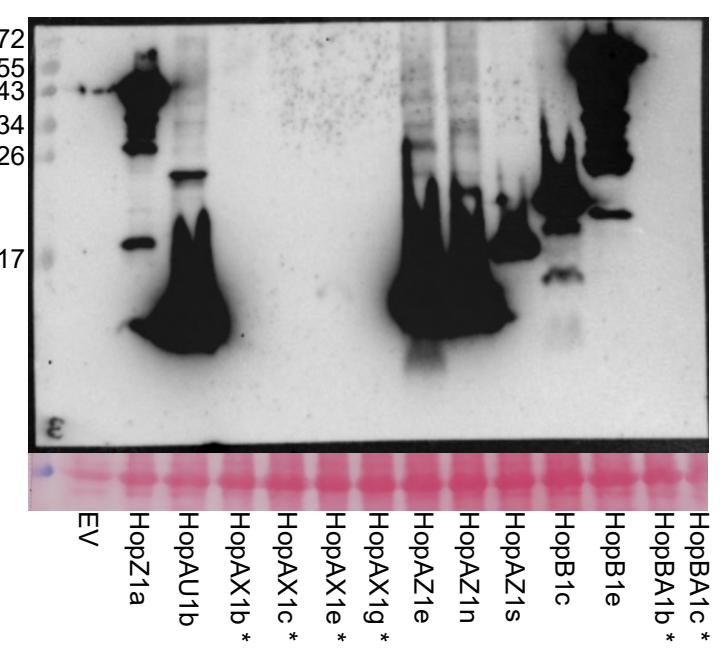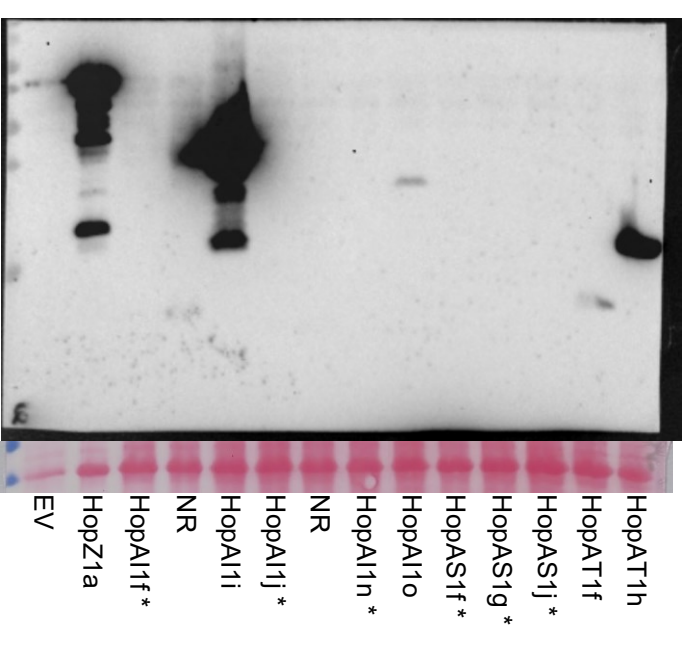

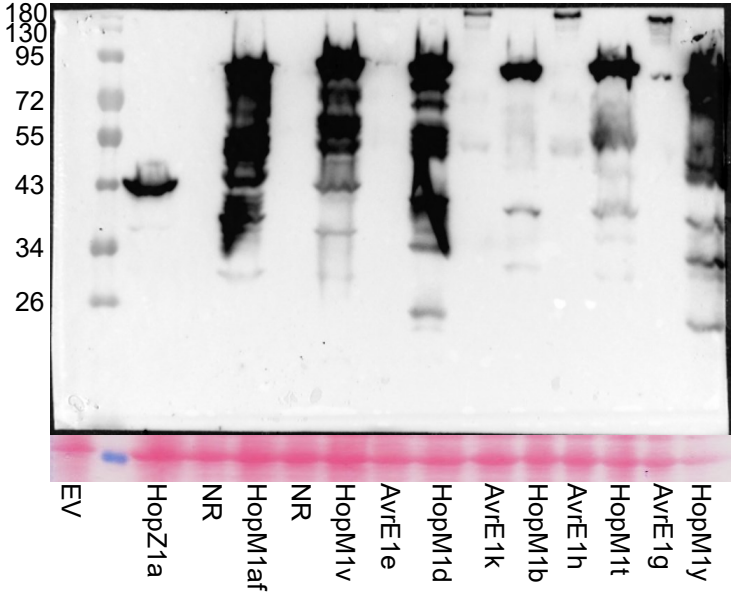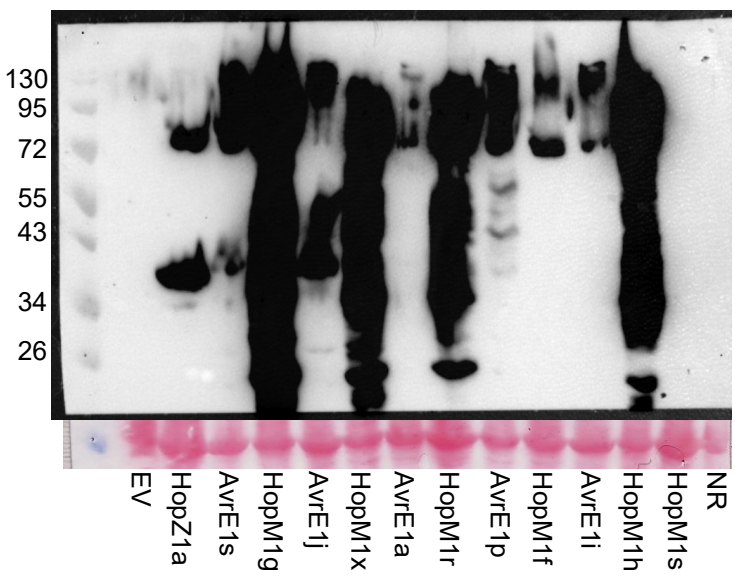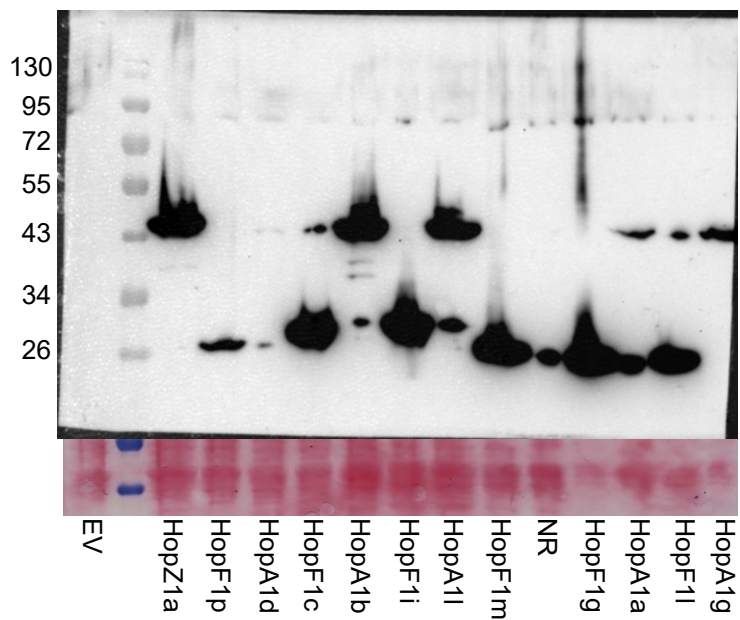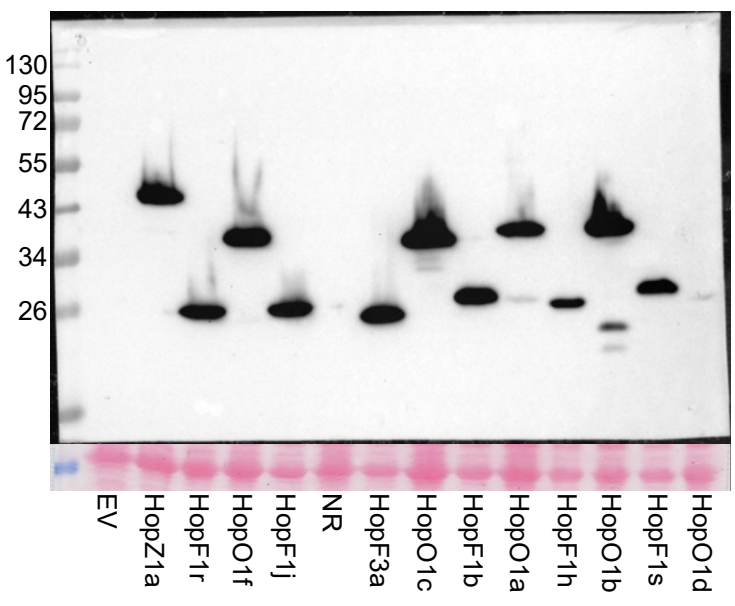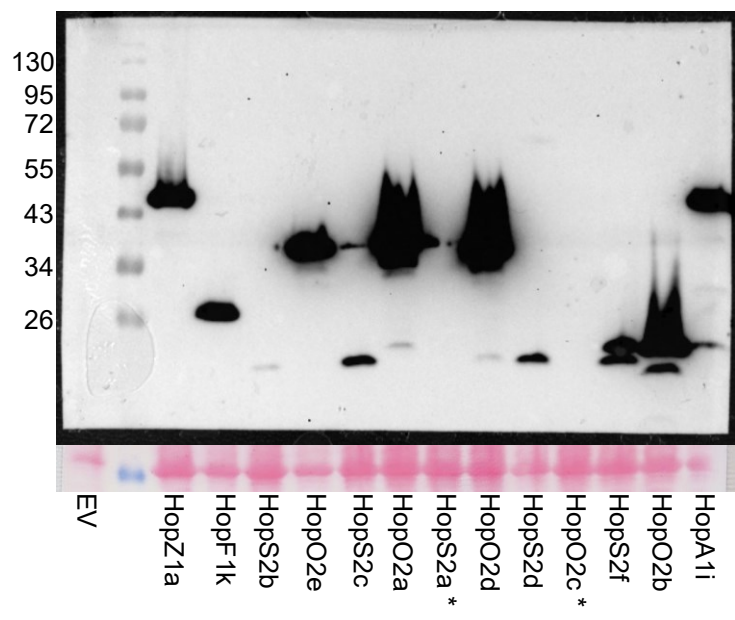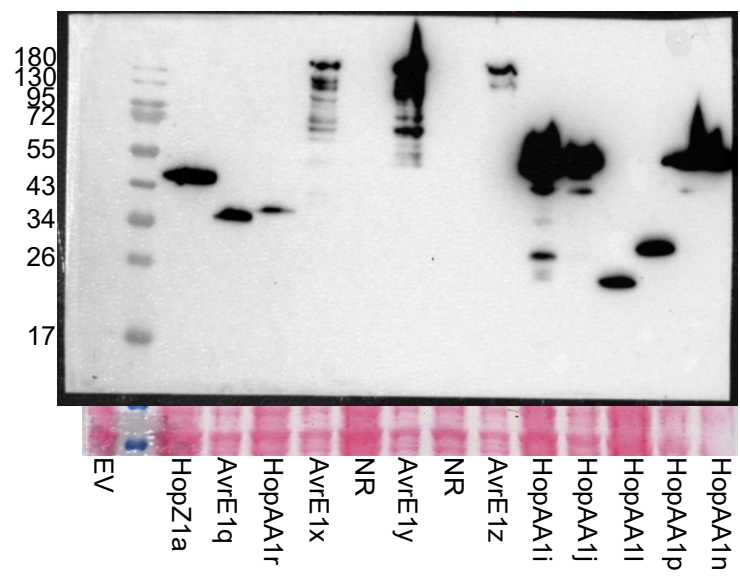

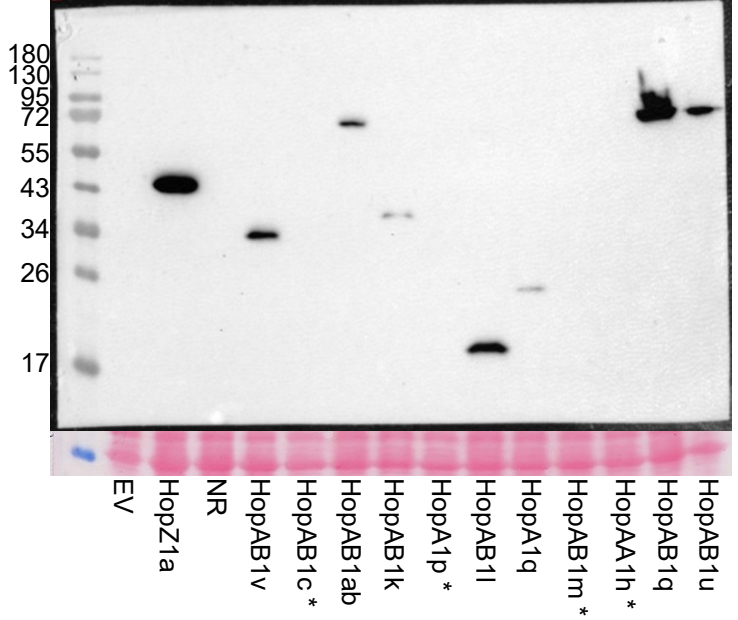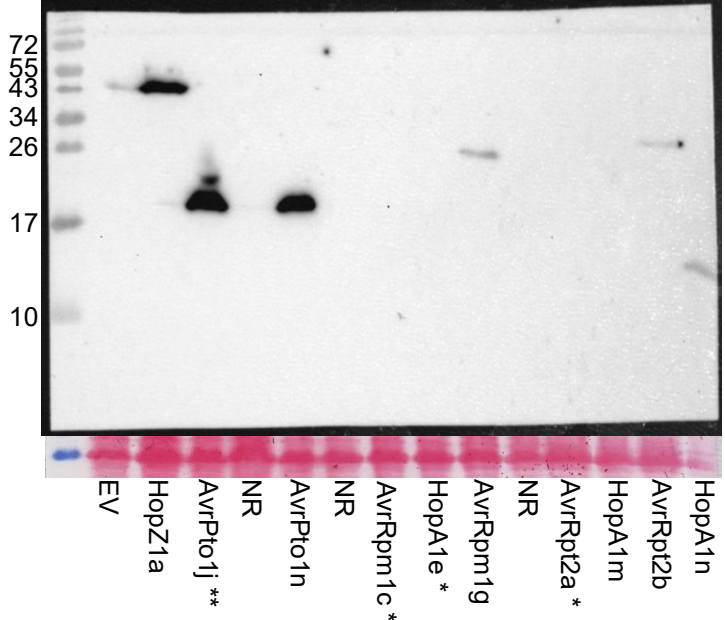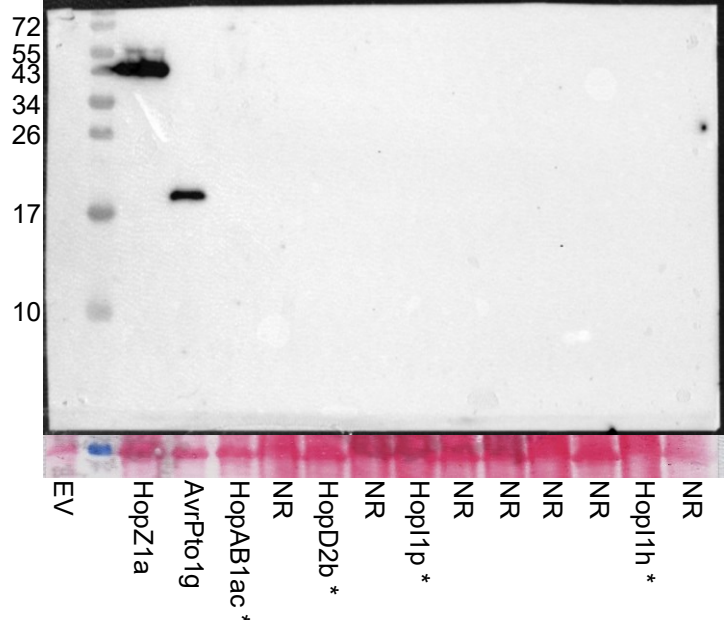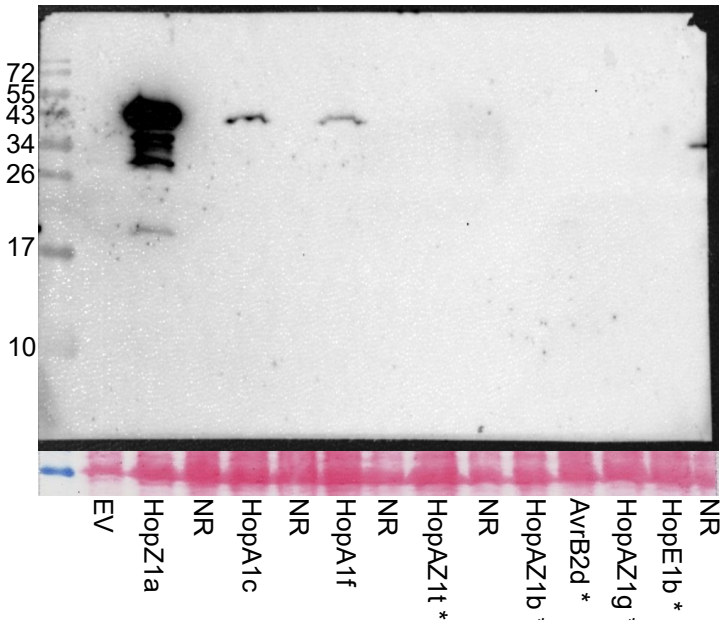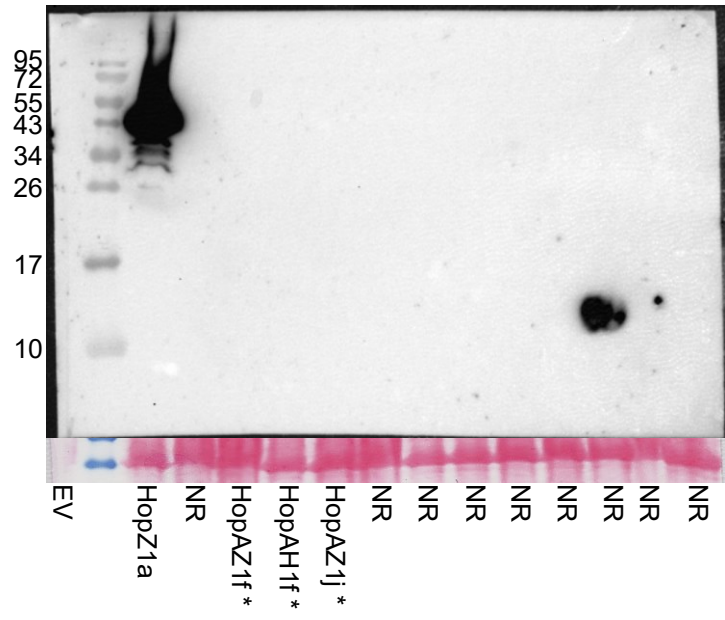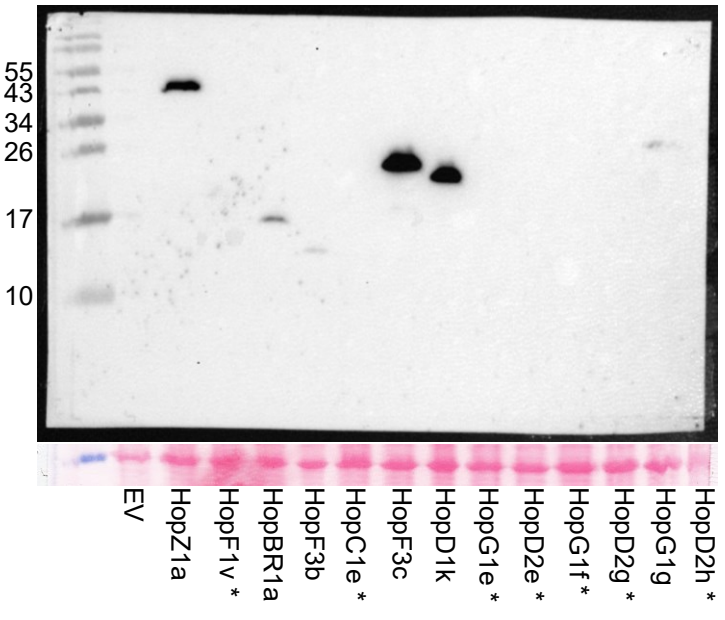

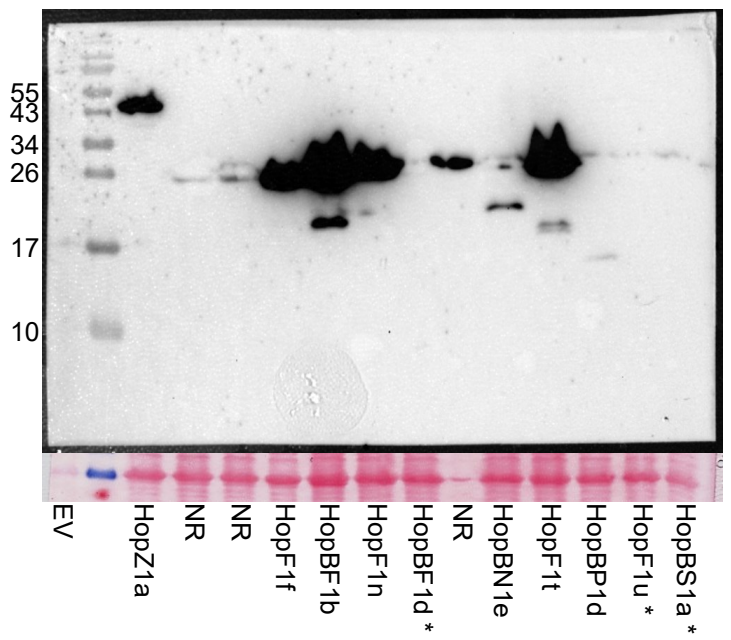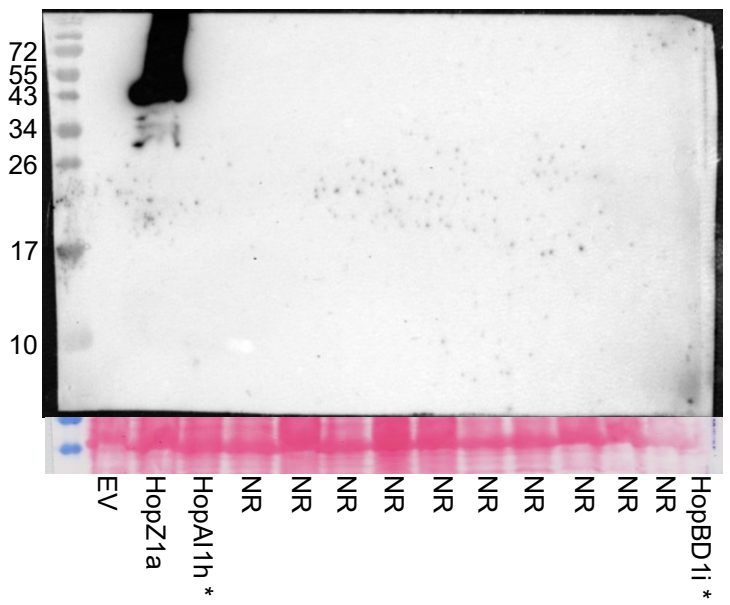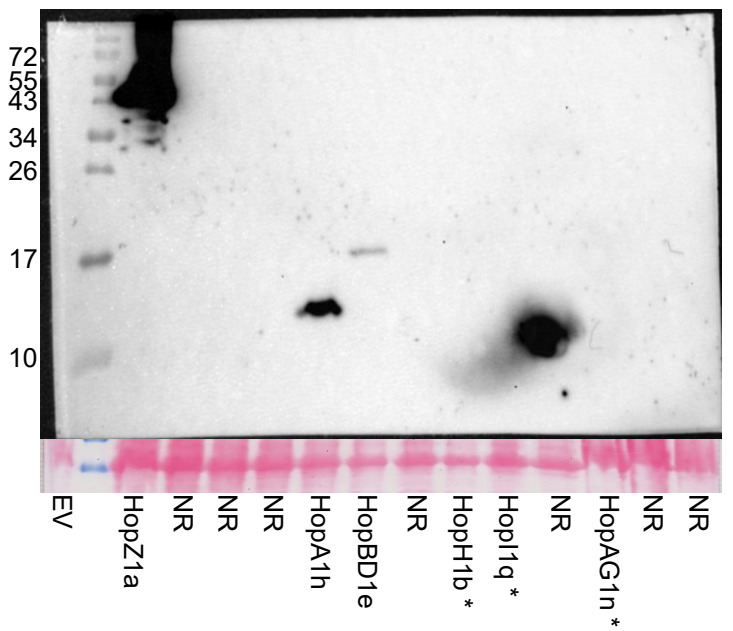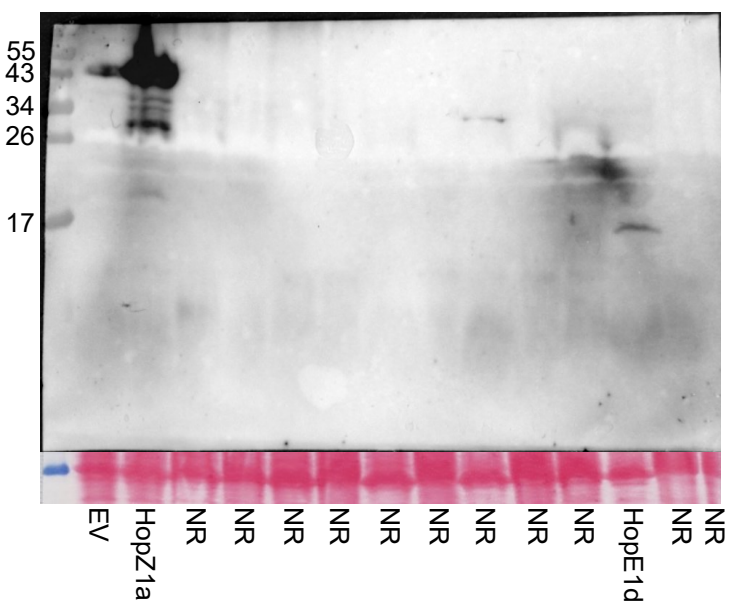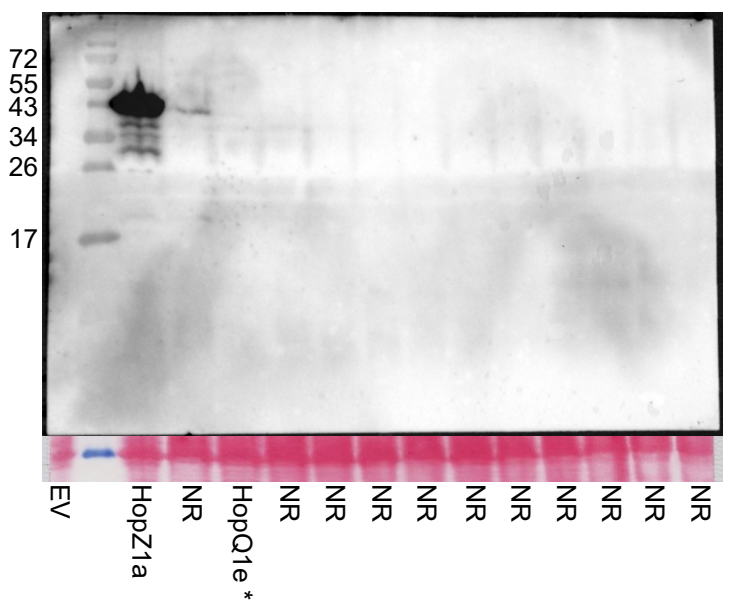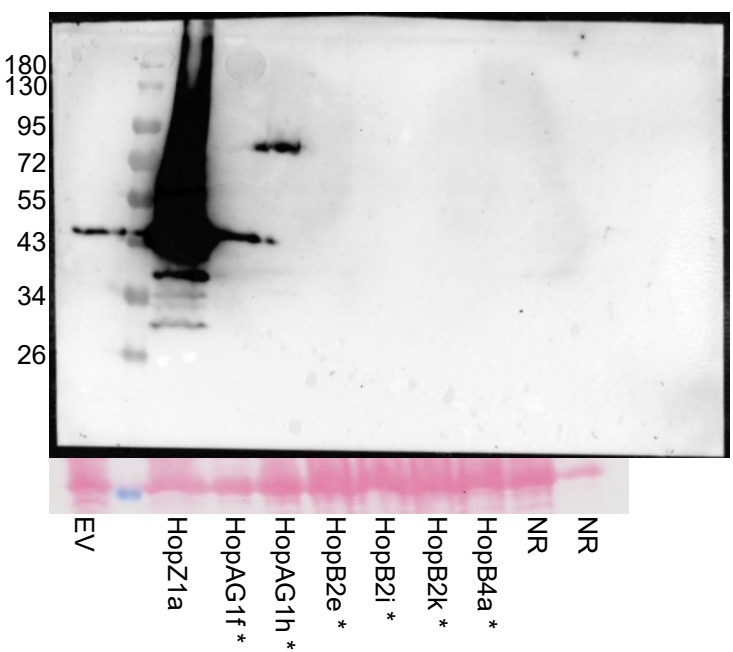

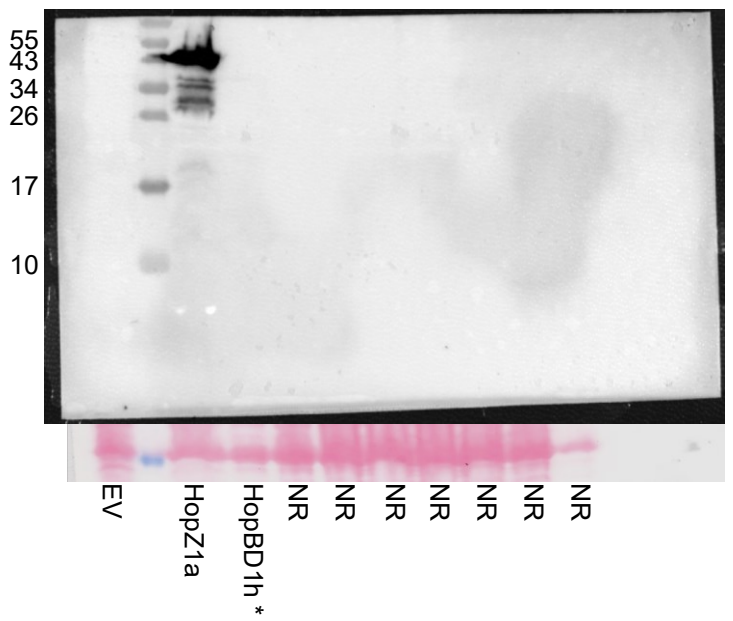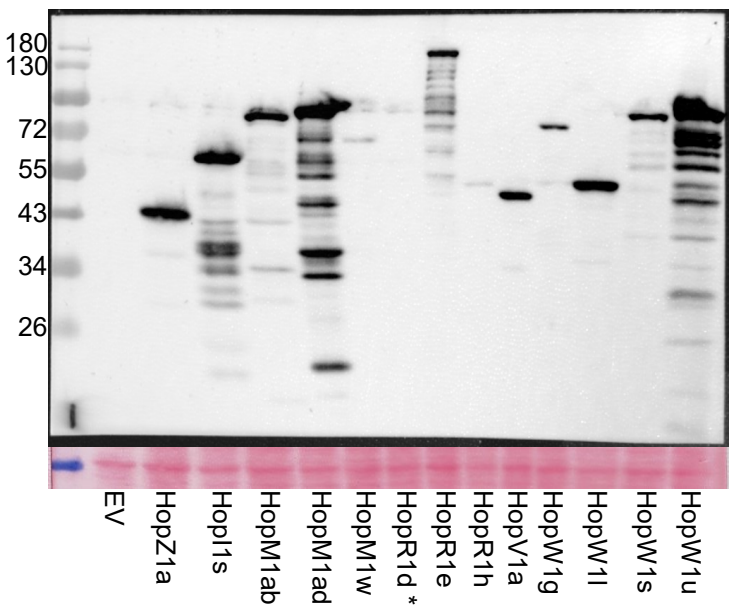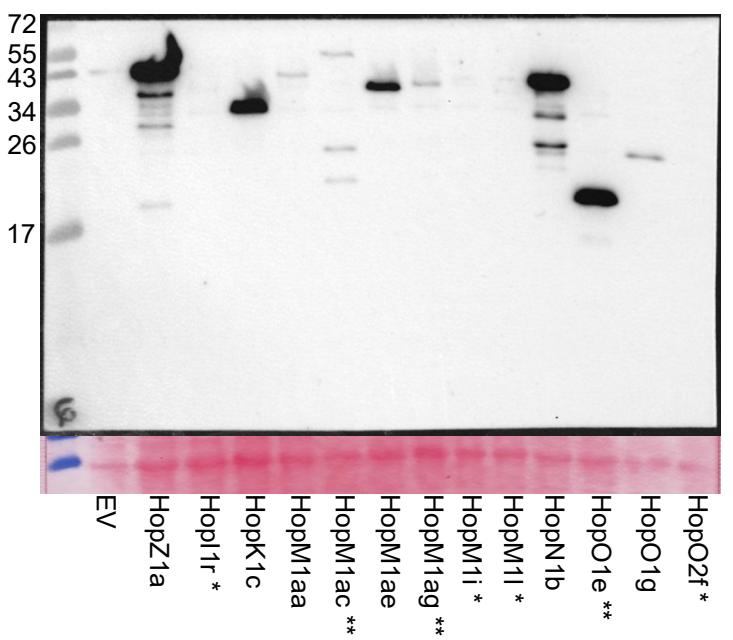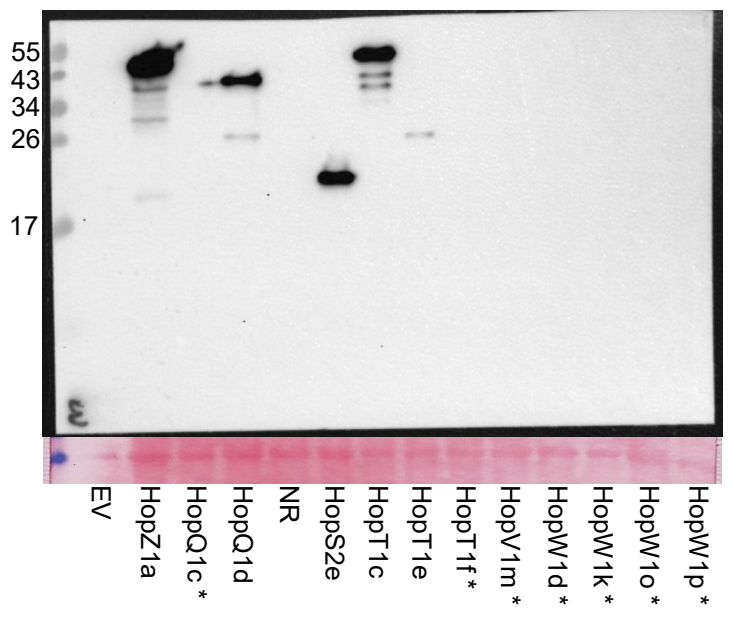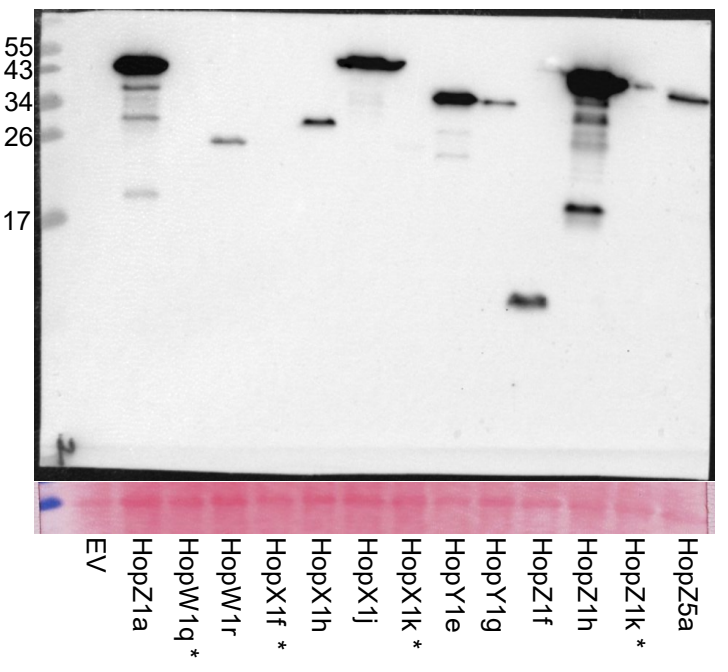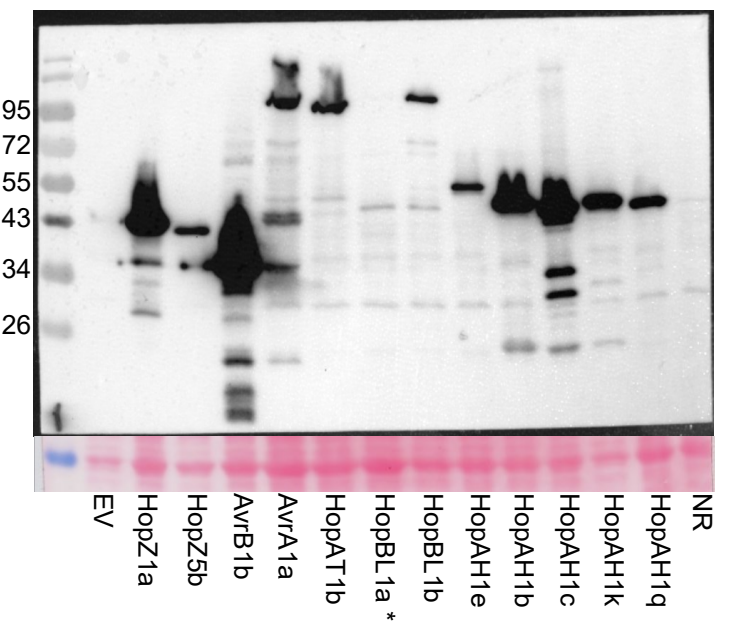

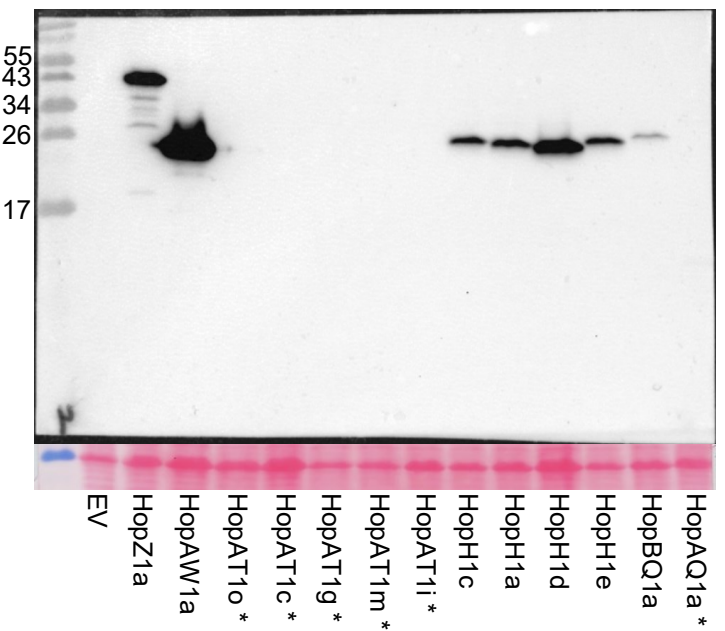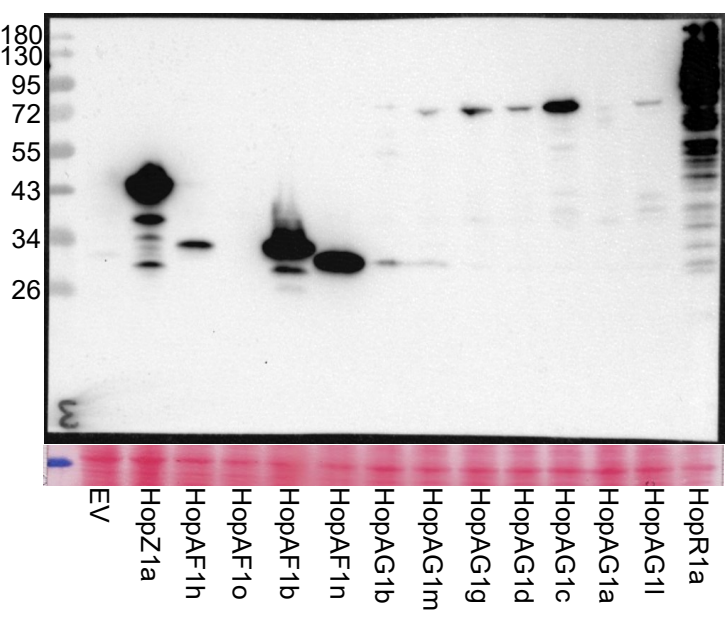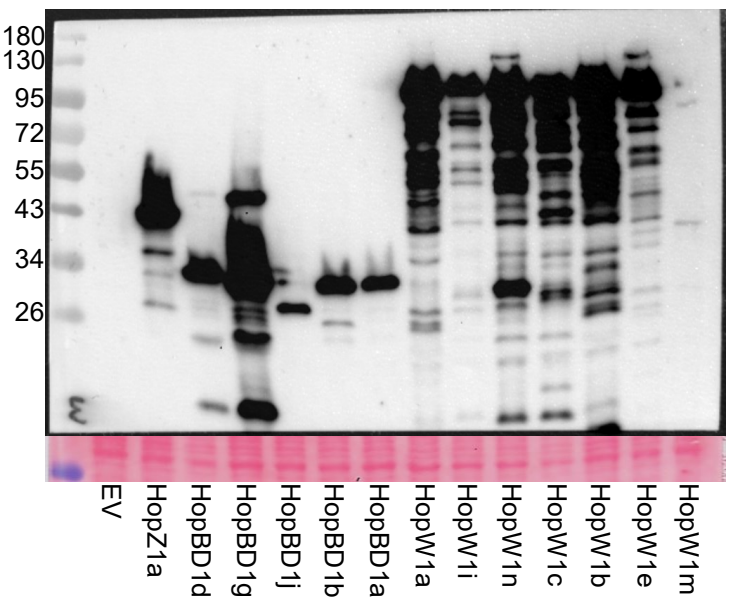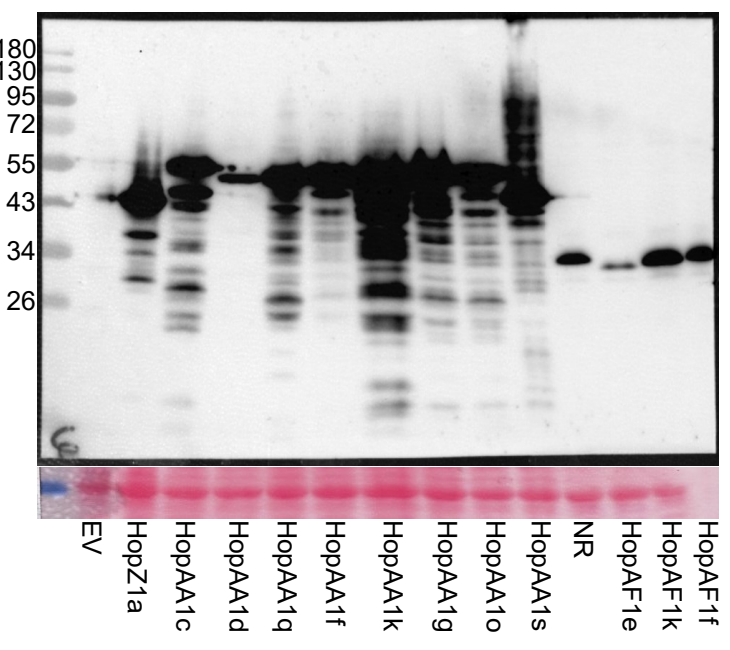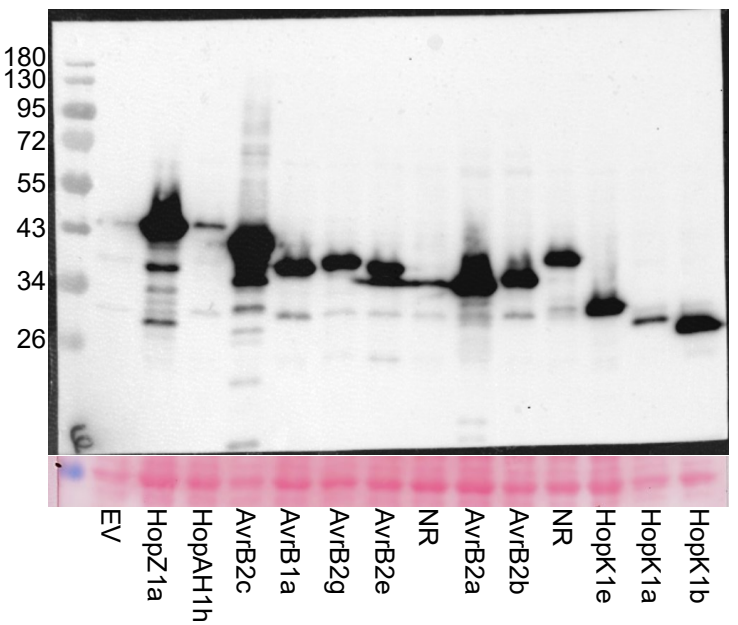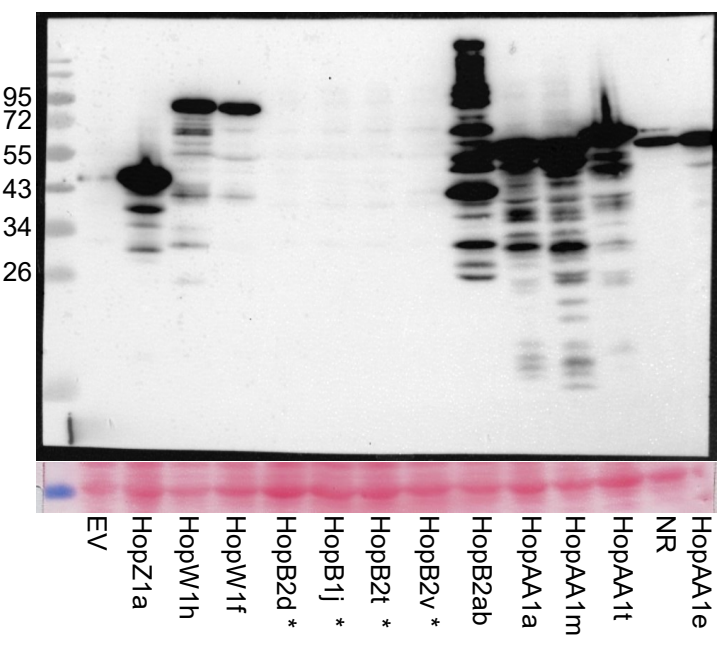

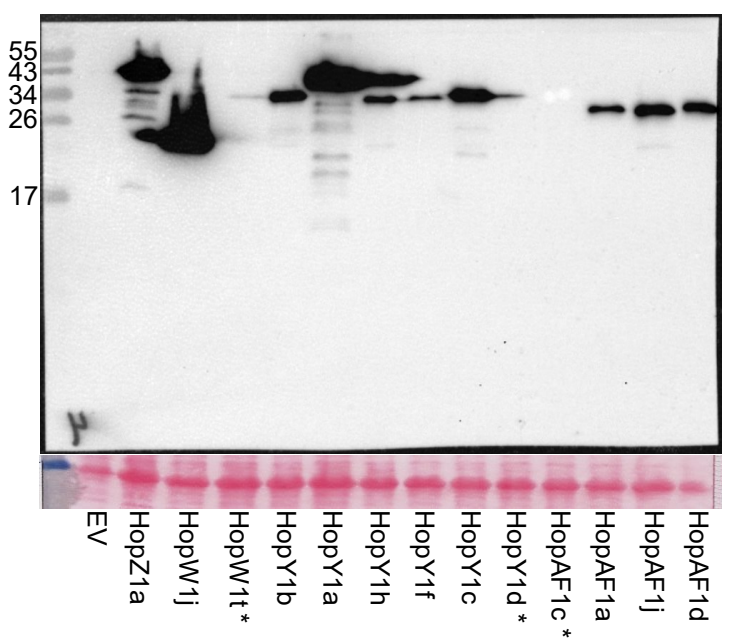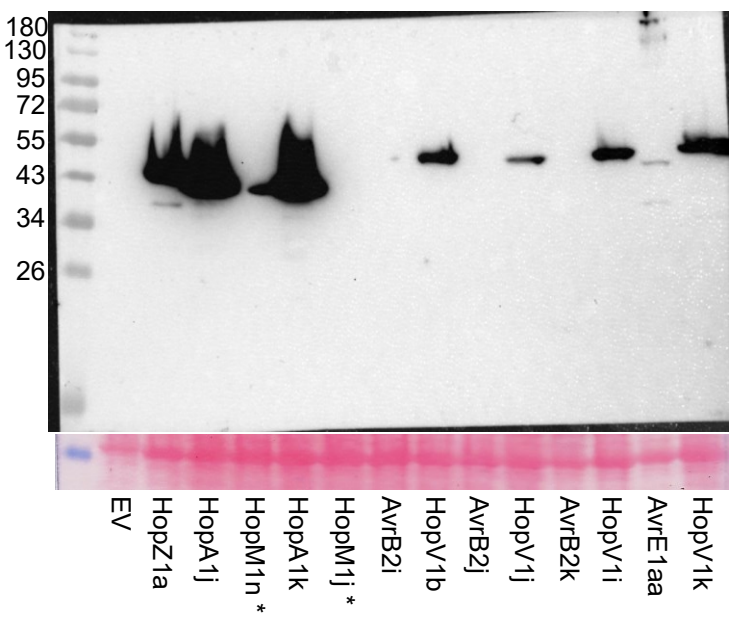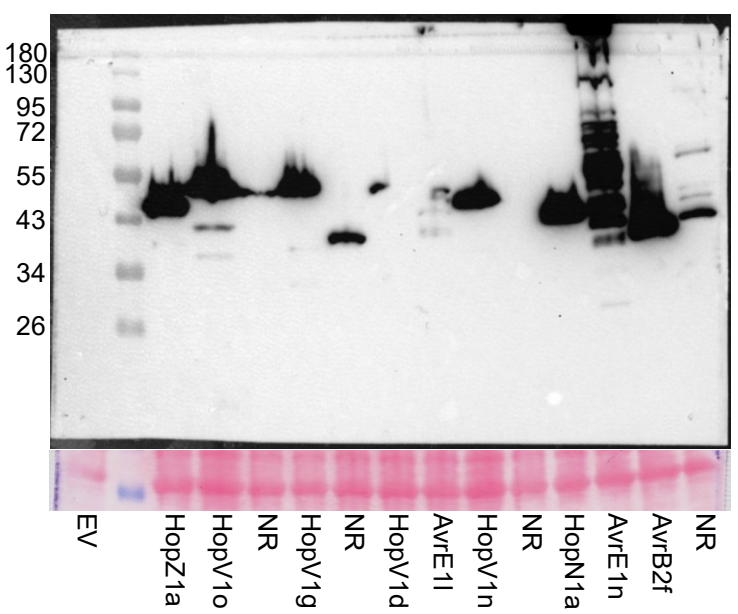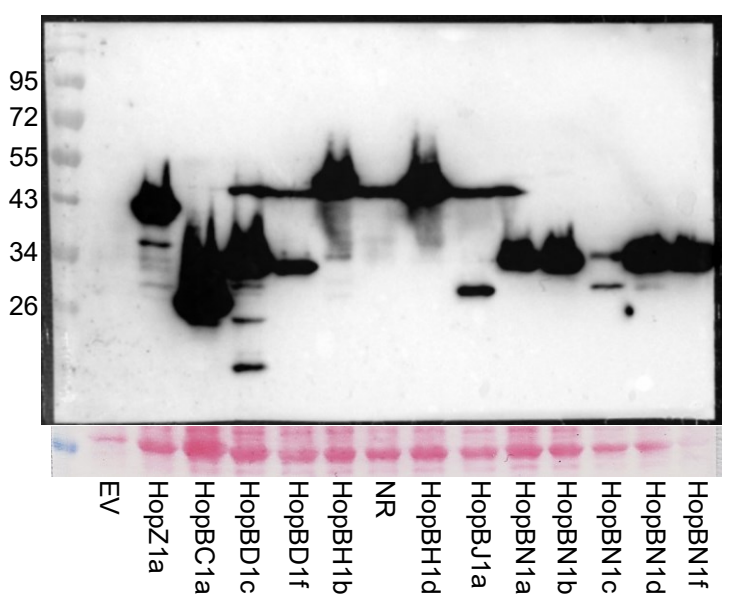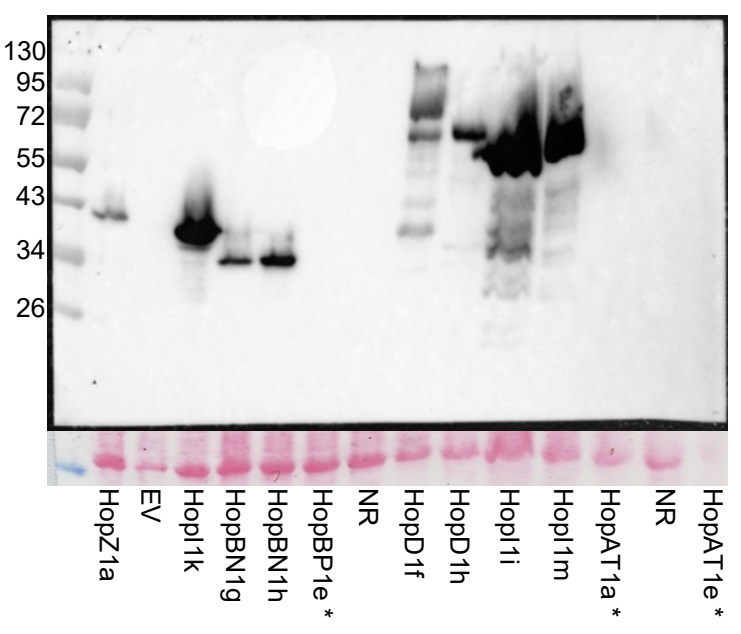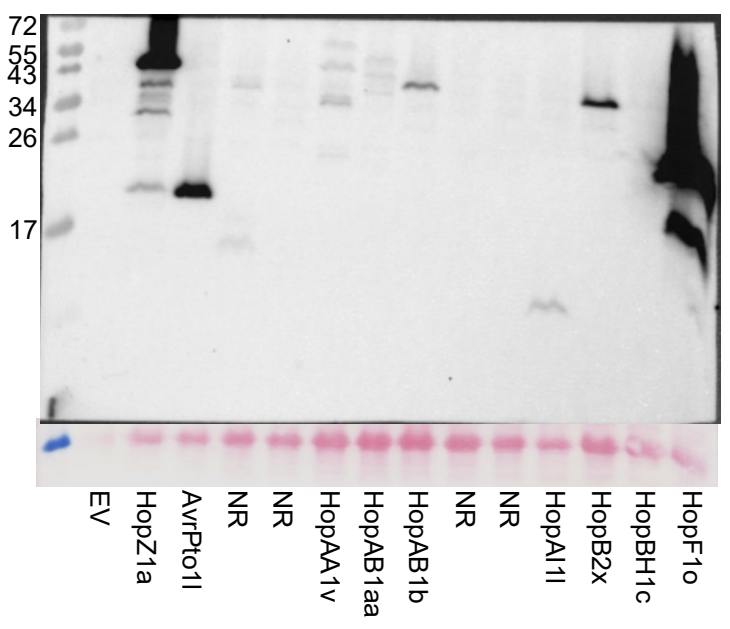

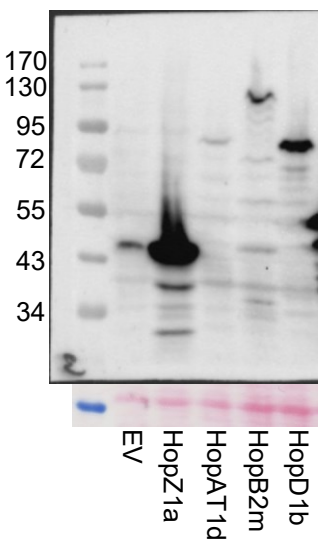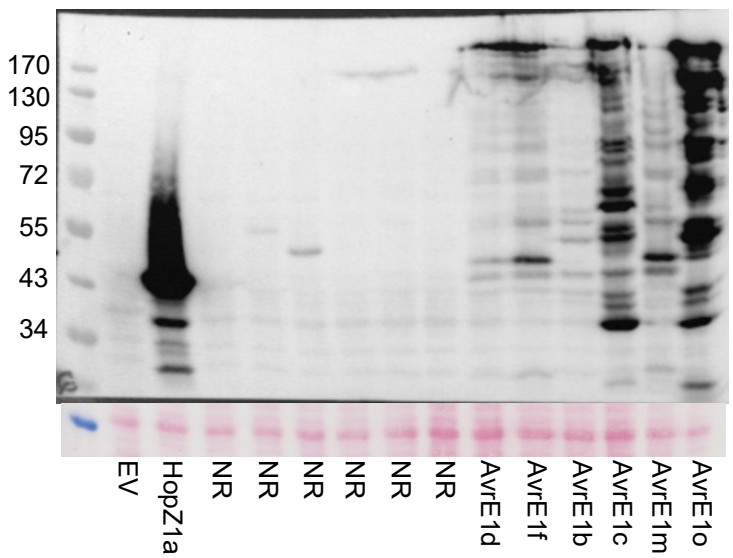

Supplement: S1 Fig — Immunoblots against the HA tag of each of the 529 PsyTEC representative alleles in PmaES4326 following overnight growth in hrp-inducing minimal media. An empty-vector (EV) and positive expression control (HopZ1a) is included in each immunoblot. A ponceau staining of the membrane is presented under each immunoblot to display equal loading. An individual asterisk (*) indicates T3SEs for which we could not detect expression. Two asterisks (**) indicates that expression was detected, but at a different size than expected. NR indicates that this sample is not relevant to this study. Numbers to the left of the immunoblots describe the molecular weight of the ladder, in kDa. (PDF) [file ppat.1010541.s005.pdf]

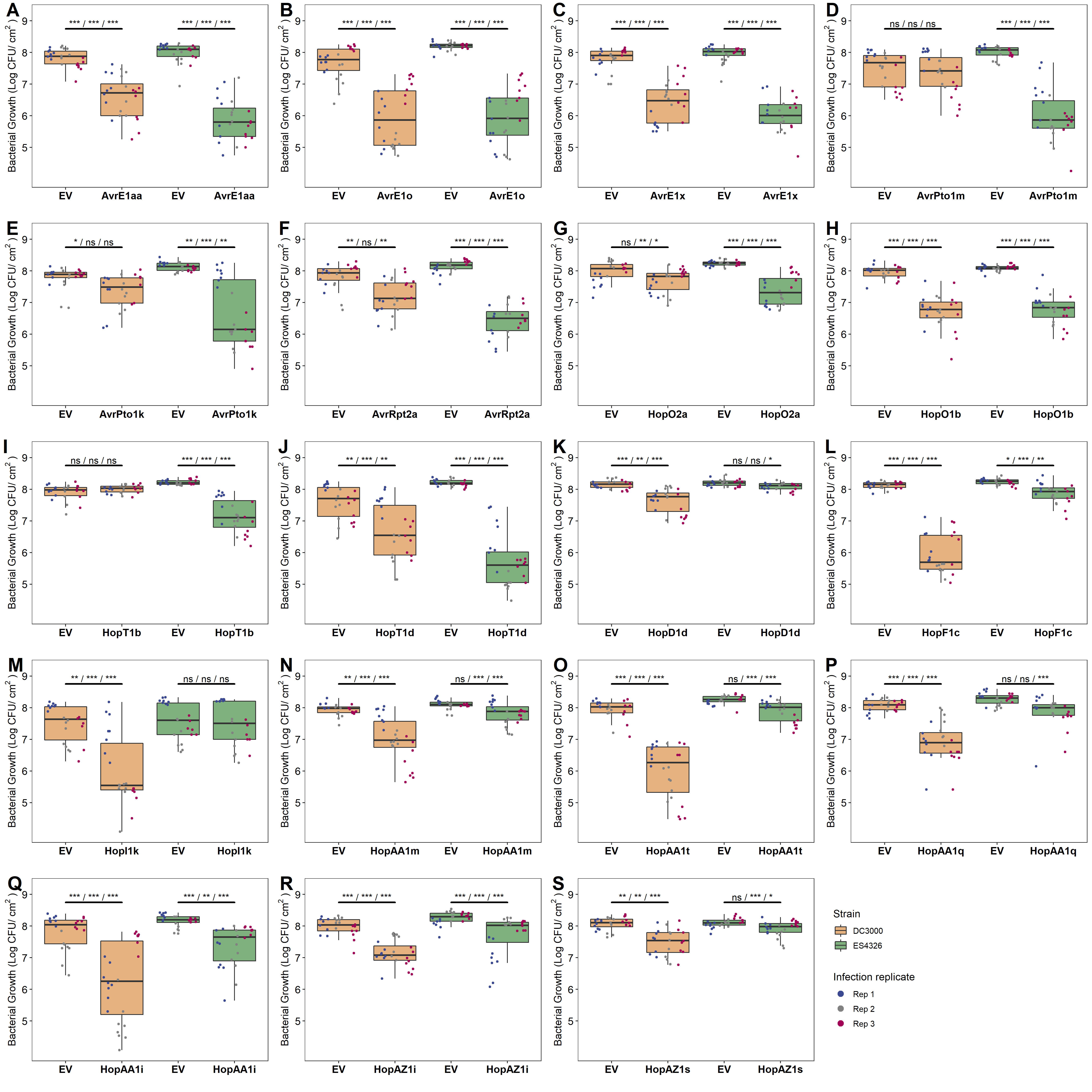

Supplement: S4 Fig — (A-J) Bacterial growth data for all 19 T3SE alleles that displayed differential ETI phenotypes when delivered from either PmaES4326 or (K-S) PtoDC3000. Asterisks indicate treatments determined to be statistically significantly different based on pairwise T-tests (* P < 0.5, ** P < 0.1, *** P < 0.01). Growth assays were performed 3 days post infection. Color-coding of the three independent infection replicates are described in the legend. (PNG) [file ppat.1010541.s008.png]

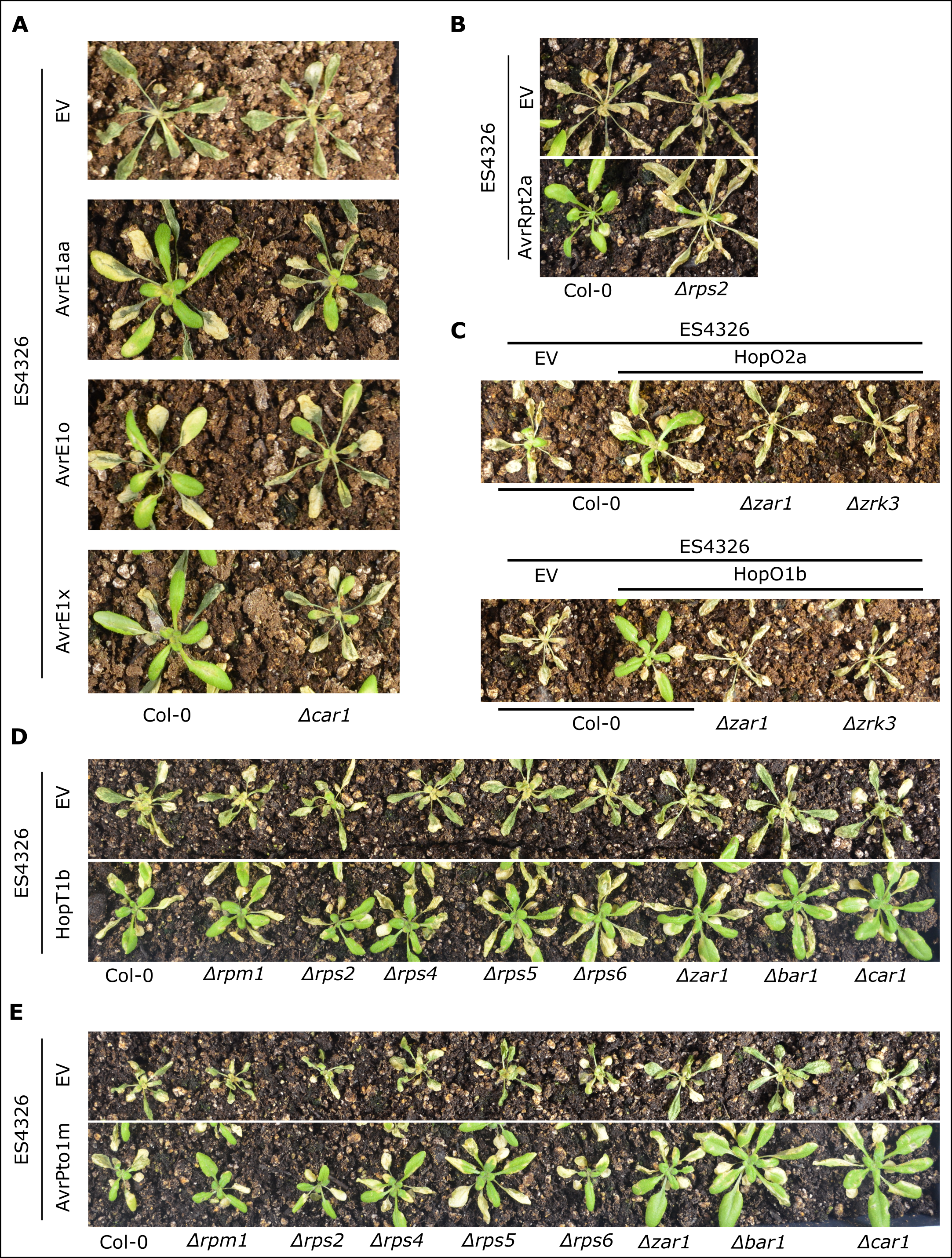

Supplement: S5 Fig — Host genetic requirements of newly identified ETI-eliciting T3SE families belonging to T3SE families with previously characterized host genetic components was confirmed, when delivered from PmaES4326. (A) AvrE1x, AvrE1o and AvrE1aa were spray-inoculated on Col-0 and Δcar1 (car1-1), (B) AvrRpt2a was spray inoculated onto Col-0 and Δrps2 and (C) HopO2a and HopO1b were spray-inoculated on Col-0, Δzar1 (zar1-1) and Δzrk3 (zrk3-1). One allele from each newly identified ETI-eliciting T3SE family, (D) AvrPto1m and (E) HopT1b, was spray inoculated onto a collection of 8 knockout lines representing each characterized NLR mediating P. syringae T3SE ETIs in A. thaliana. Experiments in panels A-C were repeated twice with similar results. Experiments in panels D and E were performed once. (PNG) [file ppat.1010541.s009.png]

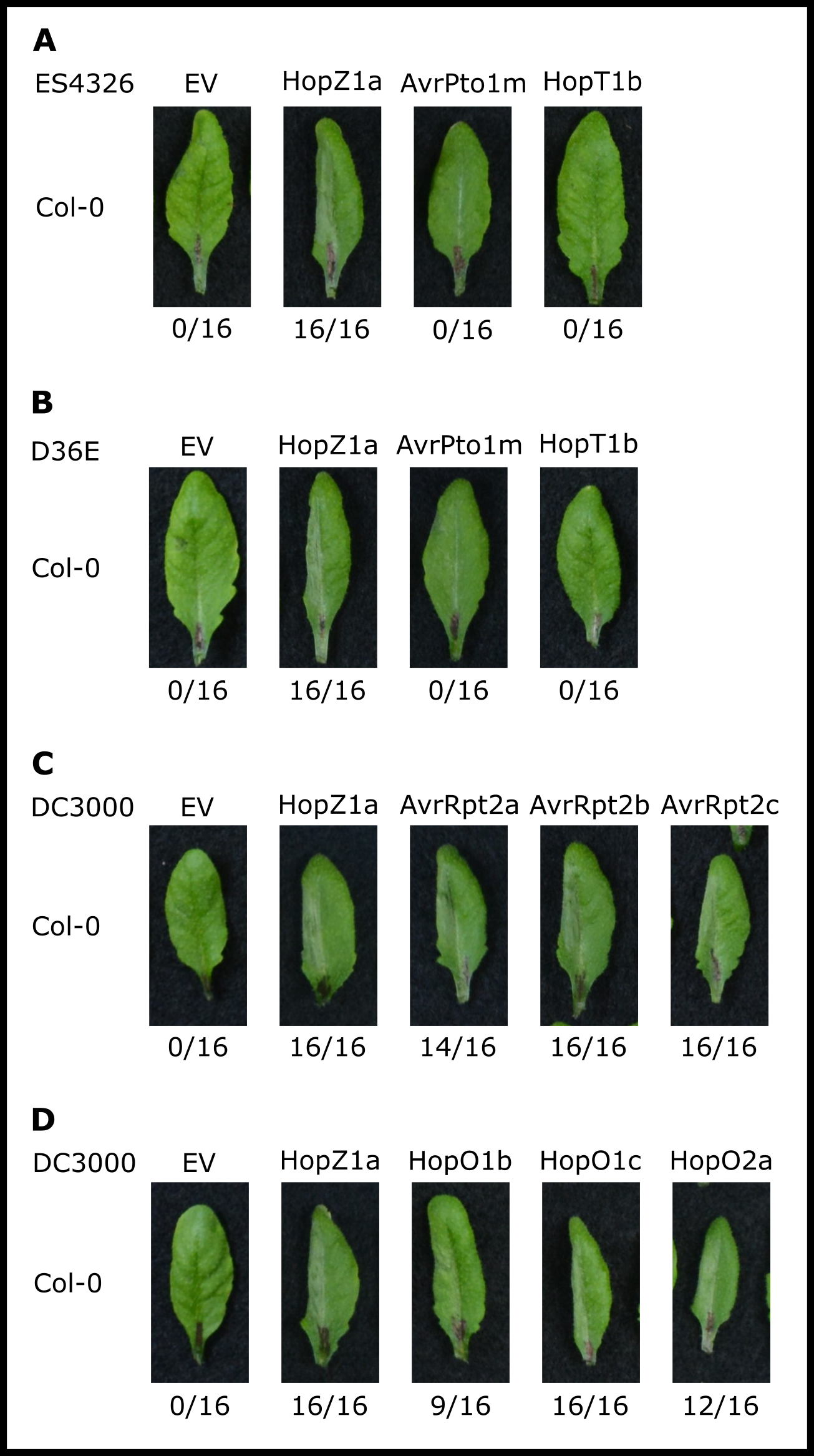

Supplement: S6 Fig — Hypersensitive response (HR) assays delivering an empty-vector (EV), HopZ1a, AvrPto1m and HopT1b from (A) PmaES4326 and from (B) the effectorless PtoDC3000 derivative D36E into the left side of A. thaliana Col-0 leaves. HR assays delivering all ETI-eliciting alleles from the (C) AvrRpt2 and the (D) HopO1/2 T3SE families, in conjunction with an empty-vector (EV) and HopZ1a control, delivered from PtoDC3000 into the left side of A. thaliana Col-0 leaves. Numbers below the representative leaf images represent the total number of observed macroscopic tissue collapses observed out of 16. These experiments were repeated 3 times with similar results. (PNG) [file ppat.1010541.s010.png]

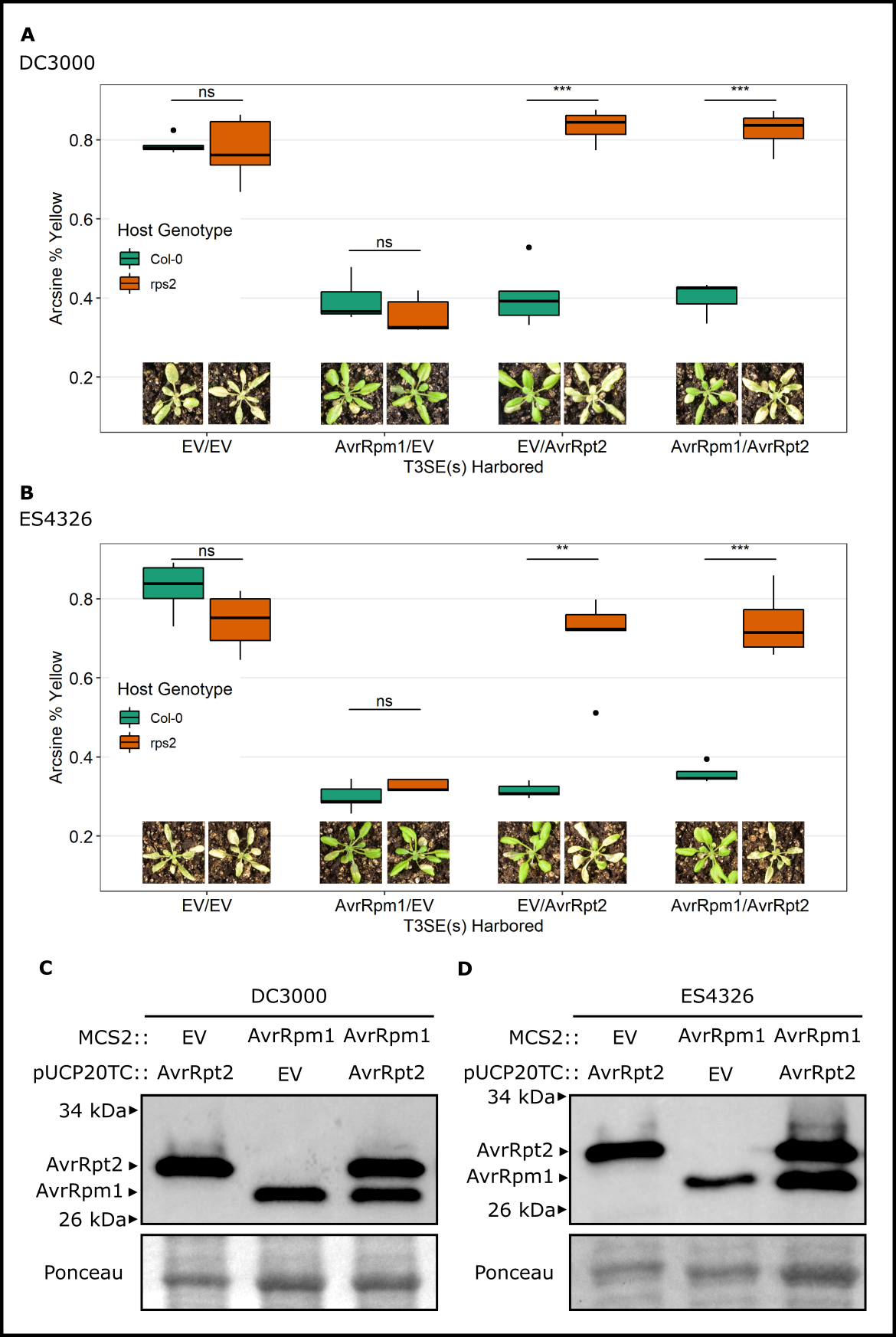

Supplement: S7 Fig — Validation that disease symptom quantification effectively captures the well characterized AvrRpm1/AvrRpt2 ETI suppression example using our two-vector system in both (A) PtoDC3000 and (B) PmaES4326. Asterisks indicate treatments determined to be statistically significantly different based on pairwise T-tests (* P < 0.5), whereas ns indicated no significant differences. Representative plant images for each treatment are included. Validation of protein accumulation through immunoblotting for all T3SEs in (C) PtoDC3000 and (D) PmaES4326 is presented, with associated Ponceau load controls below. These experiments were performed once. (PNG) [file ppat.1010541.s011.png]

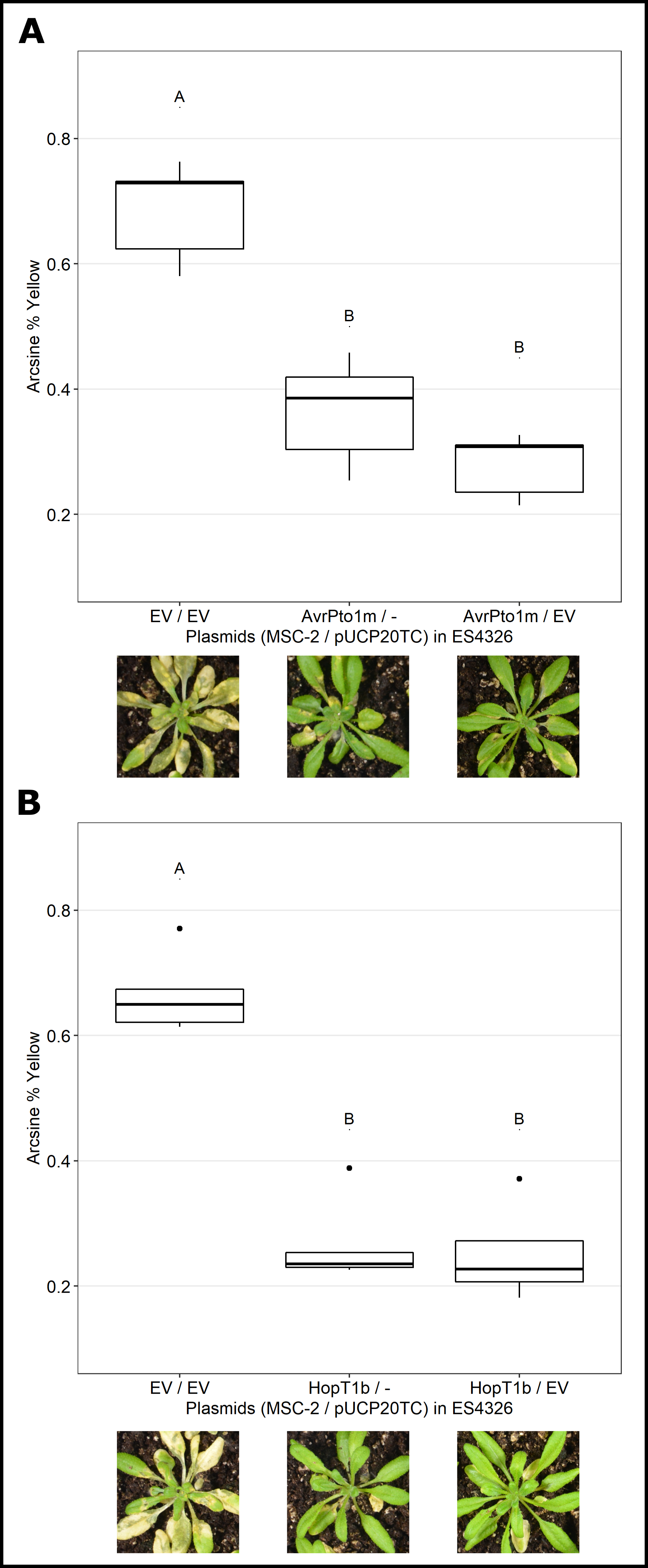

Supplement: S8 Fig — Visual disease symptom quantification through PIDIQ [38] validating similar ETI phenotypes for strains carrying only the ETI elicitor on MSC2 and those carrying an ETI elicitor on MCS2 and an empty vector pUCP20TC. Representative plant images for each treatment are included. These experiments were performed once. (PNG) [file ppat.1010541.s012.png]

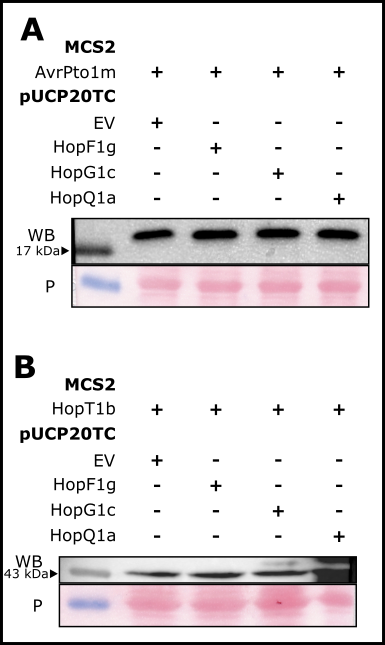

Supplement: S9 Fig — Immunoblots confirming the minimal media-induced expression of (A) AvrPto1m (expected size: 18 kDa) and (B) HopT1b (expected size: 42 kDa) in the suppression strains harboring HopF1g, HopG1c, HopQ1a or an empty vector pUCP20TC plasmid. HopQ1a (49 kDa) is of a similar size to HopT1b and can be observed in the WB panel. WB depicts the immunoblot against the ETI elicitor of interest probing for the fused HA tag; P depicts the associated Ponceau stained membrane confirming equal protein loading. The size of the molecular weight marker visible in the figure is described by the arrow and kDa value described to the left of each immunoblot. These experiments were performed once. (PNG) [file ppat.1010541.s013.png]
