# Supplementary material for: Metaeffector interactions modulate the type III effector-triggered immunity load of Pseudomonas syringae
Source: PLoS Pathog. 2022 May 16;18(5):e1010541. doi: 10.1371/journal.ppat.1010541 (PMC9135338; doi:10.1371/journal.ppat.1010541)
Supplement: S2 Fig — Images of A. thaliana Col-0 plants spray inoculated with PmaES4326 harboring representative PsyTEC T3SE alleles 5–10 days post infection that were used to determine percent chlorosis and disease score metrics presented in Fig 1. T3SE allele names are presented above the images, while the associated disease score for each treatment is described below. A positive ETI control (“P” PmaES4326 harboring HopZ1a, with a disease score of 0) and a negative ETI control (“N” PmaES4326 harboring an empty vector, with a disease score of 1) were included on each flat. T3SE descriptions are color coded based on their ETI classification using a disease score threshold of 0.45, where blue—ETI positive T3SE and red—ETI negative T3SE. Shaded-out plant columns are not relevant to this study. (PDF) [file ppat.1010541.s006.pdf]

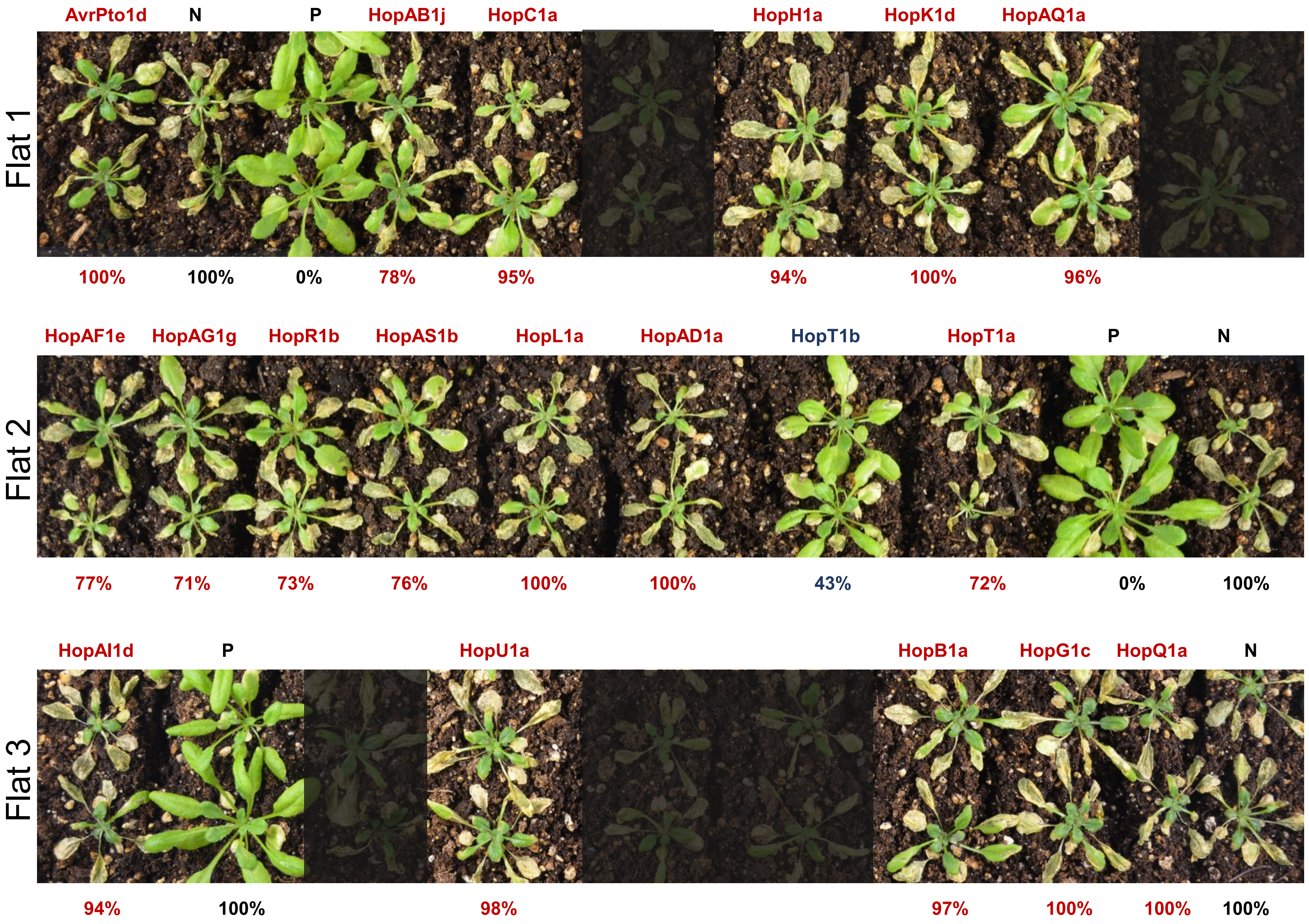

Flat 1
AvrPto1d
N
P
HopAB1j
HopC1a
HopH1a
HopK1d
HopAQ1a
100%
100%
0%
78%
95%
94%
100%
96%
Flat 2
HopAF1e
HopAG1g
HopR1b
HopAS1b
HopL1a
HopAD1a
HopT1b
HopT1a
P
N
77%
71%
73%
76%
100%
100%
43%
72%
0%
100%
Flat 3
HopAI1d
P
HopU1a
HopB1a
HopG1c
HopQ1a
N
94%
100%
98%
97%
100%
100%
100%

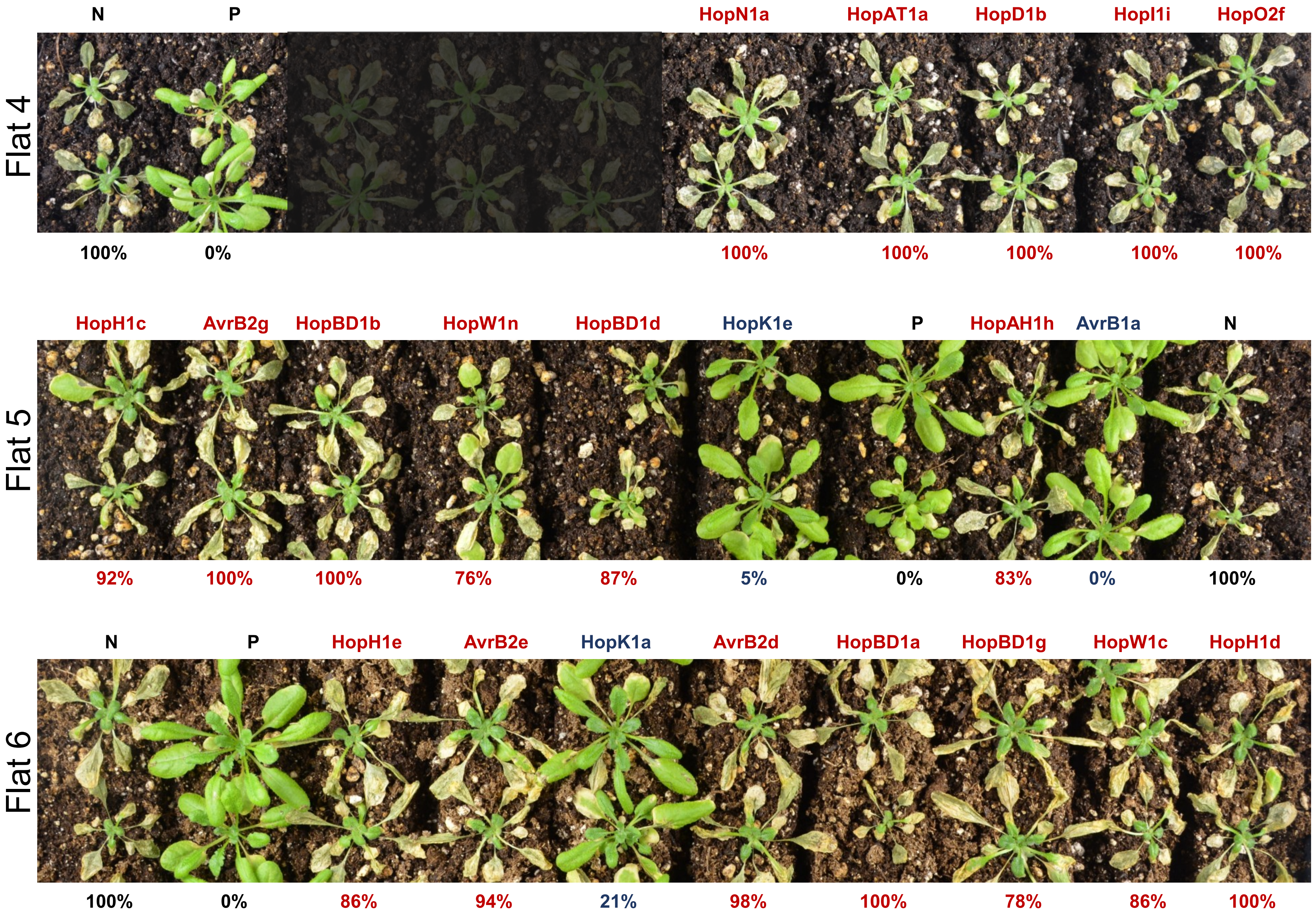
Flat 4
N
P
HopN1a
HopAT1a
HopD1b
HopI1i
HopO2f
100%
0%
100%
100%
100%
100%
100%
Flat 5
HopH1c
AvrB2g
HopBD1b
HopW1n
HopBD1d
HopK1e
P
HopAH1h
AvrB1a
N
92%
100%
100%
76%
87%
5%
0%
83%
0%
100%
Flat 6
N
P
HopH1e
AvrB2e
HopK1a
AvrB2d
HopBD1a
HopBD1g
HopW1c
HopH1d
100%
0%
86%
94%
21%
98%
100%
78%
86%
100%

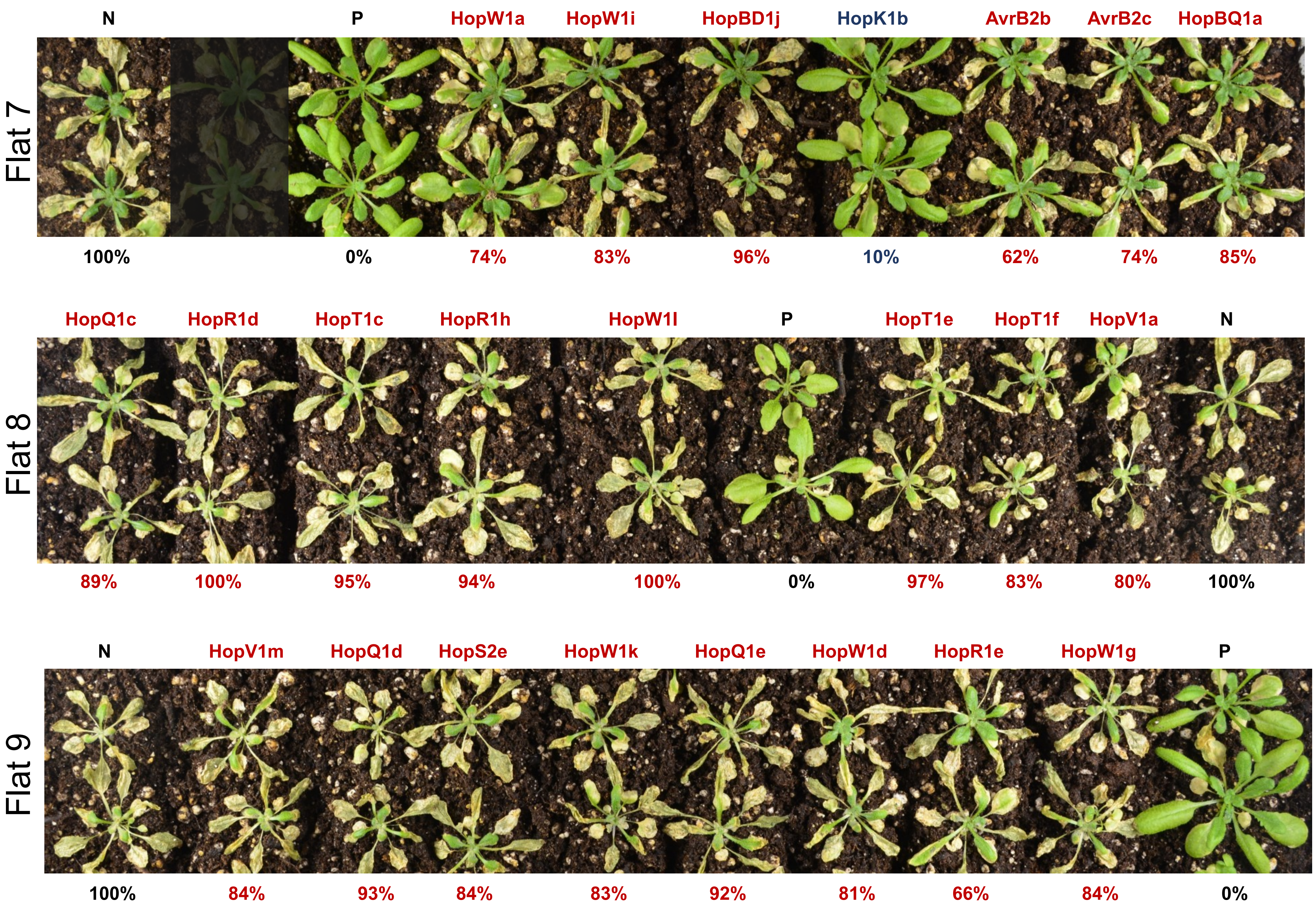
Flat 7
N
P
HopW1a
HopW1i
HopBD1j
HopK1b
AvrB2b
AvrB2c
HopBQ1a
100%
0%
74%
83%
96%
10%
62%
74%
85%
Flat 8
HopQ1c
HopR1d
HopT1c
HopR1h
HopW1l
P
HopT1e
HopT1f
HopV1a
N
89%
100%
95%
94%
100%
0%
97%
83%
80%
100%
Flat 9
N
HopV1m
HopQ1d
HopS2e
HopW1k
HopQ1e
HopW1d
HopR1e
HopW1g
P
100%
84%
93%
84%
83%
92%
81%
66%
84%
0%

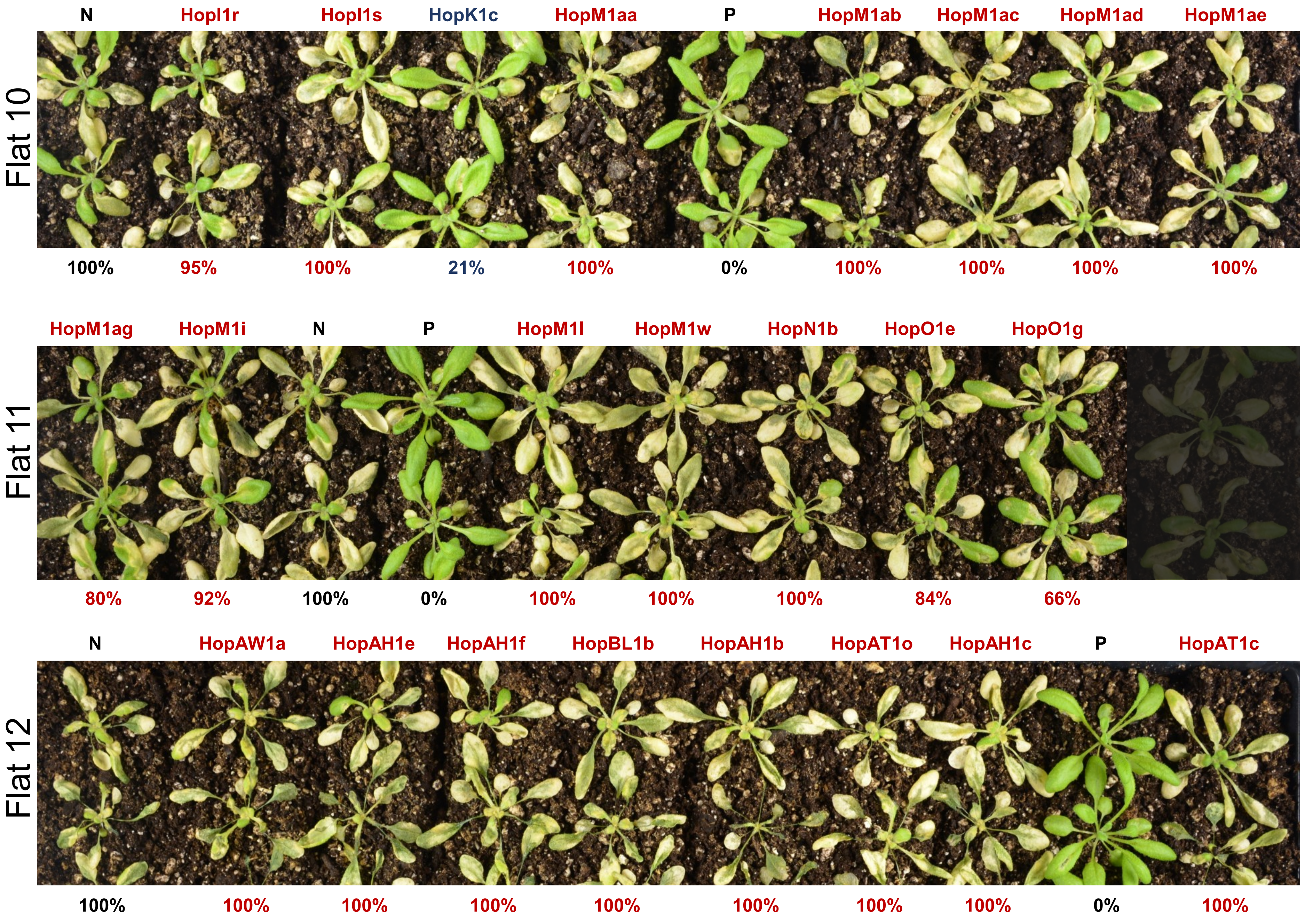
Flat 10
N
HopI1r
HopI1s
HopK1c
HopM1aa
P
HopM1ab
HopM1ac
HopM1ad
HopM1ae
100%
95%
100%
21%
100%
0%
100%
100%
100%
100%
Flat 11
HopM1ag
HopM1i
N
P
HopM1l
HopM1w
HopN1b
HopO1e
HopO1g
80%
92%
100%
0%
100%
100%
100%
84%
66%
Flat 12
N
HopAW1a
HopAH1e
HopAH1f
HopBL1b
HopAH1b
HopAT1o
HopAH1c
P
HopAT1c
100%
100%
100%
100%
100%
100%
100%
100%
0%
100%

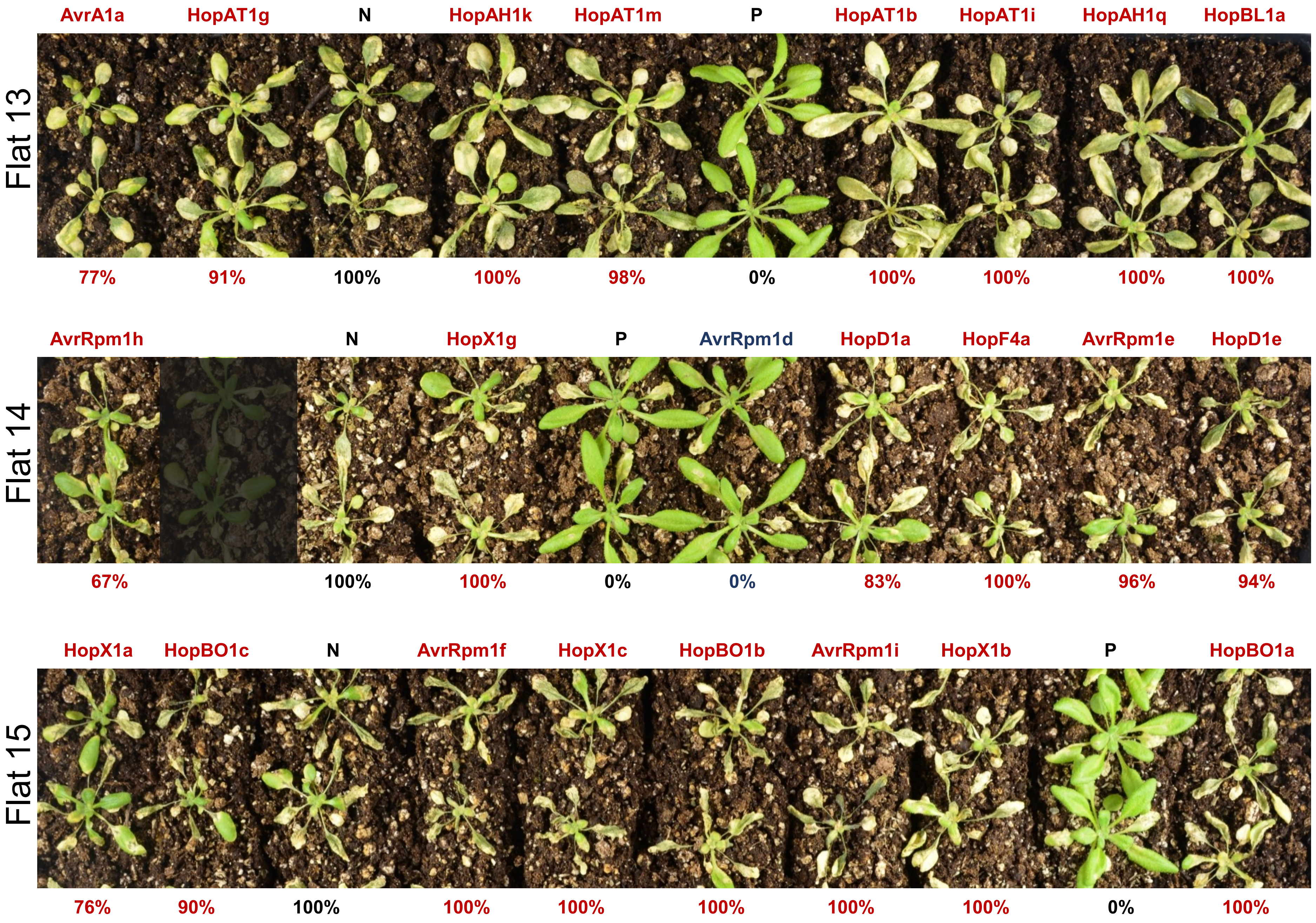
Flat 13
AvrA1a
HopAT1g
N
HopAH1k
HopAT1m
P
HopAT1b
HopAT1i
HopAH1q
HopBL1a
77%
91%
100%
100%
98%
0%
100%
100%
100%
100%
Flat 14
AvrRpm1h
N
HopX1g
P
AvrRpm1d
HopD1a
HopF4a
AvrRpm1e
HopD1e
67%
100%
100%
0%
0%
83%
100%
96%
94%
Flat 15
HopX1a
HopBO1c
N
AvrRpm1f
HopX1c
HopBO1b
AvrRpm1i
HopX1b
P
HopBO1a
76%
90%
100%
100%
100%
100%
100%
100%
0%
100%

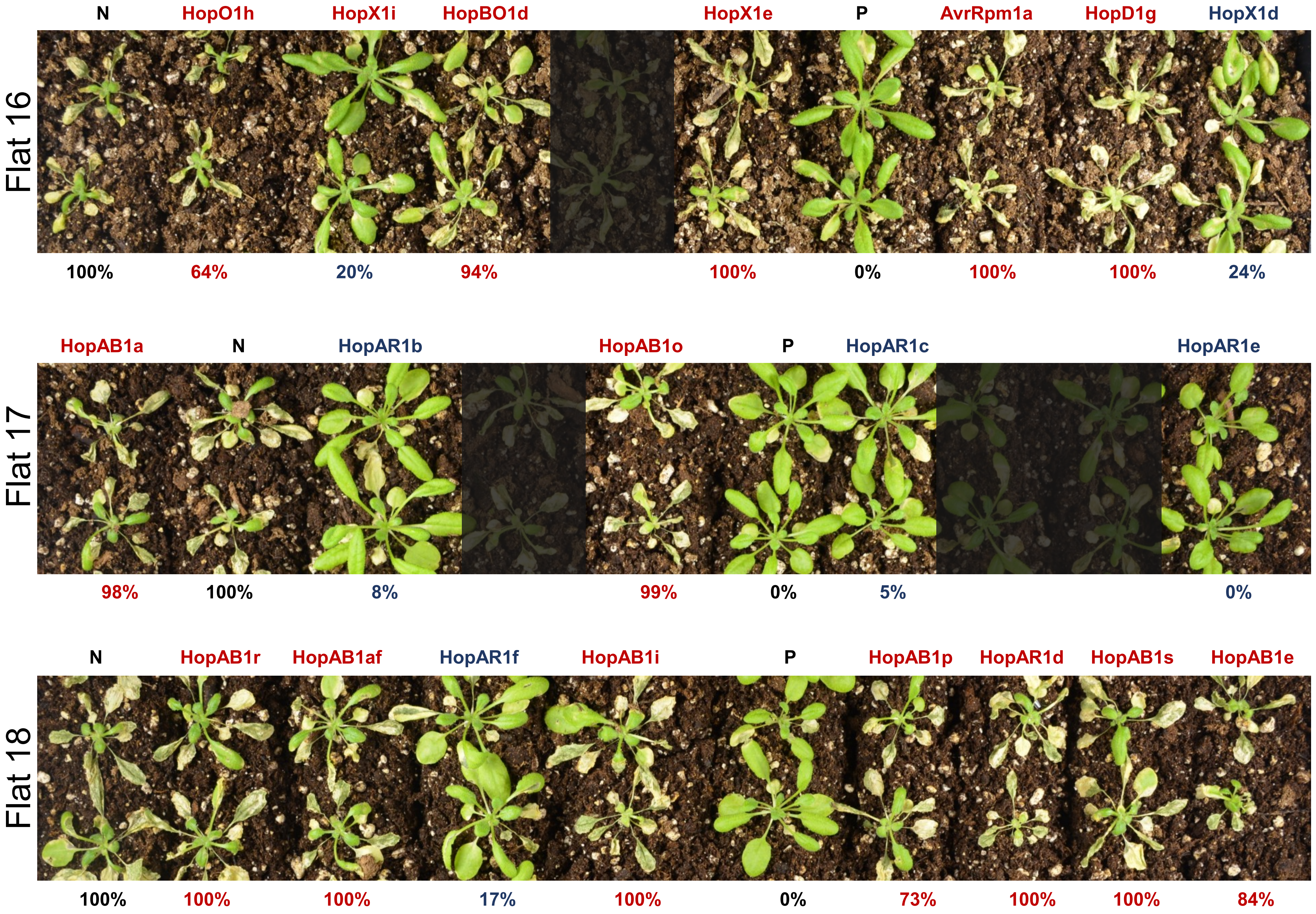
Flat 16
N
HopO1h
HopX1i
HopBO1d
HopX1e
P
AvrRpm1a
HopD1g
HopX1d
100%
64%
20%
94%
100%
0%
100%
100%
24%
Flat 17
HopAB1a
N
HopAR1b
HopAB1o
P
HopAR1c
HopAR1e
98%
100%
8%
99%
0%
5%
0%
Flat 18
N
HopAB1r
HopAB1af
HopAR1f
HopAB1i
P
HopAB1p
HopAR1d
HopAB1s
HopAB1e
100%
100%
100%
17%
100%
0%
73%
100%
100%
84%

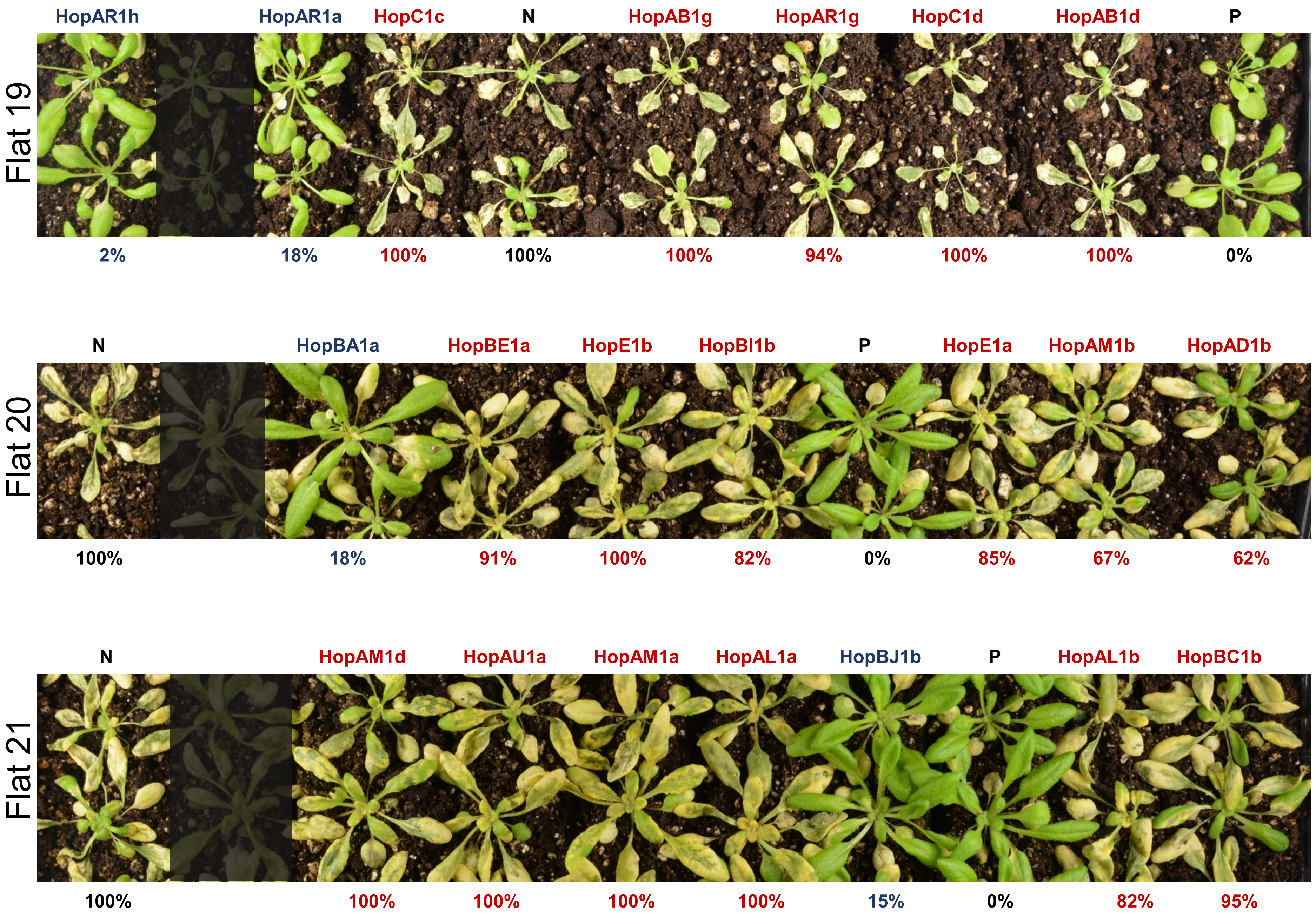

Flat 19
HopAR1h
HopAR1a
HopC1c
N
HopAB1g
HopAR1g
HopC1d
HopAB1d
P
2%
18%
100%
100%
100%
94%
100%
100%
0%
Flat 20
N
HopBA1a
HopBE1a
HopE1b
HopBI1b
P
HopE1a
HopAM1b
HopAD1b
100%
18%
91%
100%
82%
0%
85%
67%
62%
Flat 21
N
HopAM1d
HopAU1a
HopAM1a
HopAL1a
HopBJ1b
P
HopAL1b
HopBC1b
100%
100%
100%
100%
100%
15%
0%
82%
95%

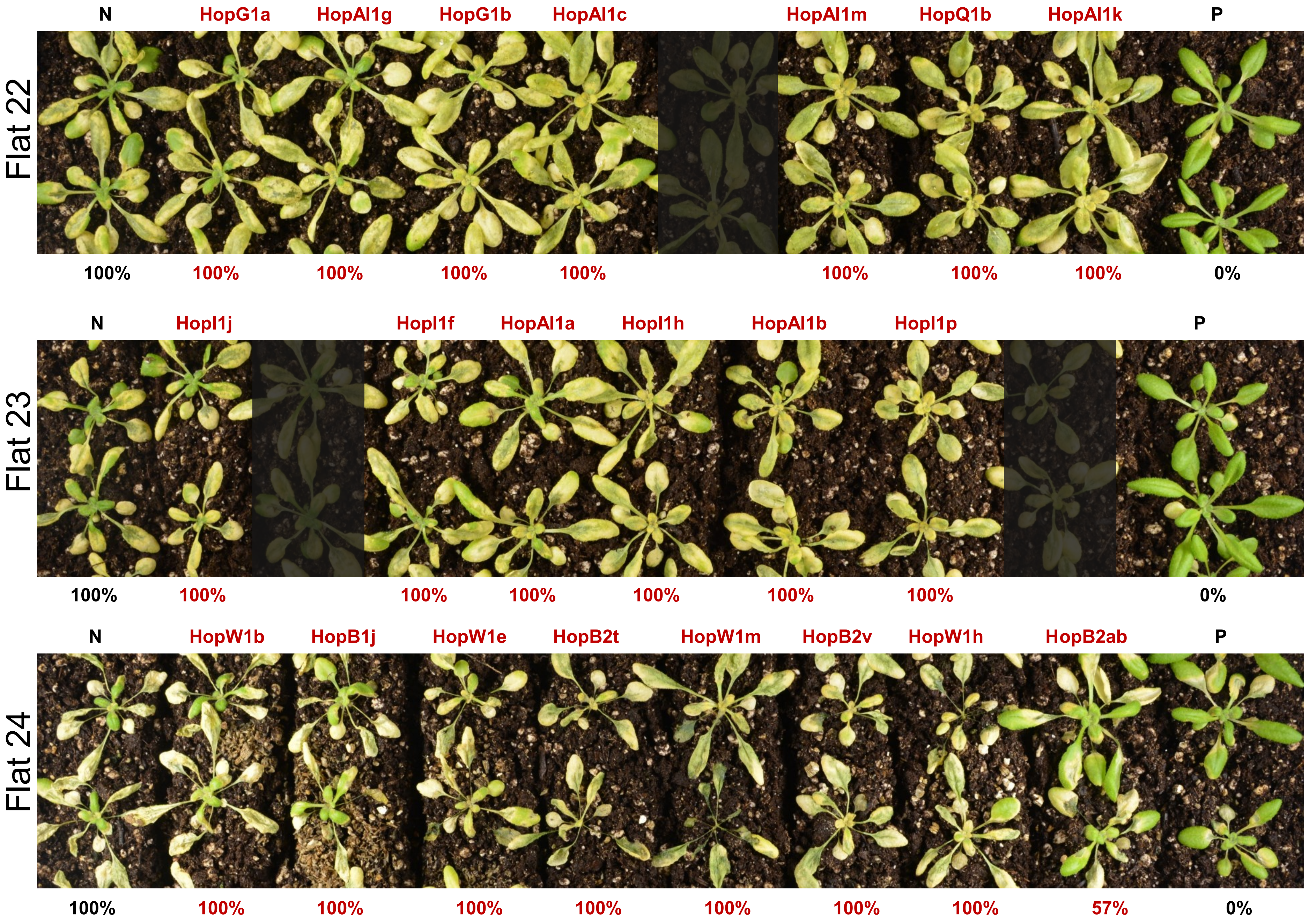
Flat 22
N
HopG1a
HopAI1g
HopG1b
HopAI1c
HopAI1m
HopQ1b
HopAI1k
P
100%
100%
100%
100%
100%
100%
100%
100%
0%
Flat 23
N
HopI1j
HopI1f
HopAI1a
HopI1h
HopAI1b
HopI1p
P
100%
100%
100%
100%
100%
100%
100%
0%
Flat 24
N
HopW1b
HopB1j
HopW1e
HopB2t
HopW1m
HopB2v
HopW1h
HopB2ab
P
100%
100%
100%
100%
100%
100%
100%
100%
57%
0%

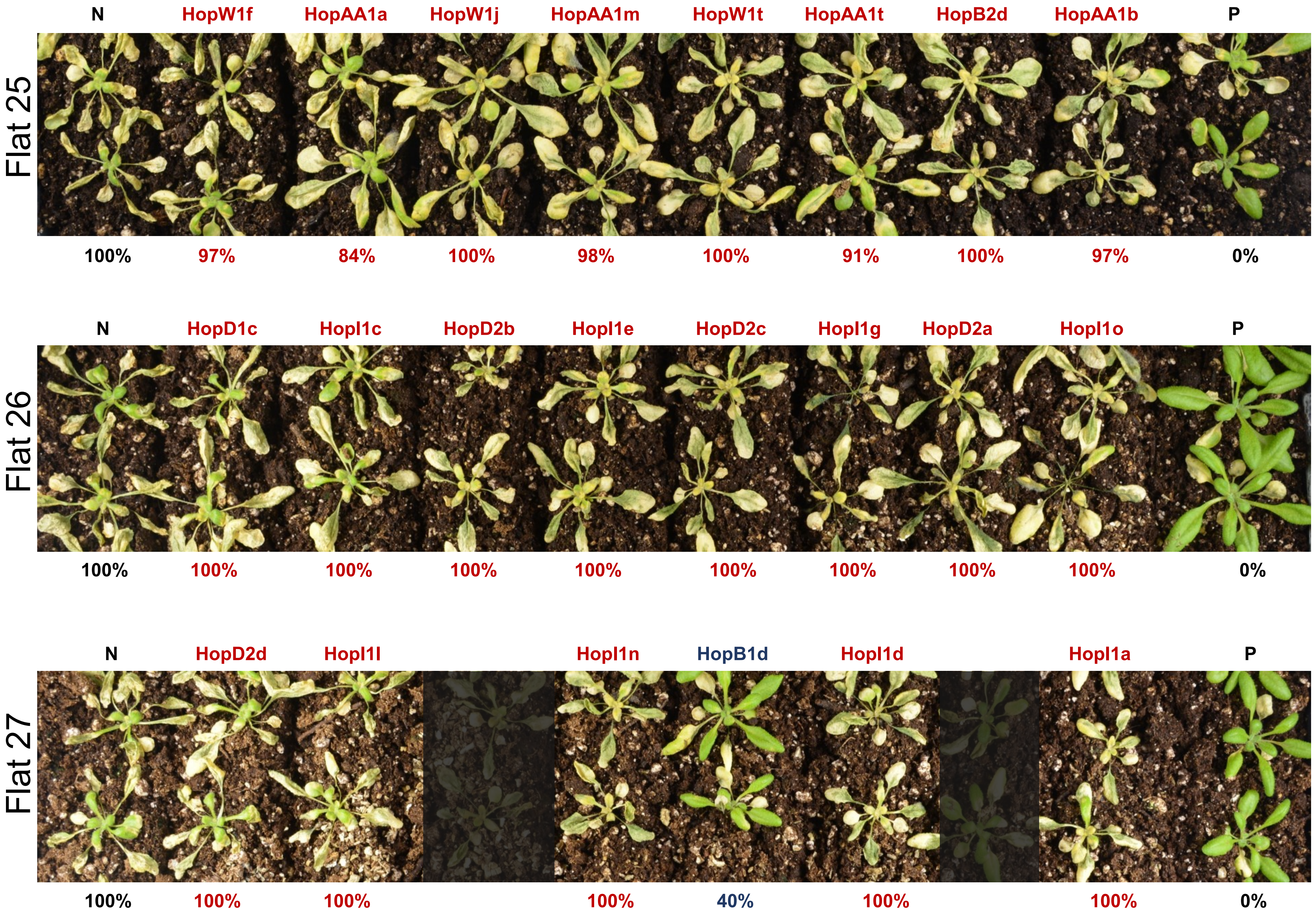

Flat 25
N
HopW1f
HopAA1a
HopW1j
HopAA1m
HopW1t
HopAA1t
HopB2d
HopAA1b
P
100%
97%
84%
100%
98%
100%
91%
100%
97%
0%
Flat 26
N
HopD1c
HopI1c
HopD2b
HopI1e
HopD2c
HopI1g
HopD2a
HopI1o
P
100%
100%
100%
100%
100%
100%
100%
100%
100%
0%
Flat 27
N
HopD2d
HopI1l
HopI1n
HopB1d
HopI1d
HopI1a
P
100%
100%
100%
100%
40%
100%
100%
0%

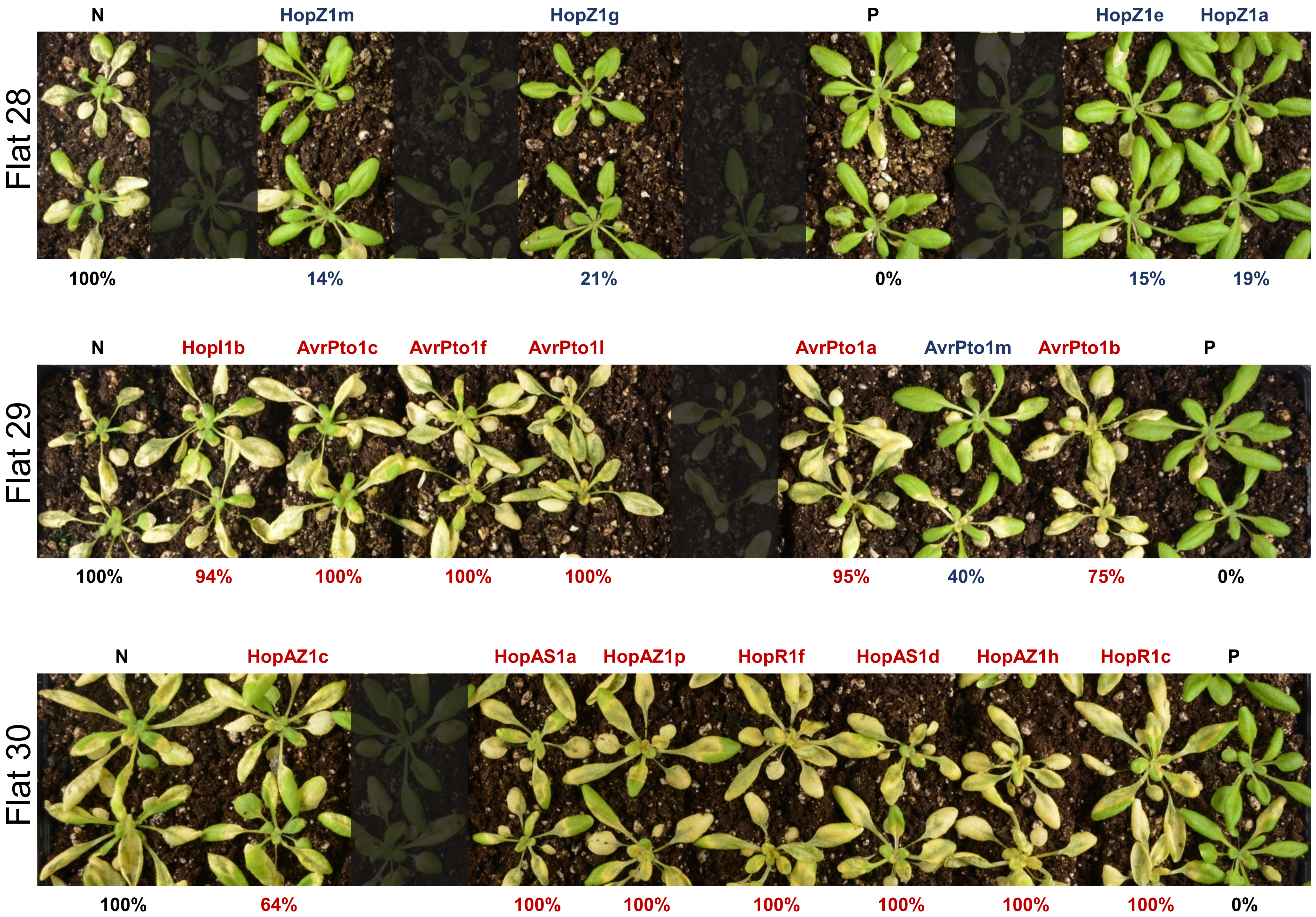
Flat 28
N
HopZ1m
HopZ1g
P
HopZ1e
HopZ1a
100%
14%
21%
0%
15%
19%
Flat 29
N
HopI1b
AvrPto1c
AvrPto1f
AvrPto1l
AvrPto1a
AvrPto1m
AvrPto1b
P
100%
94%
100%
100%
100%
95%
40%
75%
0%
Flat 30
N
HopAZ1c
HopAS1a
HopAZ1p
HopR1f
HopAS1d
HopAZ1h
HopR1c
P
100%
64%
100%
100%
100%
100%
100%
100%
0%

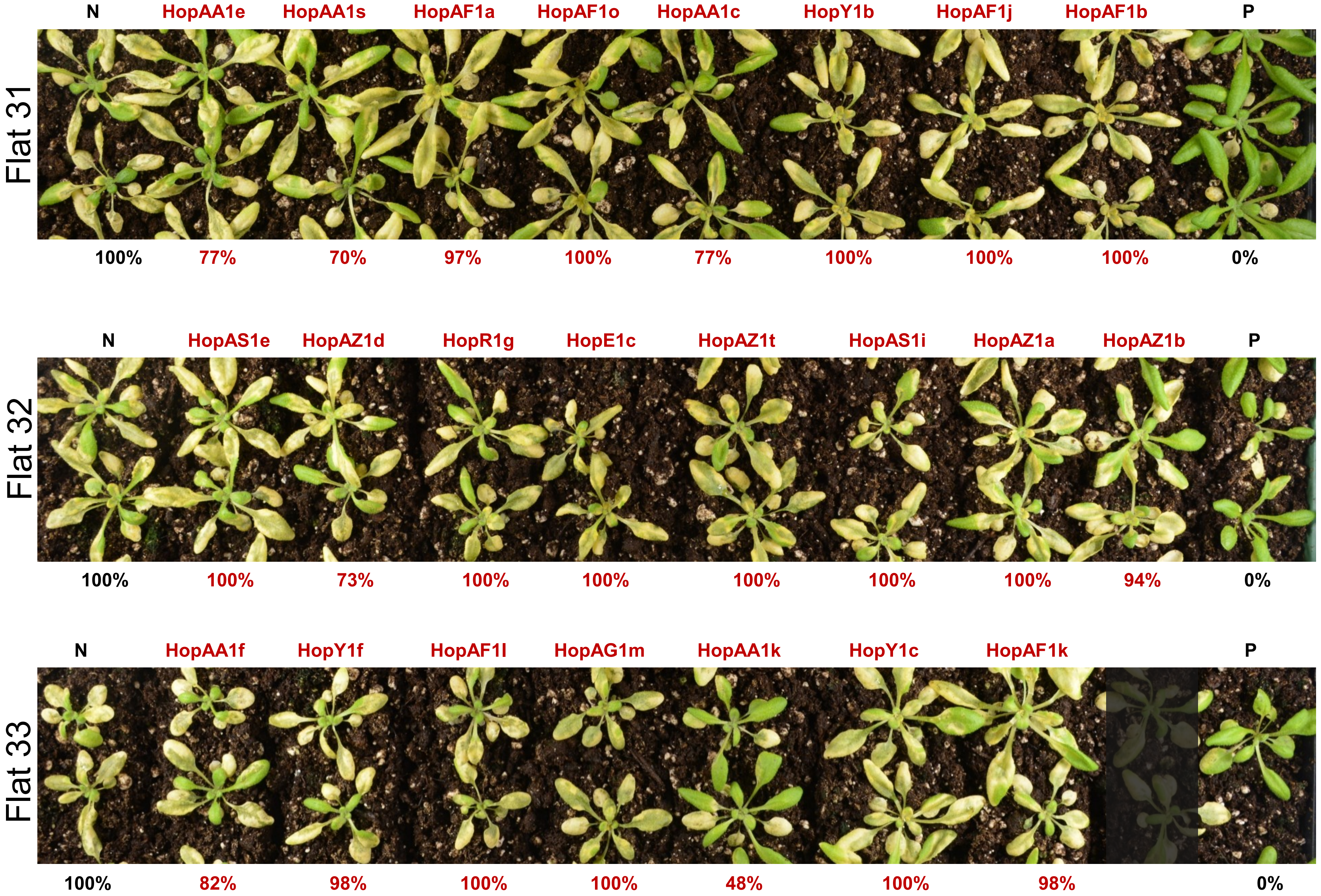
Flat 31
N
HopAA1e
HopAA1s
HopAF1a
HopAF1o
HopAA1c
HopY1b
HopAF1j
HopAF1b
P
100%
77%
70%
97%
100%
77%
100%
100%
100%
0%
Flat 32
N
HopAS1e
HopAZ1d
HopR1g
HopE1c
HopAZ1t
HopAS1i
HopAZ1a
HopAZ1b
P
100%
100%
73%
100%
100%
100%
100%
100%
94%
0%
Flat 33
N
HopAA1f
HopY1f
HopAF1l
HopAG1m
HopAA1k
HopY1c
HopAF1k
P
100%
82%
98%
100%
100%
48%
100%
98%
0%

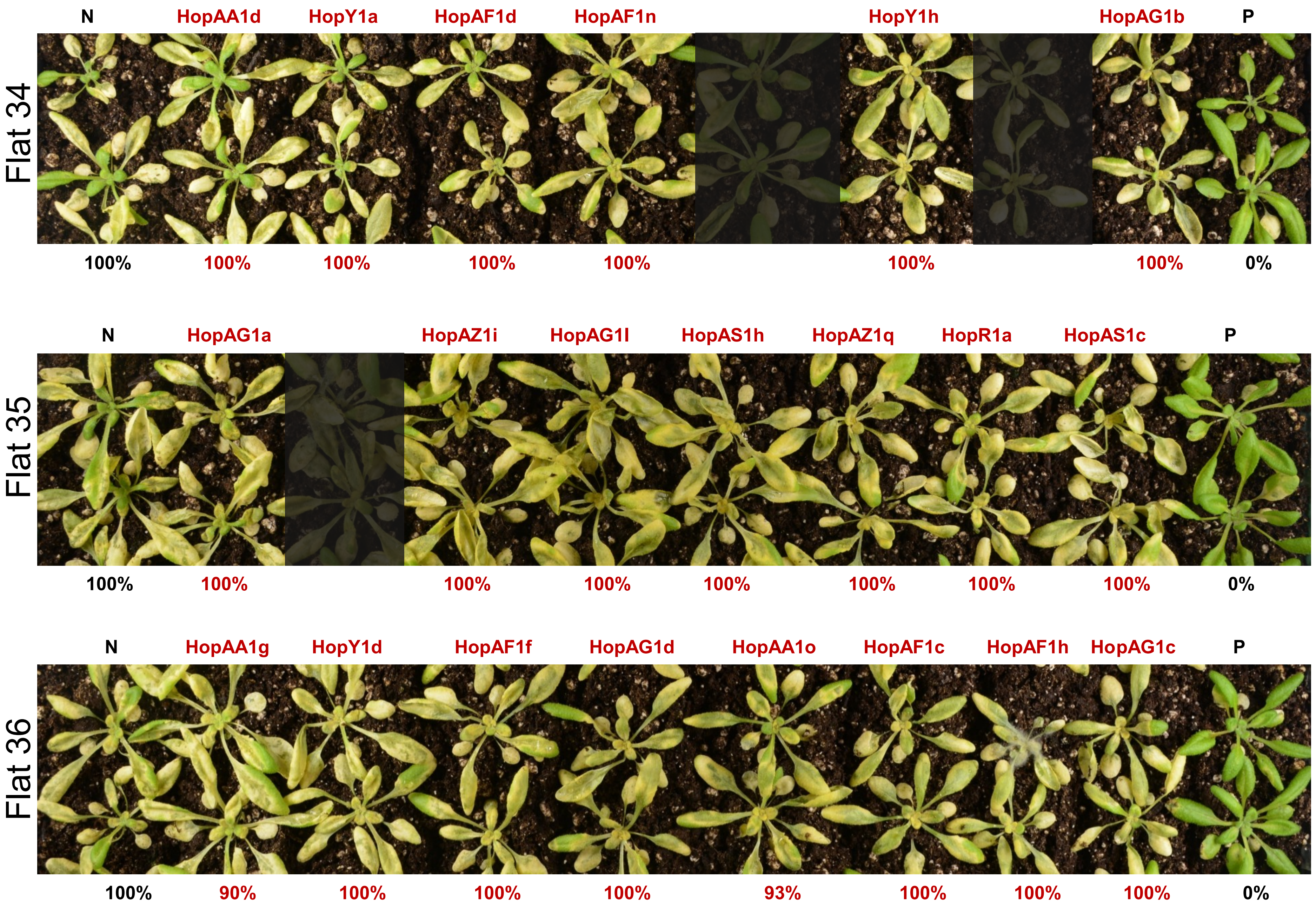
Flat 34
N
HopAA1d
HopY1a
HopAF1d
HopAF1n
HopY1h
HopAG1b
P
100%
100%
100%
100%
100%
100%
100%
0%
Flat 35
N
HopAG1a
HopAZ1i
HopAG1l
HopAS1h
HopAZ1q
HopR1a
HopAS1c
P
100%
100%
100%
100%
100%
100%
100%
100%
0%
Flat 36
N
HopAA1g
HopY1d
HopAF1f
HopAG1d
HopAA1o
HopAF1c
HopAF1h
HopAG1c
P
100%
90%
100%
100%
100%
93%
100%
100%
100%
0%

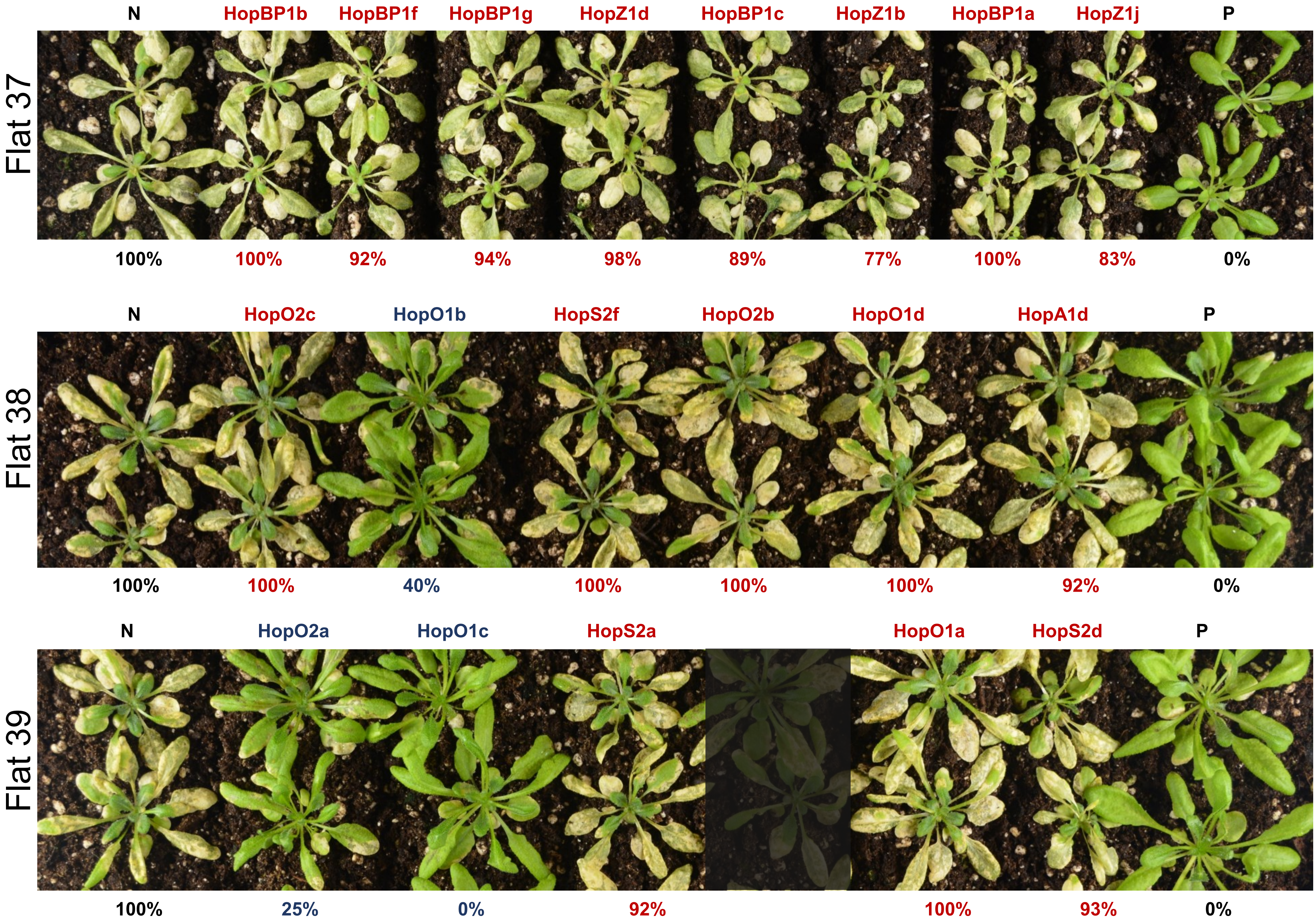
N
HopBP1b
HopBP1f
HopBP1g
HopZ1d
HopBP1c
HopZ1b
HopBP1a
HopZ1j
P
Flat 37
100%
100%
92%
94%
98%
89%
77%
100%
83%
0%
N
HopO2c
HopO1b
HopS2f
HopO2b
HopO1d
HopA1d
P
Flat 38
100%
100%
40%
100%
100%
100%
92%
0%
N
HopO2a
HopO1c
HopS2a
HopO1a
HopS2d
P
Flat 39
100%
25%
0%
92%
100%
93%
0%

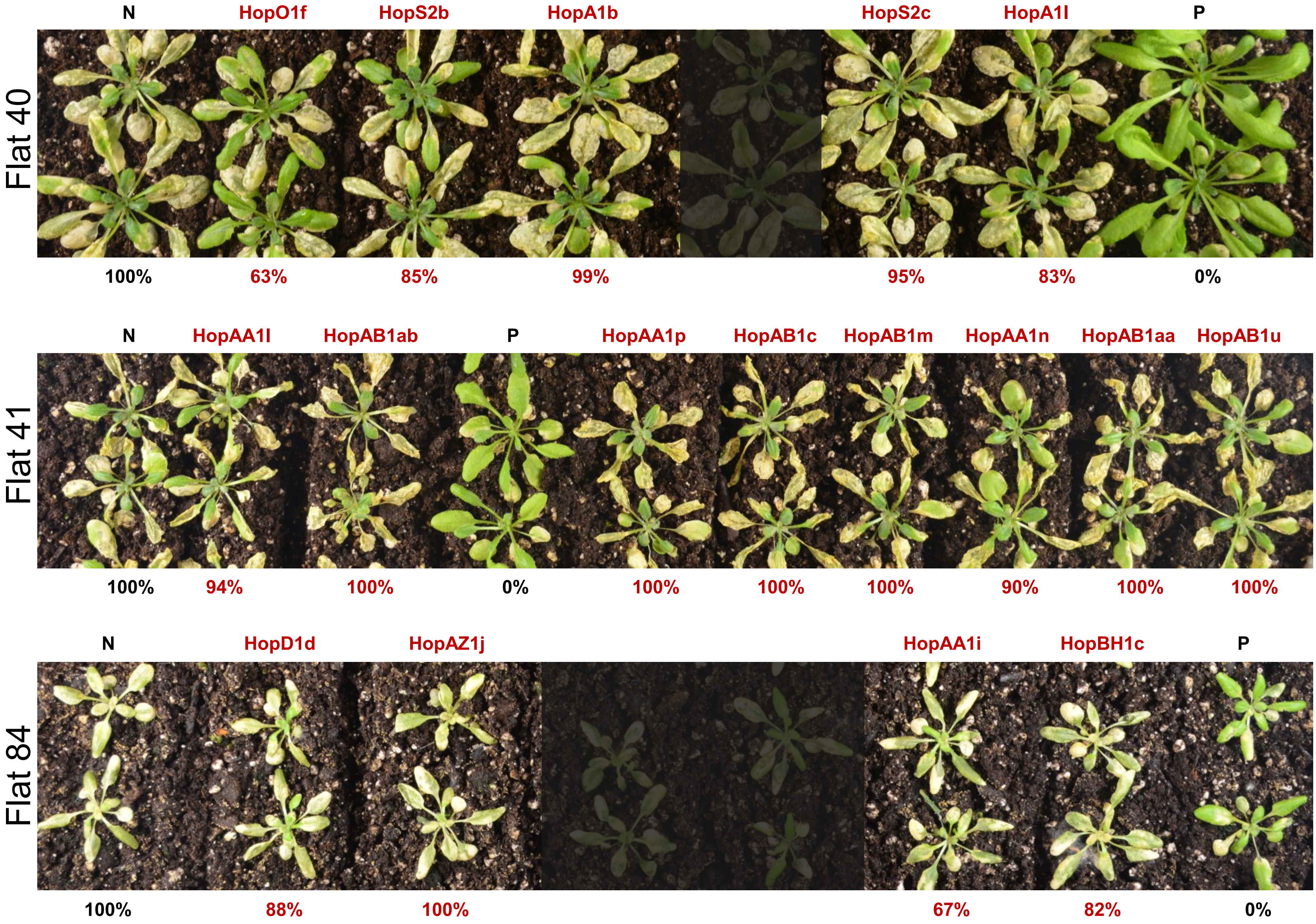
Flat 40
N
HopO1f
HopS2b
HopA1b
HopS2c
HopA1l
P
100%
63%
85%
99%
95%
83%
0%
Flat 41
N
HopAA1l
HopAB1ab
P
HopAA1p
HopAB1c
HopAB1m
HopAA1n
HopAB1aa
HopAB1u
100%
94%
100%
0%
100%
100%
100%
90%
100%
100%
Flat 84
N
HopD1d
HopAZ1j
HopAA1i
HopBH1c
P
100%
88%
100%
67%
82%
0%

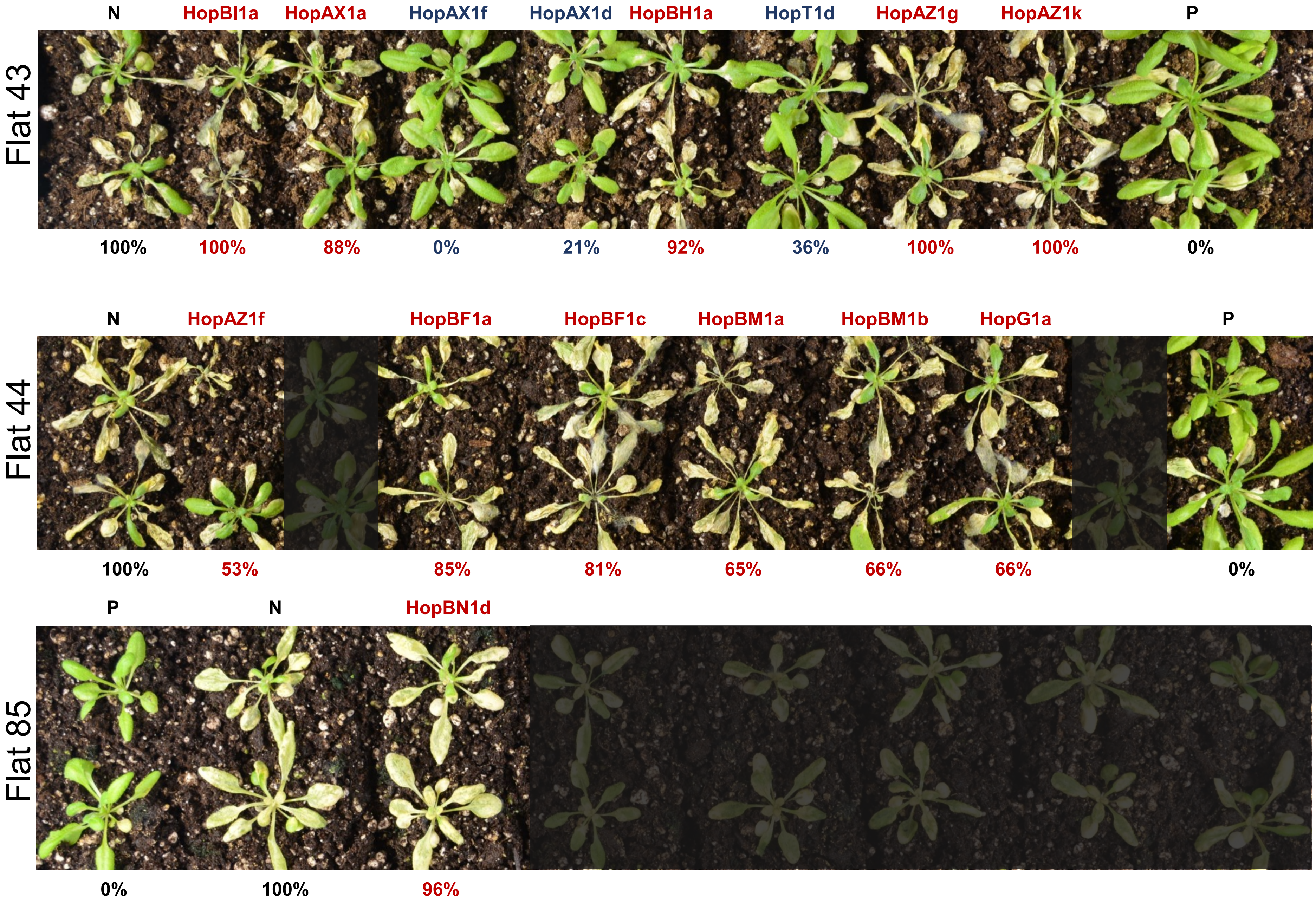

Flat 43
N
HopBI1a
HopAX1a
HopAX1f
HopAX1d
HopBH1a
HopT1d
HopAZ1g
HopAZ1k
P
100%
100%
88%
0%
21%
92%
36%
100%
100%
0%
Flat 44
N
HopAZ1f
HopBF1a
HopBF1c
HopBM1a
HopBM1b
HopG1a
P
100%
53%
85%
81%
65%
66%
66%
0%
Flat 85
P
N
HopBN1d
0%
100%
96%

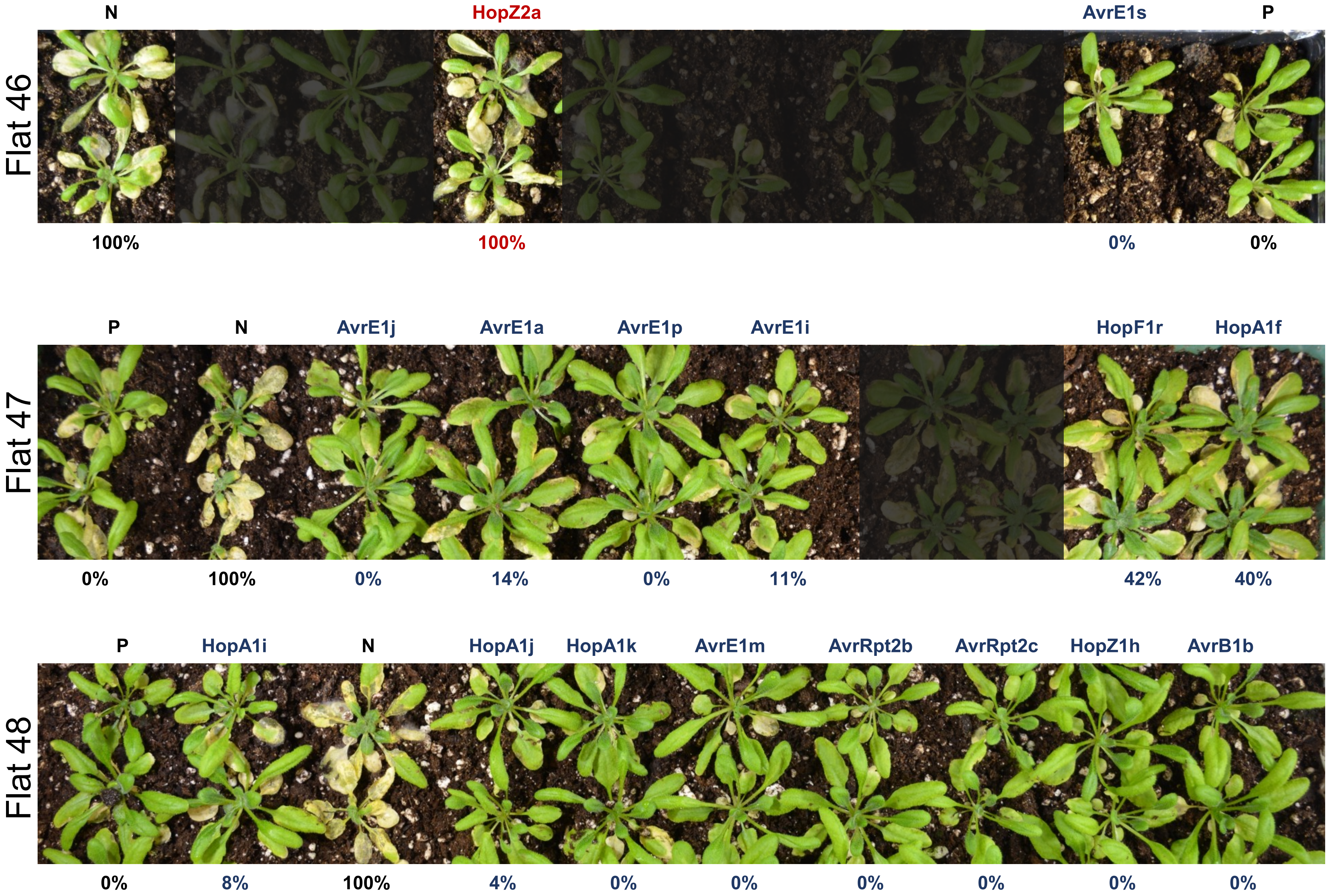
Flat 46
N
HopZ2a
AvrE1s
P
100%
100%
0%
0%
Flat 47
P
N
AvrE1j
AvrE1a
AvrE1p
AvrE1i
HopF1r
HopA1f
0%
100%
0%
14%
0%
11%
42%
40%
Flat 48
P
HopA1i
N
HopA1j
HopA1k
AvrE1m
AvrRpt2b
AvrRpt2c
HopZ1h
AvrB1b
0%
8%
100%
4%
0%
0%
0%
0%
0%
0%

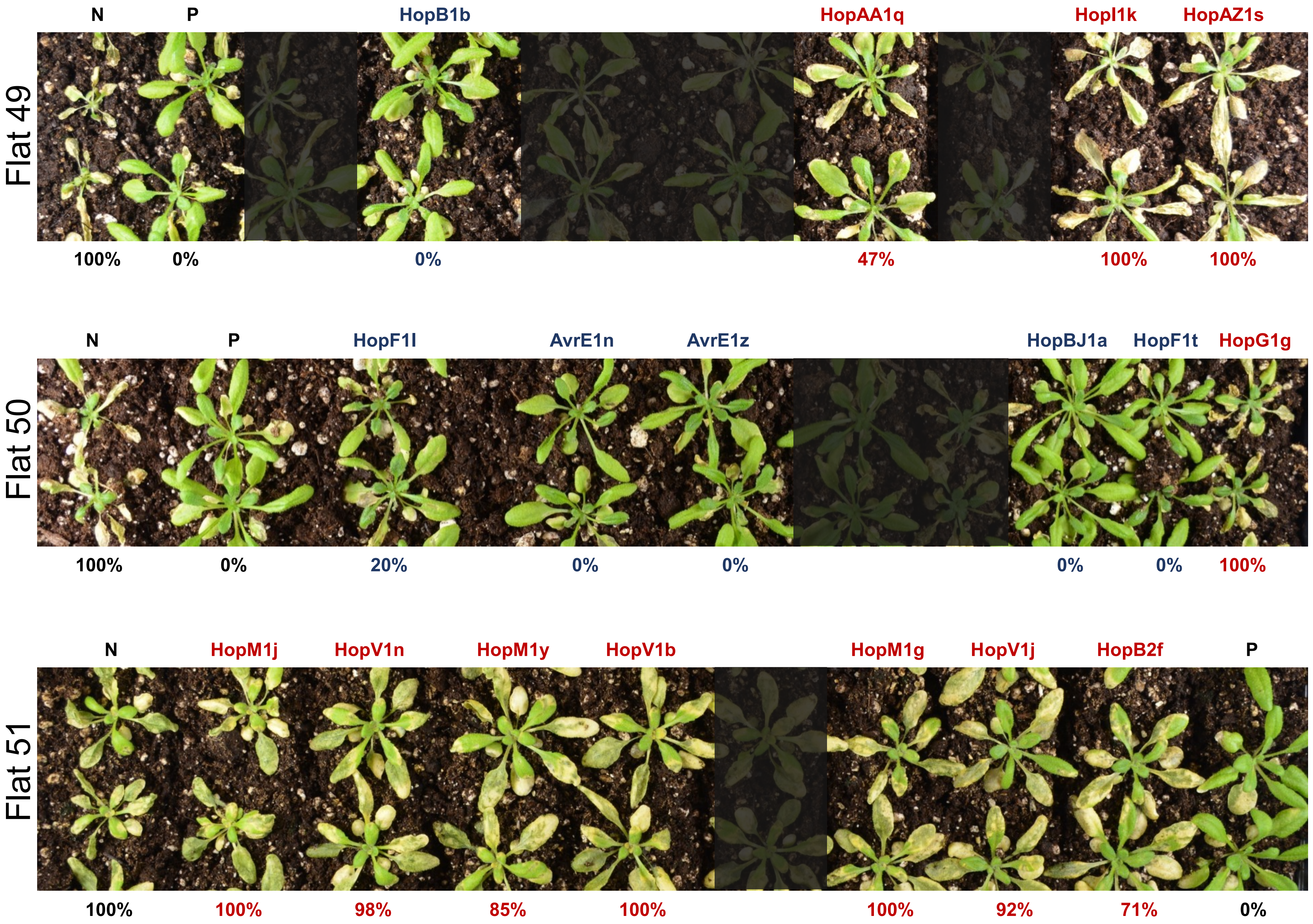
Flat 49
N
P
HopB1b
HopAA1q
HopI1k
HopAZ1s
100%
0%
0%
47%
100%
100%
Flat 50
N
P
HopF1l
AvrE1n
AvrE1z
HopBJ1a
HopF1t
HopG1g
100%
0%
20%
0%
0%
0%
0%
100%
Flat 51
N
HopM1j
HopV1n
HopM1y
HopV1b
HopM1g
HopV1j
HopB2f
P
100%
100%
98%
85%
100%
100%
92%
71%
0%

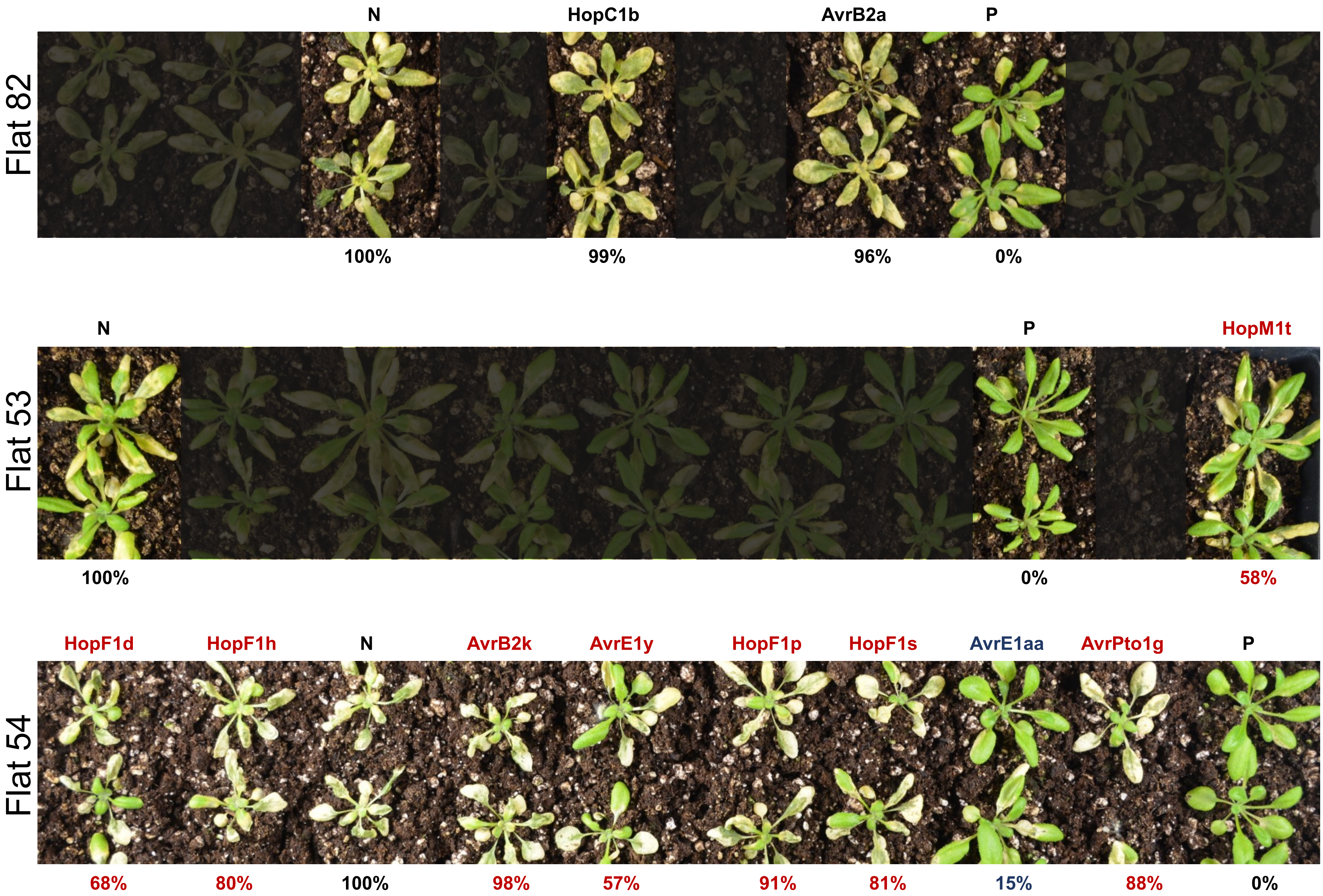

Flat 82
N
HopC1b
AvrB2a
P
100%
99%
96%
0%
Flat 53
N
P
HopM1t
100%
0%
58%
Flat 54
HopF1d
HopF1h
N
AvrB2k
AvrE1y
HopF1p
HopF1s
AvrE1aa
AvrPto1g
P
68%
80%
100%
98%
57%
91%
81%
15%
88%
0%

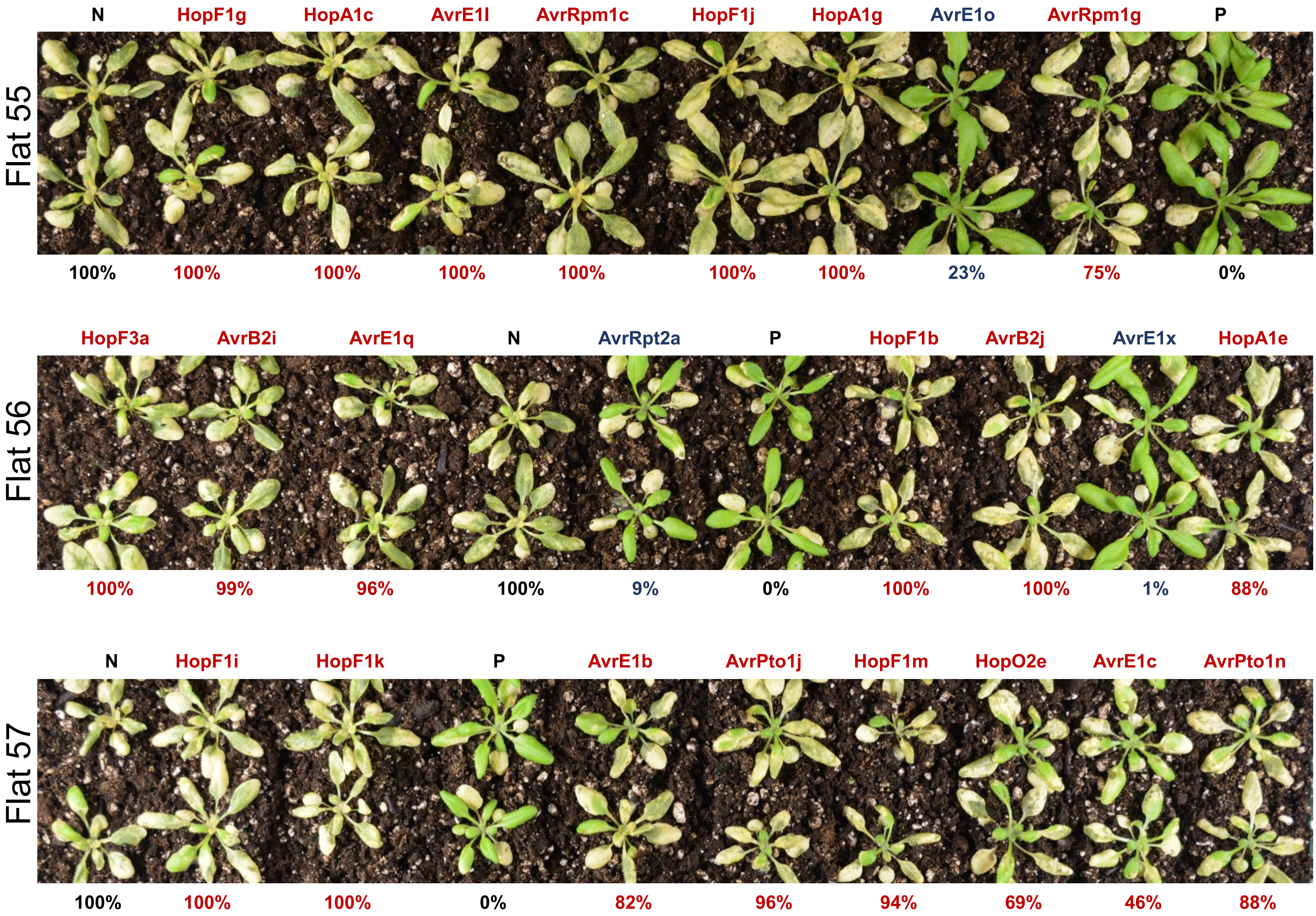

Flat 55
N
HopF1g
HopA1c
AvrE1l
AvrRpm1c
HopF1j
HopA1g
AvrE1o
AvrRpm1g
P
100%
100%
100%
100%
100%
100%
100%
23%
75%
0%
Flat 56
HopF3a
AvrB2i
AvrE1q
N
AvrRpt2a
P
HopF1b
AvrB2j
AvrE1x
HopA1e
100%
99%
96%
100%
9%
0%
100%
100%
1%
88%
Flat 57
N
HopF1i
HopF1k
P
AvrE1b
AvrPto1j
HopF1m
HopO2e
AvrE1c
AvrPto1n
100%
100%
100%
0%
82%
96%
94%
69%
46%
88%

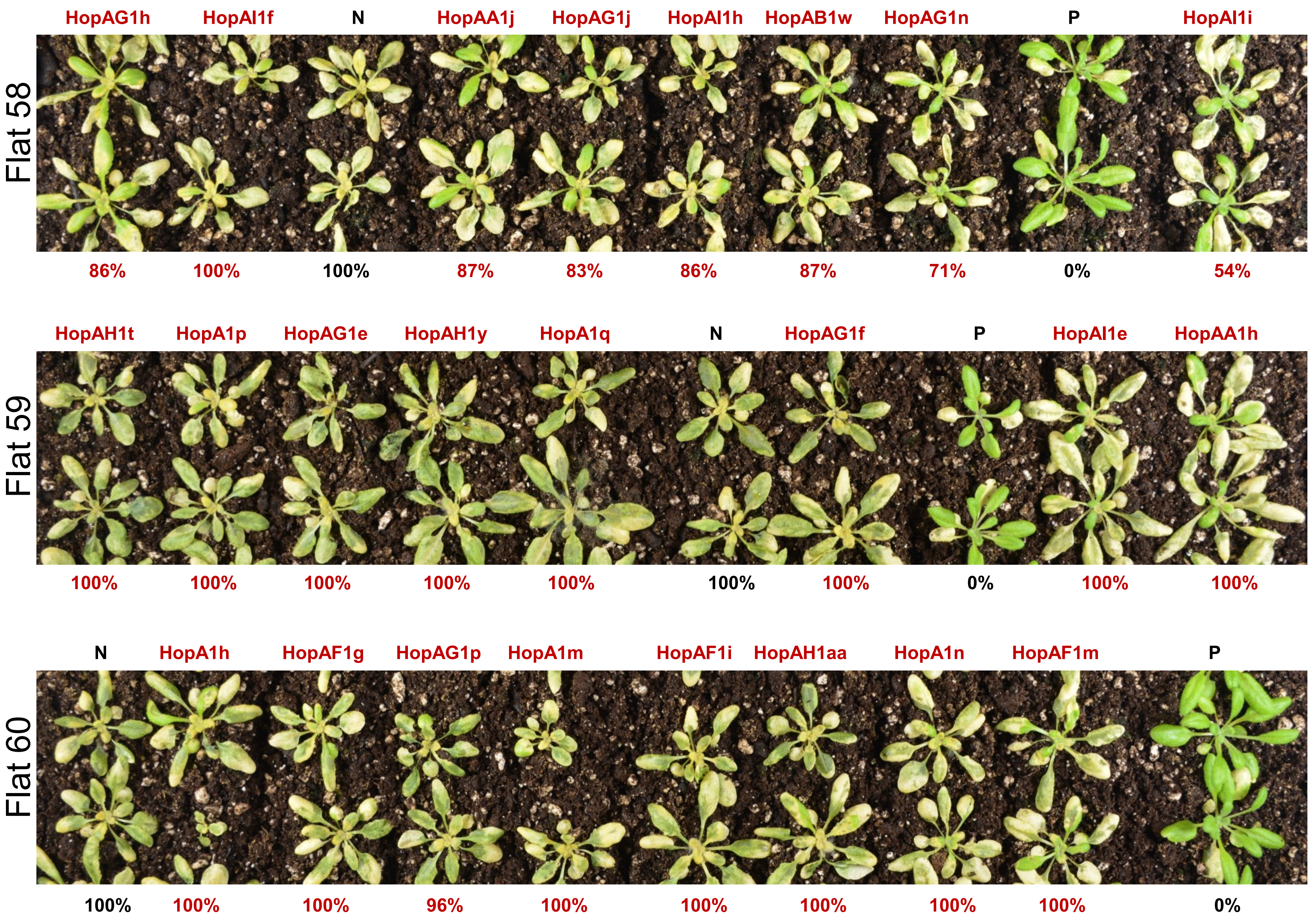
Flat 58
HopAG1h
HopAI1f
N
HopAA1j
HopAG1j
HopAI1h
HopAB1w
HopAG1n
P
HopAI1i
86%
100%
100%
87%
83%
86%
87%
71%
0%
54%
Flat 59
HopAH1t
HopA1p
HopAG1e
HopAH1y
HopA1q
N
HopAG1f
P
HopAI1e
HopAA1h
100%
100%
100%
100%
100%
100%
100%
0%
100%
100%
Flat 60
N
HopA1h
HopAF1g
HopAG1p
HopA1m
HopAF1i
HopAH1aa
HopA1n
HopAF1m
P
100%
100%
100%
96%
100%
100%
100%
100%
100%
0%

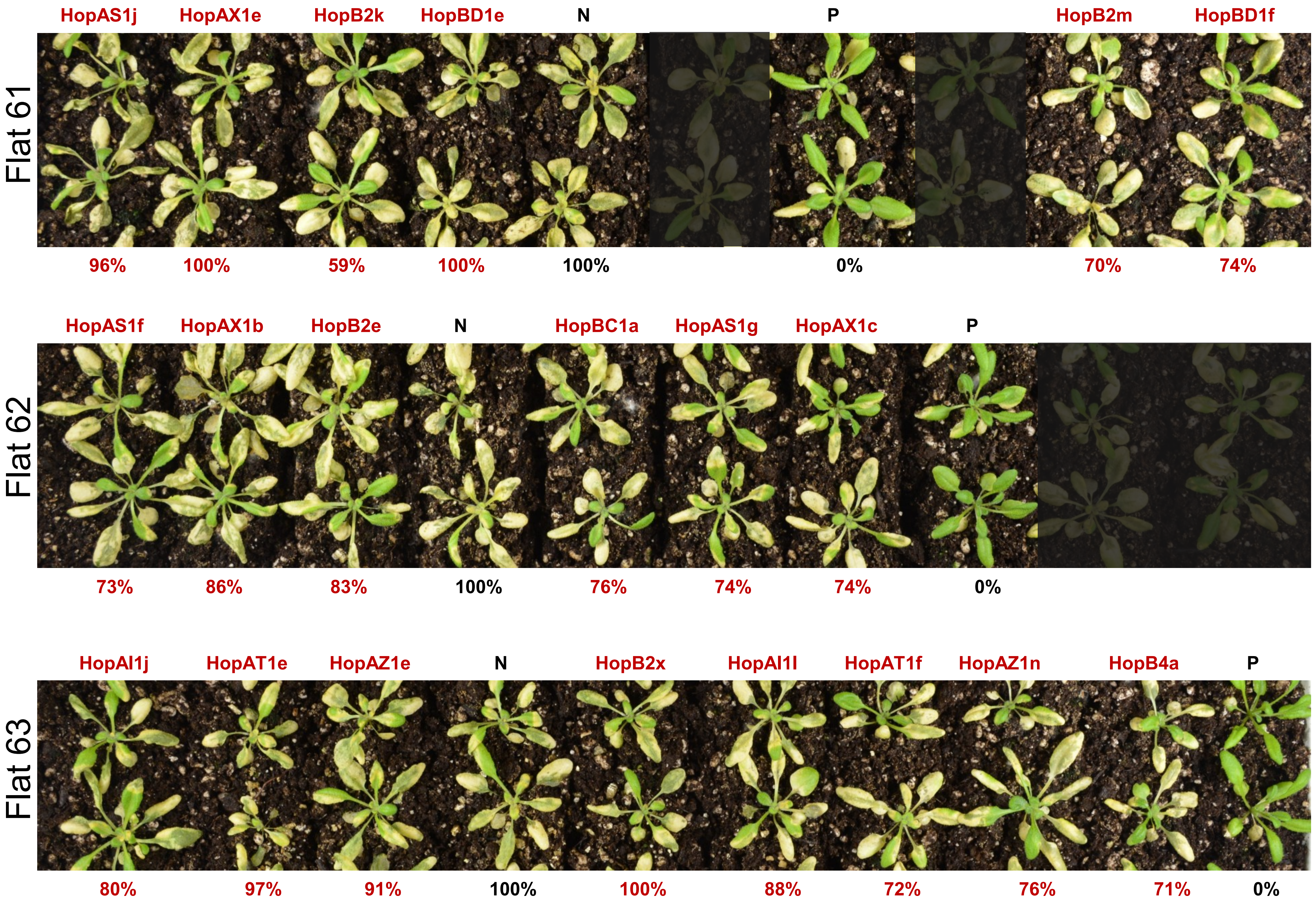
Flat 61
HopAS1j
HopAX1e
HopB2k
HopBD1e
N
P
HopB2m
HopBD1f
96%
100%
59%
100%
100%
0%
70%
74%
Flat 62
HopAS1f
HopAX1b
HopB2e
N
HopBC1a
HopAS1g
HopAX1c
P
73%
86%
83%
100%
76%
74%
74%
0%
Flat 63
HopAI1j
HopAT1e
HopAZ1e
N
HopB2x
HopAI1l
HopAT1f
HopAZ1n
HopB4a
P
80%
97%
91%
100%
100%
88%
72%
76%
71%
0%

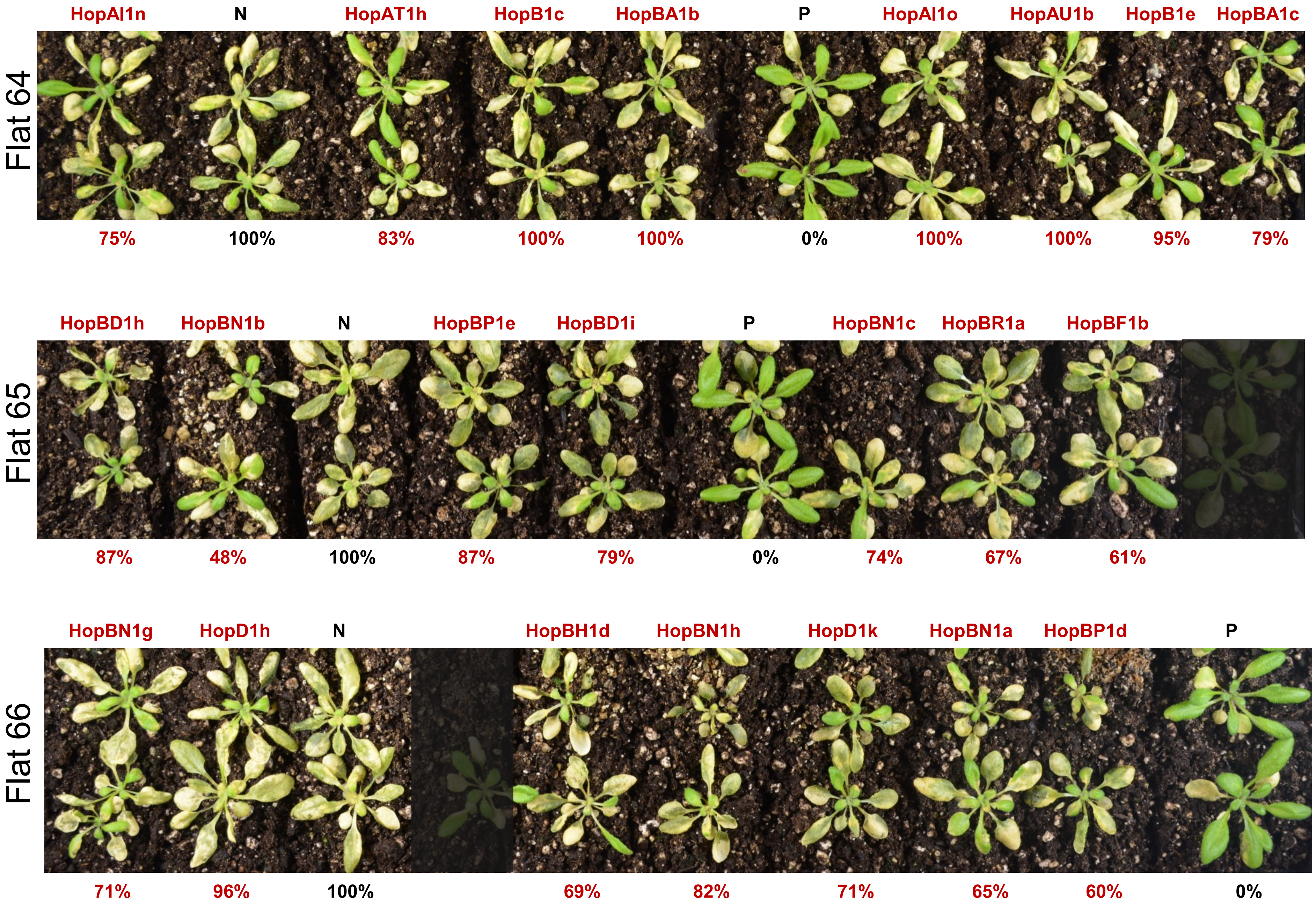
Flat 64
HopAI1n
N
HopAT1h
HopB1c
HopBA1b
P
HopAI1o
HopAU1b
HopB1e
HopBA1c
75%
100%
83%
100%
100%
0%
100%
100%
95%
79%
Flat 65
HopBD1h
HopBN1b
N
HopBP1e
HopBD1i
P
HopBN1c
HopBR1a
HopBF1b
87%
48%
100%
87%
79%
0%
74%
67%
61%
Flat 66
HopBN1g
HopD1h
N
HopBH1d
HopBN1h
HopD1k
HopBN1a
HopBP1d
P
71%
96%
100%
69%
82%
71%
65%
60%
0%

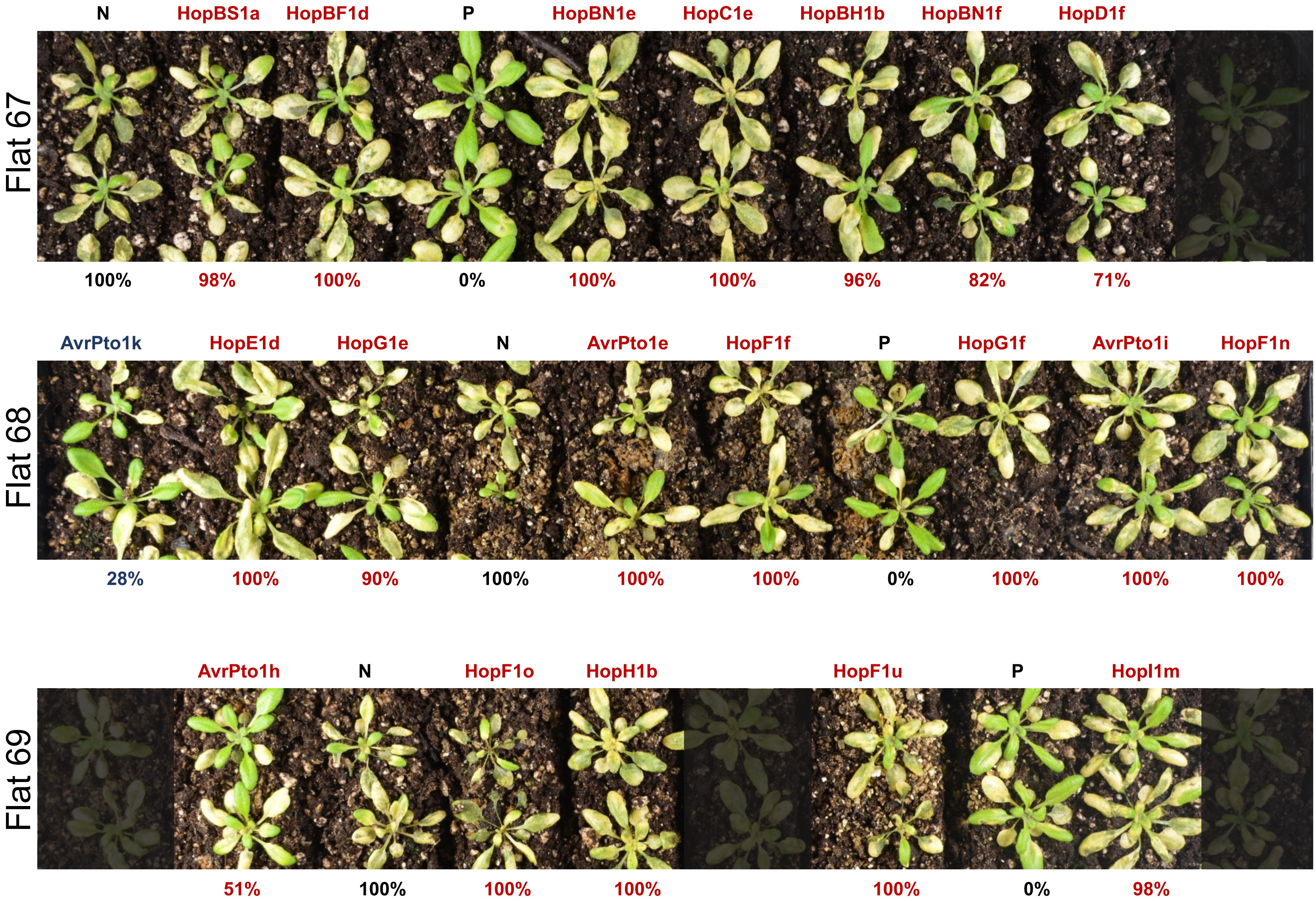
Flat 67
N
HopBS1a
HopBF1d
P
HopBN1e
HopC1e
HopBH1b
HopBN1f
HopD1f
100%
98%
100%
0%
100%
100%
96%
82%
71%
Flat 68
AvrPto1k
HopE1d
HopG1e
N
AvrPto1e
HopF1f
P
HopG1f
AvrPto1i
HopF1n
28%
100%
90%
100%
100%
100%
0%
100%
100%
100%
Flat 69
AvrPto1h
N
HopF1o
HopH1b
HopF1u
P
HopI1m
51%
100%
100%
100%
100%
0%
98%

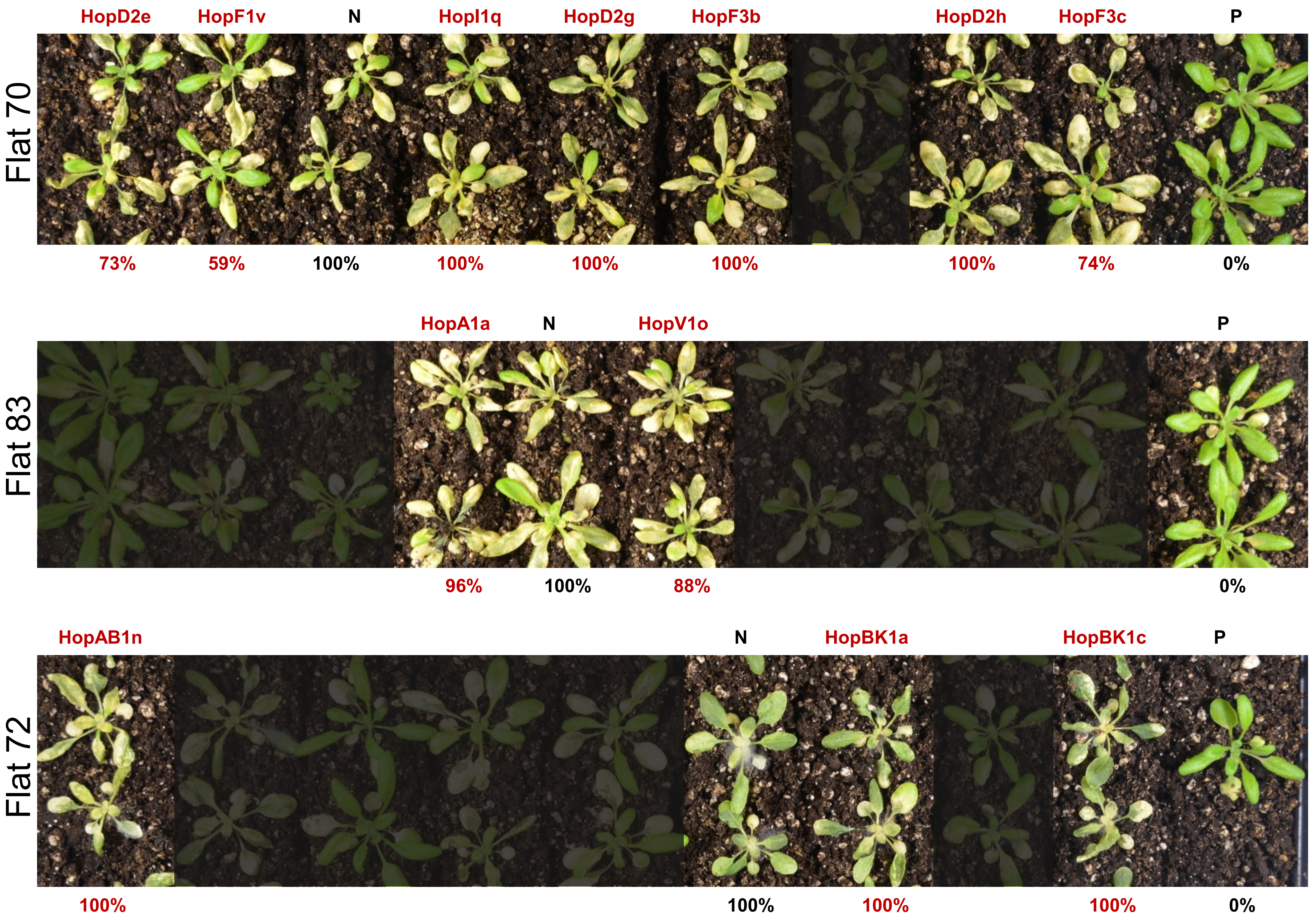
Flat 70
HopD2e
HopF1v
N
HopI1q
HopD2g
HopF3b
HopD2h
HopF3c
P
73%
59%
100%
100%
100%
100%
100%
74%
0%
Flat 83
HopA1a
N
HopV1o
P
96%
100%
88%
0%
Flat 72
HopAB1n
N
HopBK1a
HopBK1c
P
100%
100%
100%
100%
0%

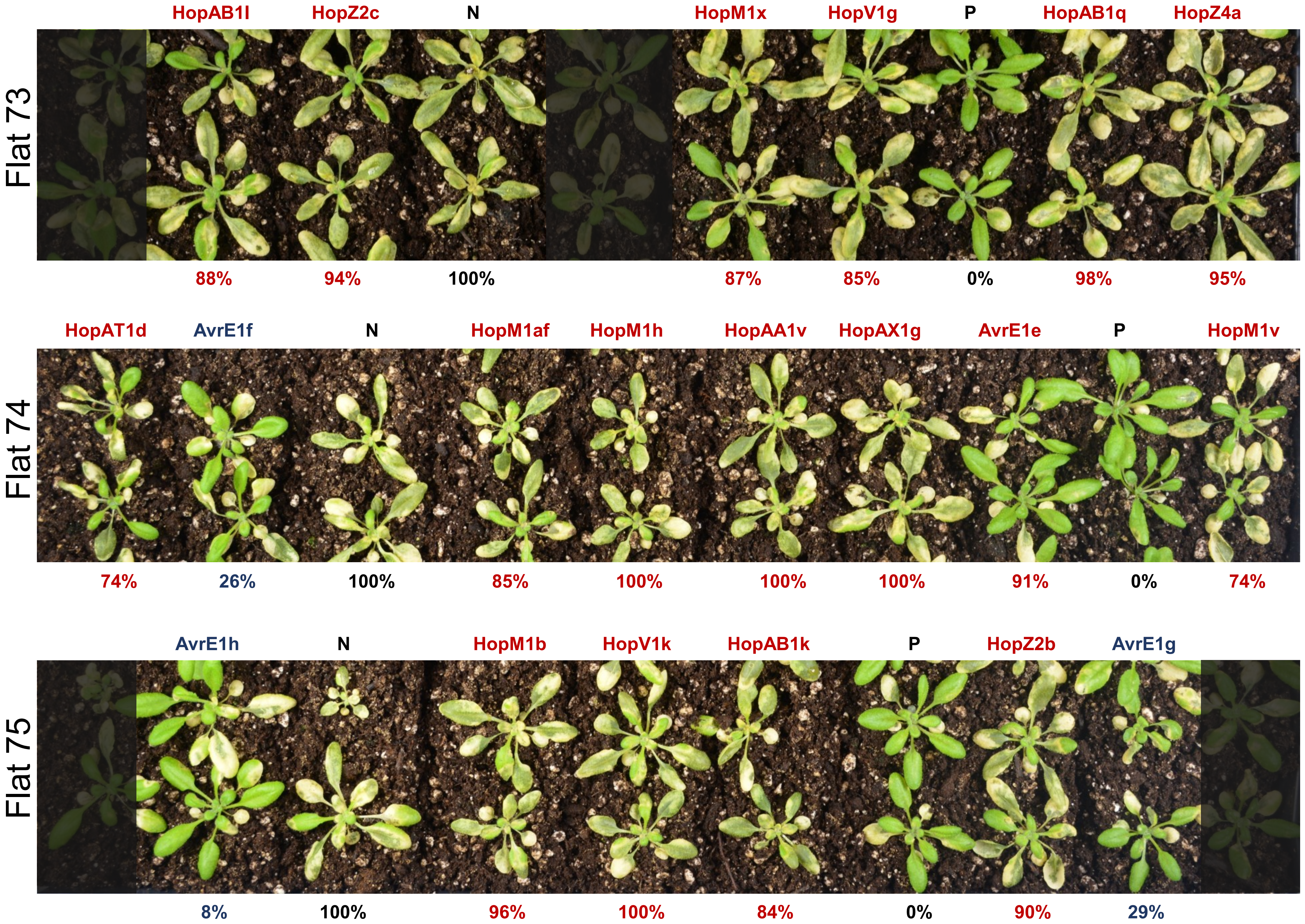
Flat 73
HopAB1l
HopZ2c
N
HopM1x
HopV1g
P
HopAB1q
HopZ4a
88%
94%
100%
87%
85%
0%
98%
95%
Flat 74
HopAT1d
AvrE1f
N
HopM1af
HopM1h
HopAA1v
HopAX1g
AvrE1e
P
HopM1v
74%
26%
100%
85%
100%
100%
100%
91%
0%
74%
Flat 75
AvrE1h
N
HopM1b
HopV1k
HopAB1k
P
HopZ2b
AvrE1g
8%
100%
96%
100%
84%
0%
90%
29%

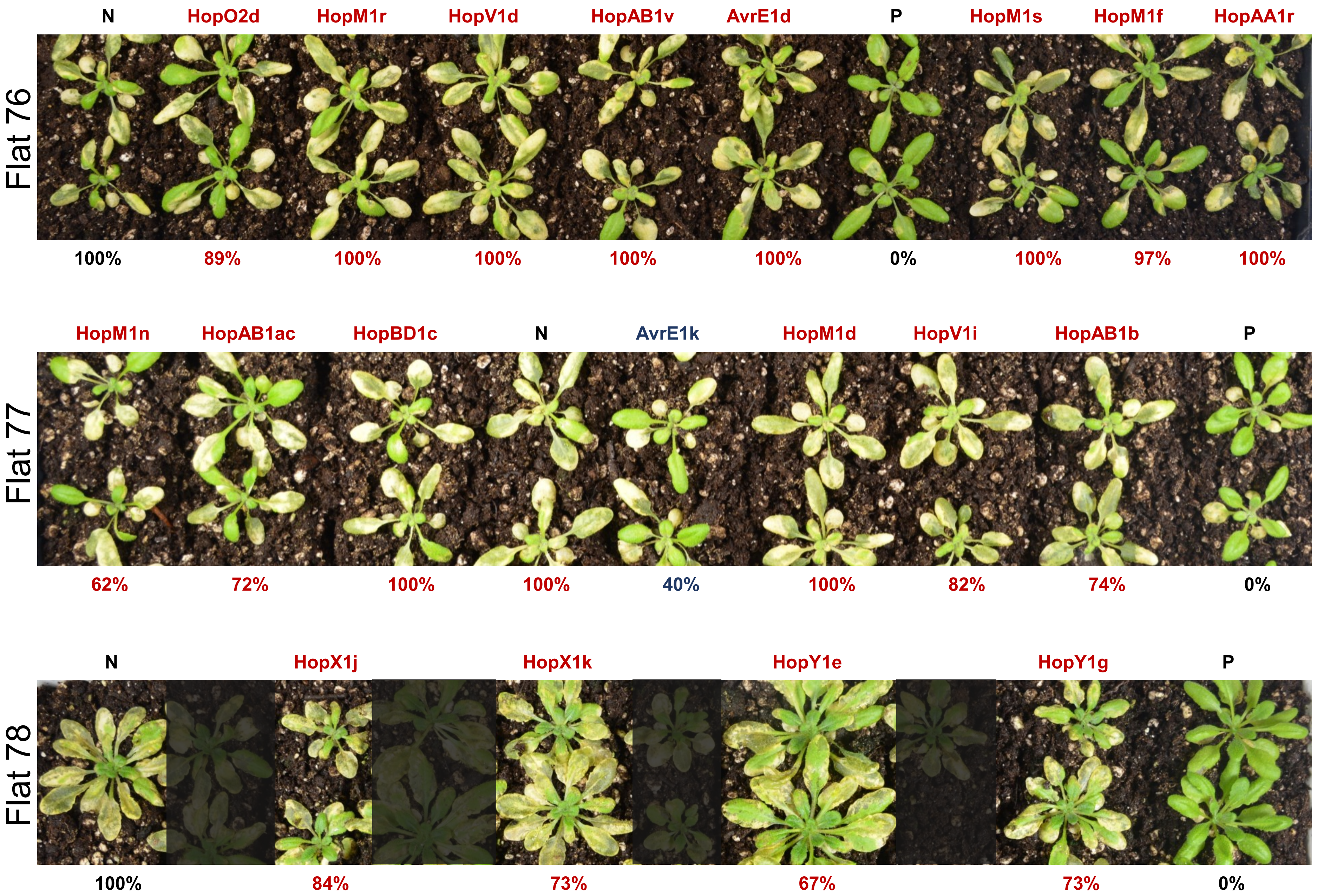
Flat 76
N
HopO2d
HopM1r
HopV1d
HopAB1v
AvrE1d
P
HopM1s
HopM1f
HopAA1r
100%
89%
100%
100%
100%
100%
0%
100%
97%
100%
Flat 77
HopM1n
HopAB1ac
HopBD1c
N
AvrE1k
HopM1d
HopV1i
HopAB1b
P
62%
72%
100%
100%
40%
100%
82%
74%
0%
Flat 78
N
HopX1j
HopX1k
HopY1e
HopY1g
P
100%
84%
73%
67%
73%
0%

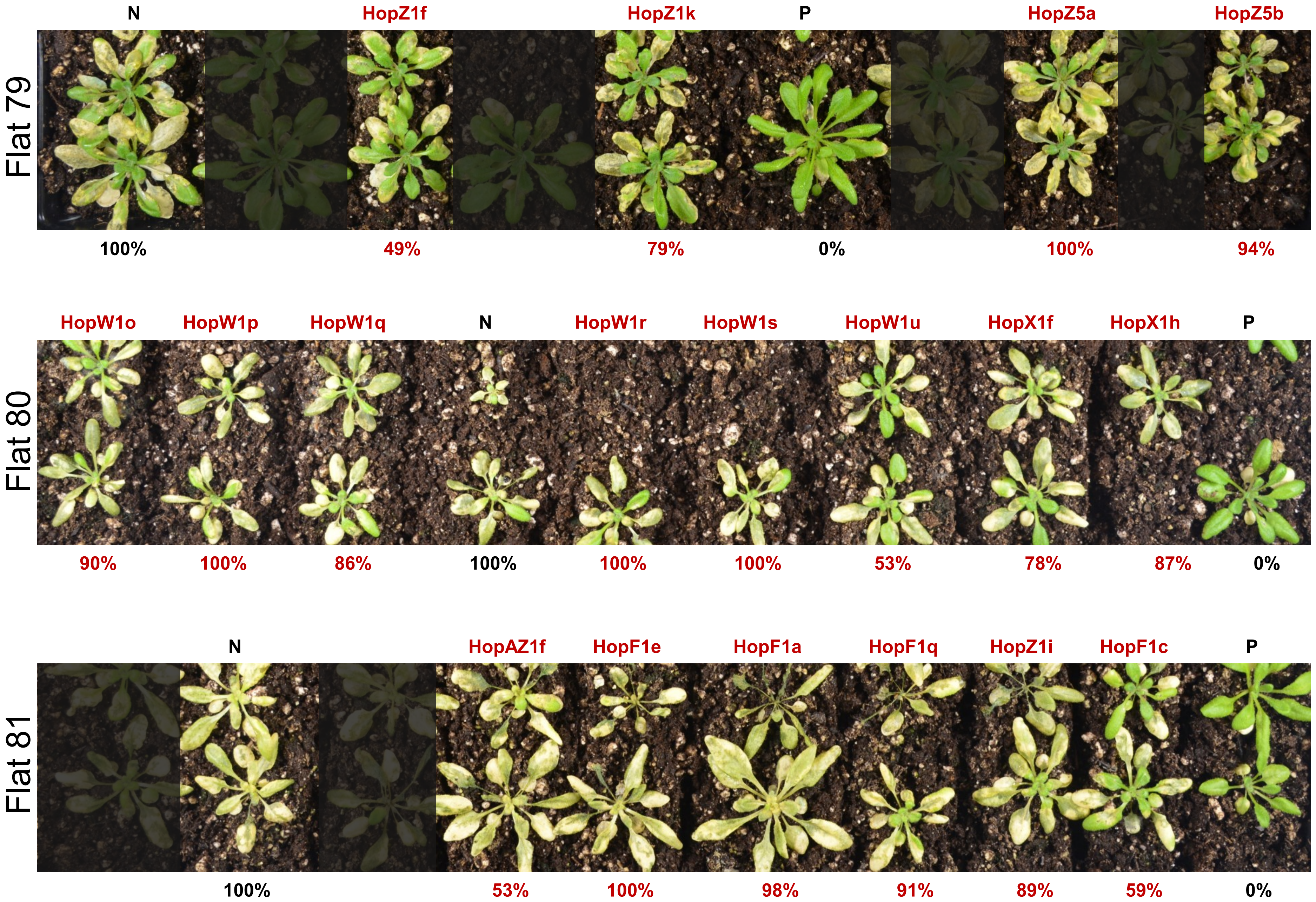

Flat 79
N
HopZ1f
HopZ1k
P
HopZ5a
HopZ5b
100%
49%
79%
0%
100%
94%
Flat 80
HopW1o
HopW1p
HopW1q
N
HopW1r
HopW1s
HopW1u
HopX1f
HopX1h
P
90%
100%
86%
100%
100%
100%
53%
78%
87%
0%
Flat 81
N
HopAZ1f
HopF1e
HopF1a
HopF1q
HopZ1i
HopF1c
P
100%
53%
100%
98%
91%
89%
59%
0%

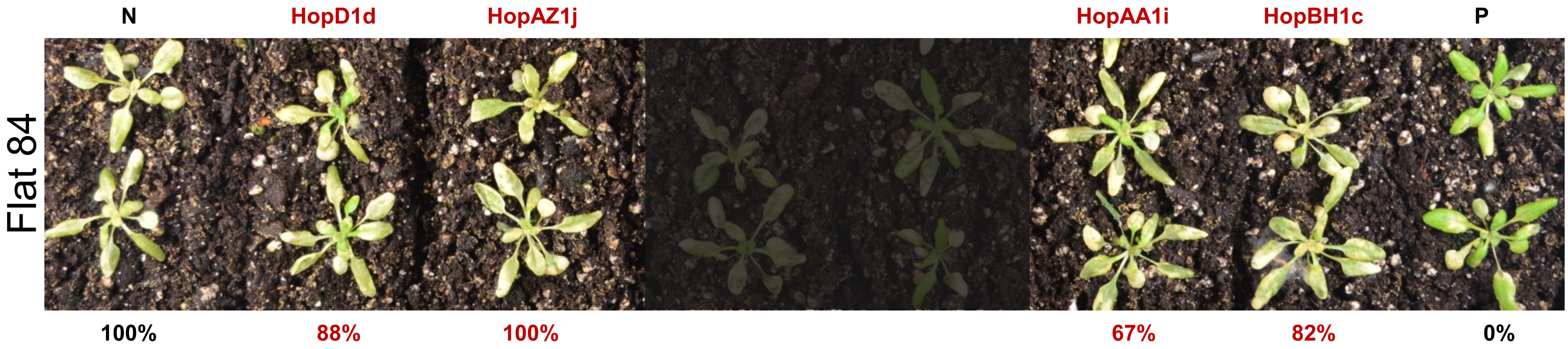

N
HopD1d
HopAZ1j
HopAA1i
HopBH1c
P
Flat 84
100%
88%
100%
67%
82%
0%
