# Supplementary material for: Metaeffector interactions modulate the type III effector-triggered immunity load of Pseudomonas syringae
Source: PLoS Pathog. 2022 May 16;18(5):e1010541. doi: 10.1371/journal.ppat.1010541 (PMC9135338; doi:10.1371/journal.ppat.1010541)
Supplement: S10 Fig — Hypersensitive response (HR) assays of PmaES4326 delivering an empty-vector (EV), AvrRpt2, and ETI-elicitorΔ79avrRpt2 fusions with and without their corresponding ETI-suppressors. The open reading frames of AvrPto1m and HopT1b (alongside upstream promoters and without stop codons) were cloned into the Gateway-compatible pBBR1-MCS2 vector which fuses the effectors to the C-terminus of AvrRpt2 [44–46]. These ETI-elicitorΔ79avrRpt2 fusions were paired with empty pUCP20 vector, or pUCP20 carrying the suppressors of each effector. These experiments were performed in triplicate. (PDF) [file ppat.1010541.s014.pdf]

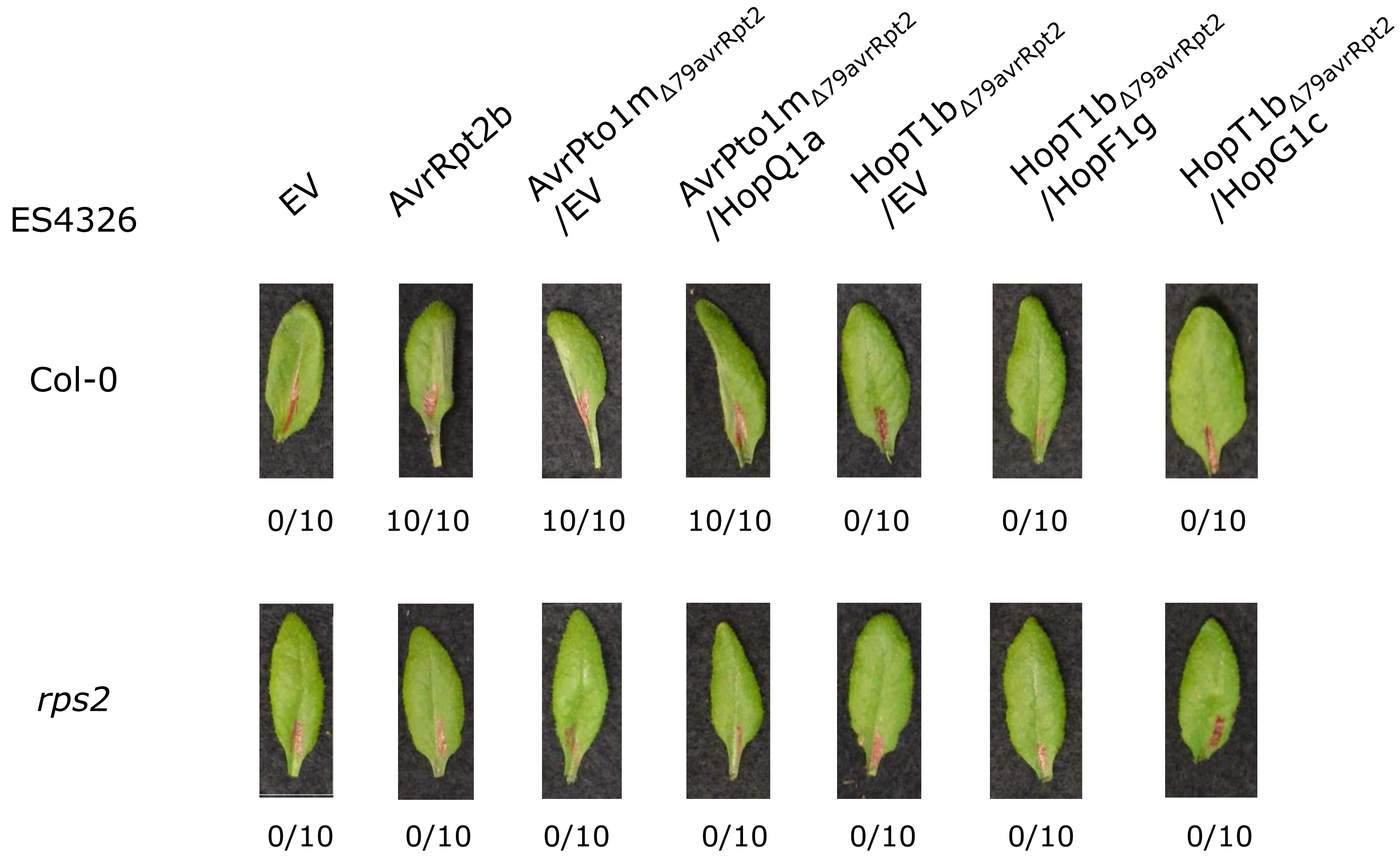

ES4326
EV
AvrRpt2b
AvrPto1m$_{\Delta79avrRpt2}$ /EV
AvrPto1m$_{\Delta79avrRpt2}$ /HopQ1a
HopT1b$_{\Delta79avrRpt2}$ /EV
HopT1b$_{\Delta79avrRpt2}$ /HopF1g
HopT1b$_{\Delta79avrRpt2}$ /HopG1c
Col-0
0/10
10/10
10/10
10/10
0/10
0/10
0/10
rps2
0/10
0/10
0/10
0/10
0/10
0/10
0/10
